# Designing Nontrivial Real‐Space Berry Curvature through Non‐Monotonic Bulk Inversion Symmetry Breaking in Self‐Intercalated Cr_1+δ_Te_2_

**DOI:** 10.1002/smsc.202500028

**Published:** 2025-03-24

**Authors:** Seungwon Rho, Dameul Jeong, Hyeong‐Ryul Kim, Jaeseok Huh, Hyeong‐Jun Son, Young‐Kyun Kwon, Mann‐Ho Cho

**Affiliations:** ^1^ Department of Physics Yonsei University Seoul 03722 Republic of Korea; ^2^ Department of Physics and Research Institute for Basic Sciences Kyung Hee University Seoul 02447 Republic of Korea; ^3^ Department of Information Display Kyung Hee University Seoul 02447 Republic of Korea; ^4^ Department of System Semiconductor Engineering Yonsei University Seoul 03722 Republic of Korea

**Keywords:** 2D ferromagnetic materials, chiral spin textures, Dzyaloshinskii–Moriya interactions, spintronics, topological Hall effects

## Abstract

The real‐space Berry curvature ($\Omega_{r}$) in magnetic materials has gained significant attention for its potential applications in chiral spintronic devices. $\Omega_{r}$ manifests in chiral spin textures stabilized by the Dzyaloshinskii–Moriya interaction (DMI), which arises in inversion‐asymmetric systems. Herein, the topological Hall effect (THE) in 2D ferromagnet Cr_1+δ_Te_2_ as a function of the Cr intercalant (*δ*) is investigated. A nonlinear dependence of the THE amplitude induced by $\Omega_{r}$ on *δ* is identified, originating from non‐monotonic bulk inversion symmetry breaking via Cr self‐intercalation. Density‐functional theory calculations further reveal a strong correlation between THE amplitude and bulk DMI strength (*E*_DMI_), demonstrating both the mechanism of THE and the tunability of $\Omega_{r}$ in Cr_1+δ_Te_2_. Remarkably, Cr_1.612_Te_2_ exhibits the largest THE amplitude observed to date (2.75 μΩ⋅cm) in the Cr_1+δ_Te_2_ family, which is a strong candidate for the highest THE amplitude, given its magnetic anisotropy and *E*_DMI_. Overall, by confirming the critical role of bulk DMI and magnetic anisotropy in engineering $\Omega_{r}$, the most efficient strategy for designing $\Omega_{r}$ in 2D ferromagnetic materials through atomic‐scale self‐intercalation is proposed. These findings provide fundamental insights into the relationship between *E*_DMI_ and THE in Cr_1+δ_Te_2_ and offer a promising approach for designing high‐performance chiral spintronic devices.

## Graphical abstract

By controlling the Cr intercalant (*δ*) in Cr_1+δ_Te_2_, the topological Hall effect (THE) amplitude ($\rho_{\text{THE}}^{\text{peak}}$) and Dzyaloshinskii–Moriya interaction strength can be tuned, revealing a strong correlation between them. Moreover, the largest $\rho_{\text{THE}}^{\text{peak}}$ of 2.75 μΩ⋅cm is observed at δ= 0.612. These findings elucidate the mechanism underlying the THE, providing an effective strategy for designing high‐performance chiral spintronic devices.

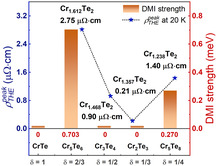

## 1 Introduction

The real‐space Berry curvature ($\Omega_{r}$), representing scalar spin chirality defined on chiral spin textures such as magnetic skyrmion, has recently been recognized as an extension of *k*‐space Berry curvature with practical applications.^[^ ^1^, ^2^, ^3^, ^4^, ^5^ ^]^ For instance, magnetic skyrmion, which is energetically stable, has great potential for applications in high‐density data storage devices and racetrack memory.^[^ ^6^, ^7^ ^]^ Chiral spin textures have been observed in various material systems such as oxide films,^[^ ^8^, ^9^, ^10^, ^11^ ^]^ B20‐type compounds,^[^ ^12^, ^13^, ^14^ ^]^ and low‐dimensional ferromagnetic materials (FMs).^[^ ^15^, ^16^, ^17^, ^18^ ^]^ Particularly, 2D FMs, which facilitate diverse control over spin textures, are expected to play a significant role in the development of high‐efficiency and low‐power spintronic devices.

To stabilize chiral spin textures within magnetic materials, the Dzyaloshinskii–Moriya interaction (DMI) term is required alongside the Heisenberg exchange interaction term in the magnetization energy Hamiltonian. DMI, which arises due to inversion symmetry breaking, induces chiral spin textures through its competition with exchange interaction energy. In heavy metal/2D FM heterostructures, where inversion symmetry is broken at the interface, the creation of magnetic skyrmions stabilized by interfacial DMI (iDMI) has been reported.^[^ ^19^, ^20^ ^]^ However, exploration of non‐centrosymmetric magnetic materials, where bulk DMI emerges, remains limited, with a lack of rigorous study on the chiral spin textures of such materials. These materials can exhibit Néel‐type skyrmions or noncoplanar spin textures of which spin rotation symmetry is broken, depending on their point group symmetry.^[^ ^17^ ^]^ For materials with chiral spin textures, $\Omega_{r}$ influences electron transport by imparting a fictitious magnetic field to electrons, detectable through Hall measurements. Magnetic materials exhibiting chiral spin textures produce a topological Hall effect (THE) signal, which is proportional to $\Omega_{r}$, alongside the ordinary Hall effect (OHE) and anomalous Hall effect (AHE). This phenomenon, where THE appears as a hump‐like signal in the Hall resistance, is a critical feature in materials with $\Omega_{r}$ originating from chiral spin textures.^[^ ^21^, ^22^ ^]^

Recent research on 2D FMs has highlighted Cr_1+δ_Te_2_ as a promising system, allowing Cr self‐intercalation to modulate its magnetic properties.^[^ ^23^, ^24^, ^25^, ^26^, ^27^, ^28^ ^]^ Although reports have discussed chiral spin textures in the Cr_1+δ_Te_2_ system,^[^ ^15^, ^17^, ^29^, ^30^, ^31^, ^32^ ^]^ ongoing debates exist regarding its inversion symmetry, depending on the extent of self‐intercalation. Therefore, by precisely controlling the amount of Cr intercalants in Cr_1+δ_Te_2_, we can better understand non‐centrosymmetric configurations and optimize the generation of $\Omega_{r}$.^[^ ^17^, ^33^ ^]^

In this study, we fabricated Cr_1+δ_Te_2_ with various *δ* values using molecular beam epitaxy (MBE) to investigate THE as influenced by self‐intercalated Cr atoms. We observed that THE amplitude is highly sensitive to changes in *δ*, suggesting that Cr intercalation can tune $\Omega_{r}$ in Cr_1+δ_Te_2_. Specifically, we report a giant THE resistivity amplitude of 2.75 μΩ⋅cm in Cr_1.612_Te_2_ at 20 K, the highest value among Cr_1+δ_Te_2_ systems, originating from its noncoplanar spin texture. Furthermore, as *δ* decreases from 0.612, the THE amplitude decreases until reaching δ≈ 1/3 (Cr_2_Te_3_, known as a centrosymmetric crystal), and it disappears. It then reappears with significant amplitude as *δ* diminished further than 1/3. Our density‐functional theory (DFT) calculations confirm that THE amplitude, which is proportional to $\Omega_{r}$, in Cr_1+δ_Te_2_ is governed by the crystal symmetry‐dependent bulk DMI strength, providing insight into a straightforward approach to designing chiral spintronic devices via Cr self‐intercalation. Furthermore, we identified that among the physical mechanisms for generating $\Omega_{r}$, including iDMI, bulk DMI is the most efficient way for controlling $\Omega_{r}$ as well as strategy for engineering $\Omega_{r}$ in 2D FMs. Our study pioneers the direct modulation of $\Omega_{r}$ through precise Cr intercalation, establishing a novel approach to controlling $\Omega_{r}$ for future chiral spintronic applications.

## 2 Chiral Spin Texture in 2D FM Cr_1+δ_Te_2_ and Sample Characterization

**Figure 1:**
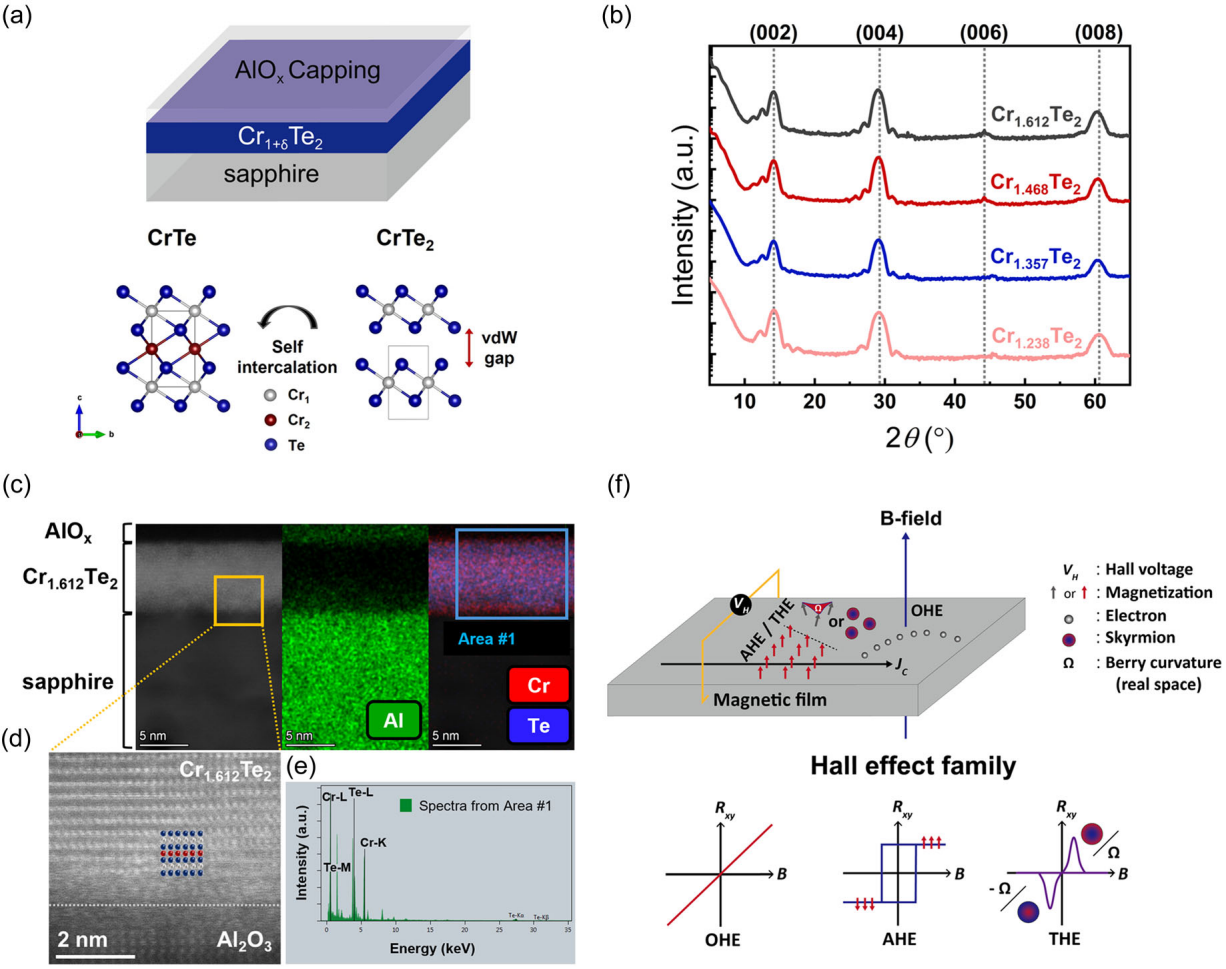
Experimental scheme and characterization of Cr_1+δ_Te_2_. a) A schematic of the experimental layout (top panel) and the crystal structure of Cr_1+δ_Te_2_ (bottom panel). b) XRD spectra of AlO_*x*_/Cr_1+δ_Te_2_ with δ= 0.612, 0.468, 0.357, and 0.238. c) Cross‐sectional HAADF–STEM image (left panel) and EDS elemental mapping data (middle and right panels) of AlO_*x*_ 2 nm/Cr_1+δ_Te_2_ 7.6 nm/sapphire with δ= 0.612. d) Enlarged high‐resolution STEM image of the yellow box in the left panel of (c). Each atom is identified with a different color according to the crystal structure (Cr: gray and red spheres, Te: blue spheres). e) EDS spectra obtained from Area #1, represented in the right panel of (c). f) A schematic of the Hall effect family.

Transition metal dichalcogenides have a layered structure with a van der Waals (vdW) gap, allowing the intercalation of atoms between layers. In the case of Cr_1+δ_Te_2_ with CrTe_2_ as the backbone, varying *δ* leads to different structures through the formation of intercalated bonding between vdW gaps (e.g., Cr_3_Te_4_, Cr_2_Te_3_, Cr_5_Te_8_), as illustrated in **Figure** **1**a (bottom panel). In particular, Cr_1+δ_Te_2_ with specific δ exhibits distinct crystal structures and magnetic properties.^[^ ^28^ ^]^ Notably, Cr_2_Te_3_ is known to possess an inversion‐symmetric crystal structure,^[^ ^34^ ^]^ denoted as P$\bar{3}$1c when Cr atoms are intercalated into the vdW gap at 33.3% occupancy. In contrast, it has been reported to adopt an inversion‐asymmetric (non‐centrosymmetric) P3m1 structure when Cr atoms are intercalated with slightly more or less occupancy,^[^ ^17^ ^]^ indicating that DMI can emerge, leading to the stabilization of chiral spin textures. Thus, with the emergence of chiral spin textures, THE can be detected due to the manifestation of nontrivial $\Omega_{r}$.^[^ ^21^, ^22^ ^]^ Although the phase characterization is not well interpreted, we investigated how differently THE appears according to crystal symmetry using well‐grown pristine Cr_1+δ_Te_2_ (with δ= 0.612, 0.468, 0.357, and 0.238) on sapphire substrates, excluding the iDMI effect.

To confirm single‐phase Cr_1+δ_Te_2_, we first measured the composition at various sites using top‐view energy‐dispersive spectroscopy (EDS) to ensure the lateral homogeneity of the sample, as represented in Section S1, Supporting Information. In this study, we selected a specific *δ* close to the stable phase based on the formation energy, as detailed in Section S1.1, Supporting Information. Since recent reports have shown that mimic‐THE can arise from two AHE domains or magnetic defects,^[^ ^21^, ^22^, ^35^, ^36^ ^]^ it is important to investigate genuine THE resulting from true chiral spin textures. In this study, we fabricated Cr_1+δ_Te_2_ with a specific thickness of 7 ≈ 8 nm that avoids 1) a too‐thick regime to prevent mimic‐THE arising from magnetic inhomogeneity and 2) an ultrathin regime (<5 nm) to minimize the impact of strain due to lattice mismatch with the substrate. Further details regarding sample characterization are provided in Section S1, Supporting Information. *δ*‐dependent THE was investigated in the common structure, as shown in Figure 1a (top panel). To prevent oxidation of the Cr_1+δ_Te_2_ layer, a 2 nm AlO_*x*_ capping layer was deposited using the electron‐beam deposition method. The notation for the AlO_*x*_ layer is omitted for convenience when denoting specific Cr_1+δ_Te_2_ structures. As shown in Figure 1b, all Cr_1+δ_Te_2_ samples with various *δ* used in this work were epitaxially grown, showing (002) family peaks (c‐axis alignment) with clear Laue oscillations. Moreover, the X‐ray diffraction (XRD) peak of Cr_1+δ_Te_2_ shifts to a lower angle as more Cr atoms are intercalated, indicating an increase in interplanar distance, which is consistent with an increase in the c‐axis lattice constant as *δ* increases in Cr_1+δ_Te_2_.^[^ ^28^ ^]^

The sharp interface between the substrate (or AlO_*x*_ capping layer) and the Cr_1.612_Te_2_ 7.6 nm film (a representative sample among Cr_1+δ_Te_2_s) is confirmed by the high‐angle annular dark‐field scanning transmission electron microscopy (HAADF–STEM) image shown in Figure 1c (left panel) and 1d. EDS elemental mapping data, showing the atomic distribution of Al, Cr, and Te, indicate that the Cr_1.612_Te_2_ (AlO_*x*_ layer) is deposited uniformly on the substrate (Cr_1.612_Te_2_ layer) without atomic interdiffusion at the interfaces, as shown in Figure 1c (middle and right panels). The HAADF–STEM image of Cr_1.612_Te_2_ along the [11$\bar{2}$0] direction demonstrates high‐crystalline epitaxial growth of Cr_1.612_Te_2_ on a sapphire substrate, as shown in Figure 1d. The atomic concentration ratio of Cr:Te for Area #1 is obtained from the EDS spectra using the Cr K series and Te L series, as shown in Figure 1e, resulting in a ratio of 44.58:55.42, represented in Figure 1c (right panel). The atomic concentration ratio of Cr to Te (44.58:55.42) corresponds to δ≈ 0.6, which is consistent with the *δ* = 0.612 calculated using top‐view EDS (Figure S2b,d, Supporting Information). Therefore, 7.6 nm of pristine Cr_1.612_Te_2_ was epitaxially grown on a sapphire substrate with vertical and lateral stoichiometric homogeneity.

When measuring the Hall effect of a magnetic material possessing chiral spin textures, the Hall resistivity signal as a function of magnetic field ($\rho_{xy}\left(H\right)$) exhibits not only OHE due to the transverse deflection of electrons by the Lorentz force and AHE due to magnetization but also THE signal generated by $\Omega_{r}$, originating from the chiral spin texture. The phenomenological layout for the Hall effect family is depicted in Figure 1f. For the case of THE, which is analogous to AHE caused by *k*‐space Berry curvature, its own $\rho_{xy}\left(H\right)$ signal is illustrated in the bottom panel of Figure 1f. For typical collinear magnetic materials where only OHE and AHE occur, $\rho_{xy}\left(H\right)$ can be represented as Equation (1), where $R_{0}$ is the ordinary Hall coefficient, $R_{S}$ is the anomalous Hall coefficient, and *M* is the magnetization.

(1) $$\rho_{xy}\left(H\right)=R_{0}H+R_{S}M$$

When a chiral spin texture emerges, the THE resistivity ($\rho_{\text{THE}}$) term is added to $\rho_{xy}\left(H\right)$ in Equation (1), and the peak of $\rho_{\text{THE}}$ (THE amplitude) can be expressed as Equation (2), where *P* is the spin polarization of FM, and $\Omega_{r}$ is the real‐space Berry curvature which acts as a fictitious magnetic field.

(2) $$\rho_{\text{THE}}^{\text{peak}}=PR_{0}\Omega_{r}$$

Therefore, in noncoplanar FM, the larger the $\Omega_{r}$ created by its spin texture, the greater the fictitious magnetic field becomes, resulting in a larger $\rho_{\text{THE}}^{\text{peak}}$.

## 3 The THE in Cr_1+δ_Te_2_ with δ=  0.612, 0.468, 0.357, and 0.238

**Figure 2:**
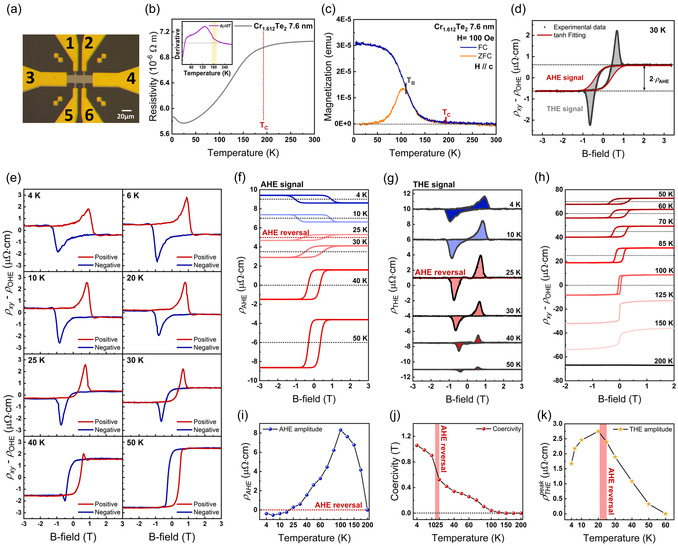
Electrical and magnetic properties of Cr_1.612_Te_2_. a) An optical image of the Hall bar device used in this work. Each Au electrode is numbered from #1 to #6. b) *R*–*T* curve data of Cr_1.612_Te_2_ 7.6 nm. A sharp change in $\rho_{xx}$ is represented by a red‐dotted line. Inset: first‐order derivative of the *R*–*T* curve. The range for the sharp change in the derivative is highlighted by a yellow box. c) SQUID measurement data of Cr_1.612_Te_2_ 7.6 nm. Both FC and ZFC measurements were performed with an applied magnetic field parallel to the c‐axis. d) $\rho_{xy}\left(H\right)$ at 30 K after subtracting $\rho_{\text{OHE}}$ in detail. The $\rho_{\text{AHE}}$ is fitted with a tanh function (red solid line) and $\rho_{\text{THE}}$ is represented by the gray area. e) $\rho_{xy}\left(H\right)$ at different temperatures after subtracting $\rho_{\text{OHE}}$ (4–50 K). Data with increasing (decreasing) field are represented as a red (blue) solid line, denoted as positive (negative). f) AHE contribution of the data in (e) at various temperatures. g) THE contribution of the data in (e) at various temperatures. AHE polarity is reversed near 20–25 K, represented as AHE reversal from now on. h) $\rho_{xy}\left(H\right)$ at different temperatures after subtracting $\rho_{\text{OHE}}$ (>50 K), showing no $\rho_{\text{THE}}$ above 50 K. For $\rho_{\text{AHE}}$ below *T*_B_, $\rho_{\text{AHE}}=0$ is represented by a gray‐dotted line. i) Temperature‐dependent AHE amplitude. $\rho_{\text{AHE}}=0$ is represented by a red‐dotted line. j) The temperature‐dependent coercivity. k) Temperature‐dependent $\rho_{\text{THE}}^{\text{peak}}$. Temperature range for AHE reversal is represented by a red box in (j) and (k).

To investigate the field‐dependent magnetic properties and resistivity as a function of temperature, we used the standard Hall bar device containing six Au electrode pads by patterning all AlO_*x*_/Cr_1+δ_Te_2_/sapphire structures, as shown in **Figure** **2**a. Using the 4‐probe method (as detailed in Experimental Section), the longitudinal resistivity ($\rho_{xx}$) versus temperature (*R*–*T*) curve was obtained for a sample of Cr_1.612_Te_2_ (7.6 nm), which is close to Cr_5_Te_6_ (δ=2/3), showing that the resistivity at 300 K is $\approx 7.05\times 10^{-6}\;\text{Ω}\cdot m$, as shown in Figure 2b. Additionally, a sharp change in $\rho_{xx}$ is observed between 190 and 200 K, which is attributed to the ferromagnetic transition upon reaching the Curie temperature (*T*_C_) of the 2D FM. When ferromagnetic ordering occurs in 2D FM with magnetic anisotropy, it induces the opening of a thermal excitation gap in the magnon density of states, leading to a significant reduction in $\rho_{xx}$ due to decreased electron–magnon scattering.^[^ ^28^, ^37^, ^38^ ^]^ Therefore, identifying the point of sharp resistance change in the *R*–*T* curve of 2D FM allows for the indirect determination of its *T*_C_. To further investigate the $\rho_{xx}$ change in detail, a first‐order derivative graph of the *R*–*T* curve is shown in the inset of Figure 2b: a sharp change in the derivative value is observed around 190–200 K, which is consistent with Figure 2b. The conductivity curves of all Cr_1+δ_Te_2_ used in this work are depicted in Section S1.6, Supporting Information, where the change in conductivity is clearly observed. To investigate the magnetic properties of 7.6 nm thick Cr_1.612_Te_2_ in detail, we measured the *M* versus temperature (*M*–*T*) curve under a magnetic field of 100 Oe parallel to the c‐axis during both zero‐field‐cooling (ZFC) and field‐cooling (FC) processes using a superconducting quantum interference device (SQUID), as shown in Figure 2c. The FC curve demonstrates a gradual decrease in magnetization with increasing temperature, confirming the *T*_C_ of Cr_1.612_Te_2_, ≈195 K. In addition, the blocking temperature (*T*_B_) can be determined through ZFC measurements, yielding a *T*_B_ of ≈108 K for Cr_1.612_Te_2_. Comparing the *T*_C_ obtained indirectly from the *R*–*T* curve (Figure 2b) with the SQUID measurement (Figure 2c) yielded consistent results, showing that the method used to determine *T*_C_ in Figure 2b is very effective.

To investigate THE and AHE contributions in Cr_1.612_Te_2_, we measured $\rho_{xy}\left(H\right)$ with varying temperatures below the measured *T*_C_, as shown in Figure 2e,h. The Hall resistivity signals in Figure 2e,h were plotted after subtracting the linear term at high fields, corresponding to the OHE term ($\rho_{\text{OHE}})$. In particular, as shown in Figure 2e, a distinct hump‐shaped signal in the temperature‐dependent $\rho_{xy}-\rho_{\text{OHE}}$ is clearly visible, along with the contribution of the hysteresis loop due to AHE. To separate the AHE signal ($\rho_{\text{AHE}})$ from $\rho_{xy}-\rho_{\text{OHE}}$, $\rho_{\text{AHE}}$ was fitted out using the quantum mechanical approach of the $M_{s}\;\text{tanh}\left(\frac{H}{a}-H_{c0}\right)$ function, where $M_{s},\;a,$ and$\;H_{c0}$ are fitting parameters, instead of the Langevin function (semiclassical approach).^[^ ^9^, ^19^ ^]^ The remaining fitting residue was then obtained to yield the THE signal ($\rho_{\text{THE}}$). Other reports have used the method of subtracting the negative sweep from the positive sweep to isolate $\rho_{\text{THE}}$ from $\rho_{xy}-\rho_{\text{OHE}}$.^[^ ^39^, ^40^ ^]^ However, considering the behavior of noncoplanar spin texture with respect to the magnetic field, this fitting method can introduce significant inaccuracies. For instance, skyrmions are formed during the process of domain switching (magnetization reversal), rather than being formed after the spins of the FM are fully aligned by the magnetic field. Therefore, Figure 2d, presented as representative data of $\rho_{xy}-\rho_{\text{OHE}}$ at 30 K, shows how the AHE and THE signals were separated in this study, considering that $\rho_{\text{THE}}$ cannot appear after AHE resistivity is saturated. The fitting was conducted by applying constraints; coercivity, defined as $a\;\cdot \;H_{c0}$, to be near the field at which $\rho_{\text{THE}}$ reaches its peak value while minimizing the fitting errors. Details of the fitting process, including the issues arising from improper fitting, are described in Section S2.1, Supporting Information. As the temperature approaches 50 K, $\rho_{\text{THE}}$ becomes nearly negligible in $\rho_{xy}-\rho_{\text{OHE}}$, leaving only the contribution from $\rho_{\text{AH}}$. $\rho_{\text{AHE}}$ obtained from fitting in the range of 4–50 K and the temperature‐dependent magnitude of $\rho_{\text{AHE}}$ (AHE amplitude) are represented in Figure 2f,i, respectively, where fitting details for each temperature are provided in Section S2.2, Supporting Information. Finally, the residue obtained by subtracting $\rho_{\text{AHE}}$ from $\rho_{xy}-\rho_{\text{OHE}}$ for each temperature yielded $\rho_{\text{THE}}$, as presented in Figure 2g. Moreover, the peak value of $\rho_{\text{THE}}$ ($\rho_{\text{THE}}^{\text{peak}}$) as a function of temperature using Figure 2g is plotted in Figure 2k. The maximum $\rho_{\text{THE}}^{\text{peak}}$ of ≈2.75 μΩ⋅cm at 20 K was observed in Cr_1.612_Te_2_, which can be attributed to the bulk inversion symmetry breaking induced $\Omega_{r}$ stabilized by the bulk DMI.

In Figure 2e,f, an unusual phenomenon is observed in Cr_1.612_Te_2_, where the polarity of AHE varies with temperature in the vicinity of 20–25 K. This change in AHE polarity, referred to as AHE reversal, occurs uniquely in several Cr_1+δ_Te_2_ with different *δ*,^[^ ^19^, ^36^, ^40^, ^41^ ^]^ attributed to the delicate variation of *k*‐space Berry curvature of Cr_1+δ_Te_2_ near the Fermi level, as shown in theoretical calculation results.^[^ ^36^, ^42^ ^]^ $\rho_{xy}-\rho_{\text{OHE}}$ (>50 K) data in Figure 2h confirm the absence of $\rho_{\text{THE}}$, i.e., $\rho_{xy}-\rho_{\text{OHE}}=\rho_{\text{AHE}}$. AHE amplitudes for >50 K, plotted in Figure 2i, show that the AHE amplitude increases up to 100 K and then gradually decreases until approaching *T*_C_, due to the decrease in *M* with increasing temperature. The coercivity values obtained from $\rho_{\text{AHE}}$ (4–200 K) for Cr_1.612_Te_2_ in Figure 2j show a typical decreasing trend with increasing temperature, as observed in typical FM, with a sharp drop to zero at temperatures higher than *T*_B_ (≈108 K).

**Figure 3:**
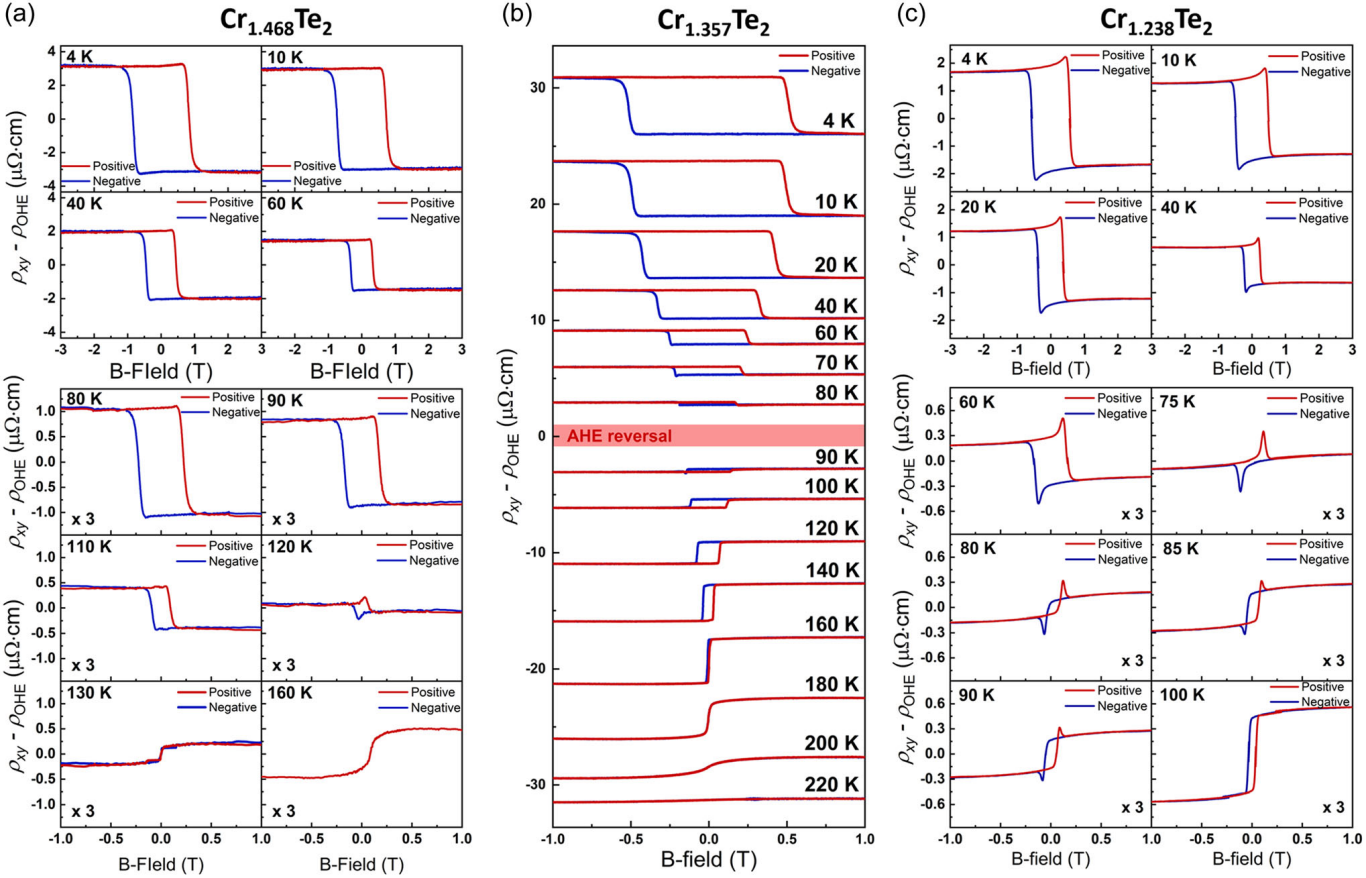
Hall resistivity as a function of the B‐field at various temperatures for different *δ*. $\rho_{xy}-\rho_{\text{OHE}}$ as a function of the B‐field with different temperatures for a) Cr_1.468_Te_2_ 7.7 nm (4–160 K), b) Cr_1.357_Te_2_ 7.4 nm (4–220 K), and c) Cr_1.238_Te_2_ 8 nm (4–100 K). The temperature range for AHE reversal is indicated by the red box in (b). All Cr_1+δ_Te_2_ with δ= 0.468, 0.357, and 0.238 exhibit AHE reversal at different temperatures.

So far, few reports have focused on the chiral spin texture or THE observed at a specific single δ  in pristine Cr_1+δ_Te_2_. However, no studies have specifically explored how the THE properties, which are directly linked to $\Omega_{r}$, of pristine Cr_1+δ_Te_2_ are affected by the amount of Cr intercalant and the underlying causes of the effect. Notably, since Cr self‐intercalation is a straightforward method for tuning the crystal symmetry of Cr_1+δ_Te_2_, it is a reasonable approach for controlling the DMI energy or $\Omega_{r}$ in Cr_1+δ_Te_2_. Considering that $\Omega_{r}$, generated by chiral spin textures, is the origin of THE in pristine magnetic films, bulk DMI, which plays a crucial role in stabilizing chiral spin textures, is essential for understanding THE. Therefore, to investigate the mechanism and the role of bulk DMI in the THE of Cr_1+δ_Te_2_, we analyzed the $\rho_{xy}-\rho_{\text{OHE}}$ characteristics of Cr_1+δ_Te_2_ depending on gradually varying *δ*. As shown in **Figure** **3**, $\rho_{xy}-\rho_{\text{OHE}}$ was investigated for δ= 0.468, 0.357, and 0.238 by reducing Cr intercalant from Cr_1.612_Te_2_ (δ= 0.612). All of the Cr_1+δ_Te_2_ samples used in this work exhibit lateral homogeneity, as demonstrated in Section S1, Supporting Information. To prevent the influence of thickness on THE, all samples were set to have a thickness ranging from 7 to 8 nm, including Cr_1.612_Te_2_.

Cr_1.468_Te_2_, which is close to Cr_3_Te_4_ with 50% (δ=1/2) of the intercalated Cr atoms in the vdW gap, shows a weak THE signal below 120 K, as shown in the temperature‐dependent $\rho_{y}-\rho_{\text{OHE}}$ curve in Figure 3a. However, the AHE amplitude at low temperatures is significantly larger than that of Cr_1.612_Te_2_, resulting in a relatively small THE signal. Upon fitting the $\rho_{xy}-\rho_{\text{OHE}}$ curve using the tanh function, the maximum $\rho_{\text{THE}}^{\text{peak}}$ value of Cr_1.468_Te_2_ is notable (1.12  μΩ⋅cm at 4 K), although it is ≈2.5 times smaller compared to that of Cr_1.612_Te_2_ (2.75 μΩ⋅cm at 20 K). Therefore, Cr_1.468_Te_2_ is expected to have a non‐centrosymmetric crystal symmetry, leading to THE mediated by ${\text{Ω}}_{r}$ originating from bulk DMI.

Cr_1.357_Te_2_, which is close to Cr_2_Te_3_ (33.3% [δ=1/3]) with a further reduction of intercalated Cr atoms in the vdW gap, exhibits negligible THE contribution, as shown in the temperature‐dependent $\rho_{xy}-\rho_{\text{OHE}}$ curve in Figure 3b. If THE in Cr_1+δ_Te_2_ is dominated by bulk DMI, THE is not expected to emerge near the composition of Cr_2_Te_3_ because Cr_2_Te_3_ has a centrosymmetric crystal structure (P$\bar{3}$1c), which aligns with our experimental results in Figure 3b. In addition, Cr_1.238_Te_2_, where the Cr atom is slightly less intercalated compared to Cr_5_Te_8_ (Cr atoms are 25% intercalated [δ=1/4] into the vdW gap), shows considerable contributions of THE at temperatures below 90 K, as shown in the $\rho_{xy}-\rho_{\text{OHE}}$ curve in Figure 3c. This is consistent with previous reports demonstrating that Cr_1+δ_Te_2_ with δ≈ 0.3 exhibits non‐centrosymmetric crystal symmetry (P3m1).^[^ ^17^ ^]^ Moreover, the atomic positions of the space group P3m1 along the c‐axis exhibit inversion asymmetry, and P3m1 is deformed from a (2 × 2) supercell of CrTe_2_ (centrosymmetric P$\bar{3}$m1), not P$\bar{3}$1c, indicating that effective crystal symmetry tuning is possible through Cr intercalation. Therefore, our experimental results for various *δ* provide evidence that THE in Cr_1+δ_Te_2_ is due to bulk DMI, highlighting the significant role of crystal symmetry in engineering $\Omega_{r}$ (THE). The results of overall AHE amplitude and coercivity for Cr_1+δ_Te_2_ with different *δ*, as investigated in Figure 2 and 3, are summarized in Figure S17, Supporting Information (Section S2.6, Supporting Information).

## 4 The Bulk DMI Strength‐Dependent THE in Cr_1+δ_Te_2_

**Figure 4:**
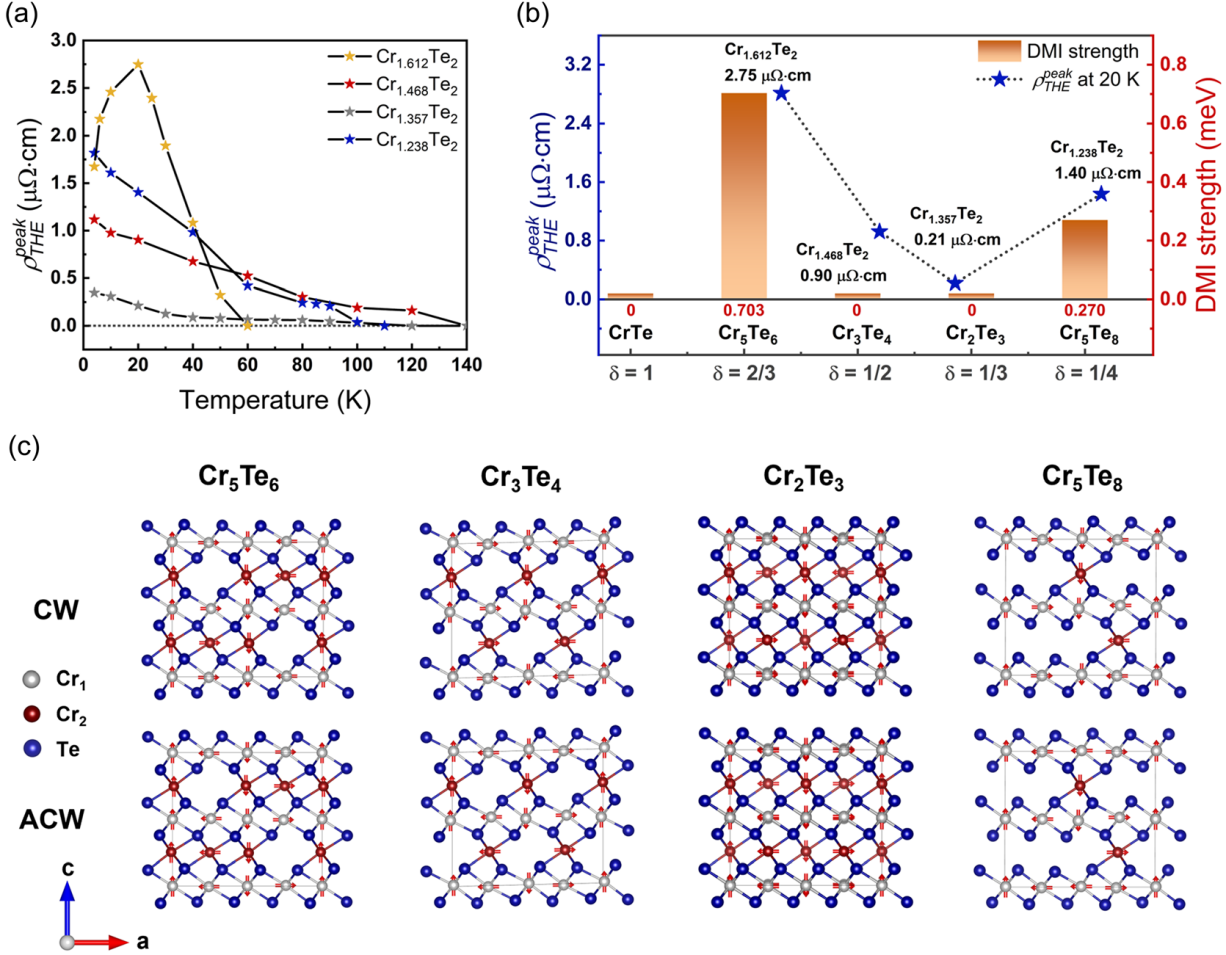
First‐principles calculations for bulk DMI strength. a) Temperature‐dependent $\rho_{\text{THE}}^{\text{peak}}$ values. b) The calculated bulk DMI strength of Cr_1+δ_Te_2_ (with δ= 1, 2/3, 1/2, 1/3, and 1/4) and experimentally measured $\rho_{\text{THE}}^{\text{peak}}$ of Cr_1+δ_Te_2_ (δ = 0.612, 0.468, 0.357, and 0.238) at 20 K. c) Magnetic unit cell of Cr_1+δ_Te_2_ (with δ= 2/3, 1/2, 1/3, and 1/4) with CW and ACW spin configurations.

For each Cr_1+δ_Te_2_ with a different *δ*, we subtracted the AHE contribution using the tanh function for different temperatures and obtained the peak corresponding to the temperature‐dependent $\rho_{\text{THE}}^{\text{peak}}$, as shown in **Figure** **4**a. Fitting details of the AHE contribution and THE contribution for each *δ* at different temperatures can be found in Section S2.2 through S2.5, Supporting Information. As shown in Figure 4a, $\rho_{\text{THE}}^{\text{peak}}$ of Cr_1+δ_Te_2_ with δ = 0.468, 0.357, and 0.238 shows a monotonic change as a function of temperature, while that of Cr_1.612_Te_2_ shows a non‐monotonic change as a function of temperature. To verify whether the observed giant THE in pristine Cr_1.612_Te_2_ and Cr_1.238_Te_2_ is genuine, where the effects of two magnetic domains and iDMI were excluded, first, we attempted to fit the temperature‐dependent coercivity, $H_{C}\left(T\right)$, using Kneller's law, $H_{C}\left(T\right)=H_{0}\;\cdot \;\left[1-{\left(\frac{T}{T_{B}}\right)}^{\alpha }\right]$. If the fitting parameter, α, gives an optimum fit at 1/2, this indicates that the material is a single magnetic domain.^[^ ^43^ ^]^ As shown in Section S3.2 and S3.3, Supporting Information, we confirmed that all Cr_1+δ_Te_2_ samples used in this study exhibit single magnetic domain. Second, we fitted the $\rho_{xy}-\rho_{\text{OHE}}$ curve using the two‐AHE model (Section S3.3 and S3.4, Supporting Information). We were unable to obtain a complete fit due to physical errors, suggesting that the measured data is indeed due to THE caused by chiral spin textures, not two‐AHE domains. Therefore, Cr_1.612_Te_2_ and Cr_1.238_Te_2_ exhibit a genuine THE originating from chiral spin textures. To investigate the origin of colossal THE in Cr_1.612_Te_2_, we conducted B‐field angle‐dependent THE measurements, as shown in Figure S27, Supporting Information (Section S4, Supporting Information). Unlike THE previously reported in skyrmions, the THE contribution in Cr_1.612_Te_2_ is unique in that it remains even when the B‐field is tilted up to ≈60° with respect to the c‐axis, indicating that Cr_1.612_Te_2_ possesses a noncoplanar spin texture.^[^ ^11^, ^40^ ^]^ This finding is consistent with previous reports that skyrmions have not been observed in Cr_1+δ_Te_2_ with δ≈0.6,^[^ ^15^ ^]^ but rather a noncoplanar spin texture is observed with δ≈2/3.^[^ ^40^ ^]^ Therefore, these results suggest that THE in Cr_1.612_Te_2_ is due to $\Omega_{r}$ induced by the noncoplanar spin texture stabilized by bulk DMI.

To further verify whether the THE observed in Cr_1+δ_Te_2_ is primarily governed by the bulk DMI strength (*E*_DMI_) arising from bulk crystal symmetry breaking, we performed DFT calculations to evaluate the *E*_DMI_ across various phases of Cr_1+δ_Te_2_ with δ= 1, 2/3, 1/2, 1/3, and 1/4. As shown in Figure 4c, each Cr_1+δ_Te_2_ composition was modeled within a (2 × 1 × 1) supercell structure, with an exception for δ= 1/3 within a (2 × 3 × 1) supercell, allowing us to simulate clockwise (CW) and anticlockwise (ACW) chiral spin orientations within the magnetic unit cell. By subtracting the energy for the CW spin configuration from that for the ACW spin configuration, *E*_DMI_ was estimated using Equation (3), where the constant $8\sqrt{3}$ is derived from the Moriya symmetry rule.^[^ ^40^ ^]^

(3) $$E_{\text{DMI}}=(E_{\text{ACW}}-E_{\text{CW}})/8\sqrt{3}$$

As shown in Figure 4b, we observed notable values of *E*_DMI_ for Cr_5_Te_6_ (δ=2/3) and Cr_5_Te_8_ (δ=1/4), which were 0.703 and 0.270 meV, respectively. In contrast, other values of *δ* (1, 1/2, and 1/3) yielded an *E*_DMI_ to nearly zero, indicating nonlinear bulk inversion symmetry breaking with respect to *δ*. This bulk DMI strength directly influences the formation of chiral spin textures in the magnetic thin films, such that samples with *δ* close to 2/3 and 1/4, corresponding to Cr_1.667_Te_2_ and Cr_1.25_Te_2_, exhibit noncollinear spin configurations. Remarkably, this result is well consistent with our experimental data, which reveals a pronounced THE in Cr_1.612_Te_2_ and Cr_1.238_Te_2_, as shown in Figure 2 and 3, providing strong evidence of DMI‐driven chiral behavior.

Given that all Cr_1+δ_Te_2_ samples show a monotonic $\rho_{\text{THE}}^{\text{peak}}$ with respect to temperature above 20 K, we selected this as the minimum temperature for assessing the impact of *E*_DMI_ on the THE signal. This is evident in Figure 4b, where the $\rho_{\text{THE}}^{\text{peak}}$ of Cr_1+δ_Te_2_ (δ  = 0.612, 0.468, 0.357, and 0.238) at 20 K correlates strongly with *E*_DMI_. As *δ* varies, $\rho_{\text{THE}}^{\text{peak}}$ shows a direct proportionality to *E*_DMI_, with the highest values observed near δ≈0.6, underscoring the critical role of DMI in tuning chiral spin textures. Furthermore, the zero *E*_DMI_ value of Cr_2_Te_3_ (Cr_1+1/3_Te_2_) aligns with the negligible THE observed in Cr_1.357_Te_2_, indicating that Cr_1+δ_Te_2_ with δ≈1/3 possesses an inversion‐symmetric crystal structure, as theoretically expected. Interestingly, while the calculated *E*_DMI_ for Cr_3_Te_4_ (≈0) differs from the observed minor THE in Cr_1.468_Te_2_ (our study) and the chiral spin texture in Cr_3_Te_4_,^[^ ^29^ ^]^ this deviation is likely due to local inversion symmetry breaking. Although some discrepancies exist between our theoretical evaluation and experimental results for the Cr_3_Te_4_ sample, our findings collectively suggest that the THE strength in pristine Cr_1+δ_Te_2_ is fundamentally driven by a crystal symmetry‐dependent bulk DMI strength induced by Cr intercalation.

**Figure 5:**
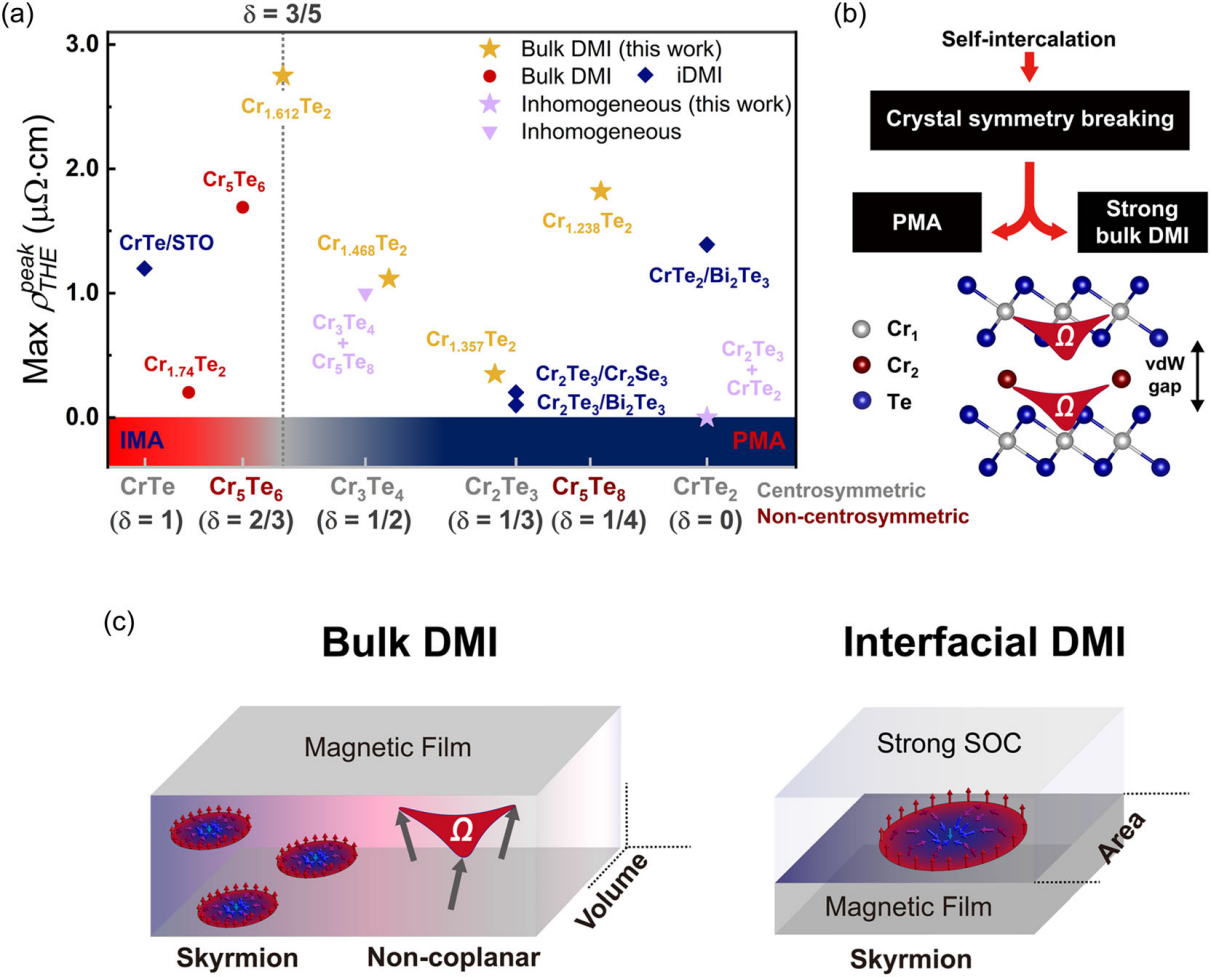
Bulk DMI‐induced colossal THE in pristine Cr_1+δ_Te_2_. a) Summary of the maximum $\rho_{\text{THE}}^{\text{peak}}$ values for the Cr_1+δ_Te_2_ family. The maximum $\rho_{\text{THE}}^{\text{peak}}$ values were extracted from CrTe_2_/Bi_2_Te_3_,^[^ ^19^ ^]^ Cr_2_Te_3_/Bi_2_Te_3_,^[^ ^20^ ^]^ Cr_0.87_Te,^[^ ^31^ ^]^ Cr_5_Te_6_,^[^ ^40^ ^]^ Cr_3_Te_4_ + Cr_5_Te_8_,^[^ ^41^ ^]^ Cr_2_Se_3_/Cr_2_Te_3_,^[^ ^42^ ^]^ and CrTe/SrTiO_3_.^[^ ^47^ ^]^ The region where the magnetic anisotropy of Cr_1+δ_Te_2_ changes, approximately at δ≈0.6, is indicated by a gray‐dotted line. b) Strategy for designing $\Omega_{r}$ in 2D van der Waals FM. c) Schematic illustration of chiral spin textures inducing $\Omega_{r}$ stabilized by bulk DMI and iDMI, respectively.

Although there have been attempts to control $\Omega_{r}$ through mechanisms such as iDMI, the maximum $\rho_{\text{THE}}^{\text{peak}}$ of Cr_1.612_Te_2_ (≈2.75 μΩ⋅cm at 20 K) is the highest among $\rho_{\text{THE}}^{\text{peak}}$ values reported in the Cr_1+δ_Te_2_ family to date. This value surpasses the previously known maximum $\rho_{\text{THE}}^{\text{peak}}$ value for bulk DMI‐mediated THE in Cr_5_Te_6_ (1.6 μΩ⋅cm at 90 K)^[^ ^40^ ^]^ and even exceeds the value observed in CrTe_2_/Bi_2_Te_3_ stack (1.39 μΩ⋅cm at 10 K)^[^ ^19^ ^]^ utilizing iDMI. To explore efficient strategies for controlling $\Omega_{r}$, which is proportional to $\rho_{\text{THE}}^{\text{peak}}$, in vdW FMs such as Cr_1+δ_Te_2_, we summarized the reported $\rho_{\text{THE}}^{\text{peak}}$ values of the Cr_1+δ_Te_2_ family according to their physical origins in **Figure** **5**a. Bulk DMI and iDMI are the representative physical mechanisms that give rise to nontrivial $\Omega_{r}$ in magnetic films, as shown in Figure 5c. Specifically, breaking the crystal inversion symmetry of the magnetic film induces bulk DMI, while constructing heterostructures with materials possessing strong spin–orbit coupling facilitates iDMI. However, the two mechanisms differ fundamentally: bulk DMI generates volumetric chiral spin textures, whereas iDMI confines chiral spin textures to the interface area. As a result, controlling bulk DMI is expected to be a more efficient approach for designing high‐density and high‐performance chiral spintronic devices. This is further corroborated by Figure 5a and **Table** **1**, which show that bulk DMI, induced by self‐intercalation‐driven bulk inversion symmetry breaking, produces significantly higher $\rho_{\text{THE}}^{\text{peak}}$ than those generated by iDMI, highlighting the superior effectiveness of bulk DMI in controlling $\Omega_{r}$ in magnetic films. Moreover, many reports have calculated iDMI‐induced THE resistivity by multiplying the entire film thickness factor, despite $\Omega_{r}$ being confined only to the interface area, leading to an overestimation of $\rho_{\text{THE}}^{\text{peak}}$ ($\Omega_{r})$.

**Table 1:** Summary of maximum THE amplitude, temperature, and origin of THE in the Cr_1+δ_Te_2_ family.

Sample	Maximum THE amplitude [μΩ⋅cm]	Maximum THE temperature [K]	Origin
Cr_1.612_Te_2_ (this work)	2.75	20	Bulk DMI
Cr_1.468_Te_2_ (this work)	1.12	4	Local symmetry breaking
Cr_1.357_Te_2_ (this work)	0.35	4	Bulk DMI
Cr_1.238_Te_2_ (this work)	1.82	4	Bulk DMI
Cr_2_Te_3_ + CrTe_2_ (this work)	None	None	Inhomogeneity
Cr_5_Te_6_^[^ ^40^ ^]^	1.6	90	Bulk DMI
Cr_0.87_Te^[^ ^31^ ^]^	0.74	10	Bulk DMI
CrTe_2_/Bi_2_Te_3_^[^ ^19^ ^]^	1.39	10	Interfacial DMI
CrTe/SrTiO_3_^[^ ^47^ ^]^	1.2	55	Interfacial DMI
Cr_2_Se_3_/Cr_2_Te_3_^[^ ^42^ ^]^	0.2	10	Interfacial DMI
Cr_2_Te_3_/Bi_2_Te_3_^[^ ^20^ ^]^	0.1	25	Interfacial DMI
Cr_3_Te_4_ + Cr_5_Te_8_^[^ ^41^ ^]^	1	50	Inhomogeneity

In addition to bulk DMI and iDMI, THE has also been observed in inhomogeneous thin films, such as (Cr_3_Te_4_ + Cr_5_Te_8_, 1 μΩ⋅cm at 50 K).^[^ ^41^ ^]^ However, in our CrTe_2_ + Cr_2_Te_3_ inhomogeneous thin film (Section S6, Supporting Information), negligible THE was observed. This suggests that THE in inhomogeneous thin films is governed by the crystal symmetry and *E*_DMI_ within the specific composition of the film. Thus, lateral or vertical inversion symmetry breaking through inhomogeneity is not an effective method for controlling $\Omega_{r}$.

Since the bulk DMI strength for Cr_1.612_Te_2_ cannot exhibit non‐monotonic behavior as a function of temperature, another factor related to the magnetic anisotropy is involved in non‐monotonic temperature dependence of $\rho_{\text{THE}}^{\text{peak}}$, as shown in Figure 4a. As *δ* approaches 1 in Cr_1+δ_Te_2_, the transition from 2D vdW FM to 3D FM occurs, accompanied by the onset of in‐plane magnetic anisotropy (IMA).^[^ ^15^, ^40^, ^44^, ^45^, ^46^ ^]^ The onset of IMA occurs when δ>0.6,^[^ ^15^ ^]^ resulting in decrease of THE amplitude, as detailed in Section S5, Supporting Information, which is consistent with the decreased $\rho_{\text{THE}}^{\text{peak}}$ in Cr_5_Te_6_^[^ ^40^ ^]^ compared to that of Cr_1.612_Te_2_. The *M*–*T* curve of Cr_1.612_Te_2_ measured under a magnetic field along the in‐plane direction shows slight magnetization compared to the out‐of‐plane magnetization, as provided in Figure S28, Supporting Information (Section S5, Supporting Information). Consequently, the contribution of in‐plane magnetization increases as the temperature decreases: i.e., relatively weakened perpendicular magnetic anisotropy (PMA) affects the spin texture, leading to a decrease in $\rho_{\text{THE}}^{\text{peak}}$, as described in detail in Section S5, Supporting Information. Similarly, Cr_5_Te_6_ exhibits non‐monotonic $\rho_{\text{THE}}^{\text{peak}}$ due to the manifestation of antiferromagnetic ordering at a certain temperature.^[^ ^40^ ^]^ Materials such as CrTe/SrTiO_3_,^[^ ^47^ ^]^ EuIn_2_As_2_,^[^ ^48^ ^]^ and SrRuO_3_/SrTiO_3_^[^ ^49^ ^]^ would be other examples showing non‐monotonic $\rho_{\text{THE}}^{\text{peak}}$ as a function of temperature. Unlike Cr_1.612_Te_2_, the $\rho_{\text{THE}}^{\text{peak}}$ of other Cr_1+δ_Te_2_s shows monotonic behavior with respect to temperature, demonstrating the strong PMA of Cr_1+δ_Te_2_ with δ<0.6.

Therefore, the $\rho_{\text{THE}}^{\text{peak}}$ in Cr_1+δ_Te_2_ is expected to exhibit a maximum value near δ≈0.6, owing to the onset of IMA for δ>0.6 ^[^ ^15^ ^]^ and considering the maximum *E*_DMI_ near δ≈0.66. This is consistent with our study recording the largest value of 2.75 μΩ⋅cm in Cr_1.612_Te_2_. Thus, effective control of $\Omega_{r}$ in 2D FMs can be achieved through the strategy illustrated in Figure 5b: first, confirming whether self‐intercalation can break inversion symmetry. Then, by considering the magnetic anisotropy and *E*_DMI_ induced by self‐intercalation, adjusting the self‐intercalant to satisfy PMA and strong *E*_DMI_, as shown in Figure 5b. This strategy is highly promising for controlling $\Omega_{r}$ in other self‐intercalatable 2D FMs, such as VTe_*x*_ and MnTe_*x*_.

## 5 Conclusion

In summary, we have successfully fabricated Cr_1+δ_Te_2_ thin films with stoichiometric and magnetic homogeneity using MBE. By systematically adjusting the amount of Cr intercalant, we identified that the THE induced by real‐space Berry curvature can be nonlinearly modulated as a function of *δ*, in relation to the crystal symmetry breaking. We demonstrated that both the bulk DMI strength and crystal symmetry in Cr_1+δ_Te_2_ can be tuned depending on *δ*, showing that the amplitude of THE strongly depends on the bulk DMI strength, as supported by DFT calculations. Particularly, a giant THE amplitude of 2.75 μΩ⋅cm was observed in Cr_1.612_Te_2_, which is not only the highest value reported among the Cr_1+δ_Te_2_ family to date but also a candidate for the maximum achievable value. Furthermore, our work underscores the critical role of bulk DMI in engineering real‐space Berry curvature, highlighting its superiority over iDMI for developing high‐efficiency and high‐density chiral spintronic devices. Our systematic study not only provides a fundamental understanding of THE in Cr_1+δ_Te_2_ but also proposes a robust strategy for designing highly efficient chiral spintronic devices through the straightforward method of self‐intercalation.

## 6 Experimental Section

6.1

6.1.1

##### Sample Characterization

Cr_1+δ_Te_2_ with various *δ* used in this work was fabricated on double‐side‐polished c‐plane sapphire (0001) 750 μm substrate using MBE equipped with Knudsen cells at a working pressure of 3 × 10^−9^ Torr. Sapphire substrates were annealed at 300 °C for 30 min and at 600 °C for 1 h to degas out the impurities from the surface of the sapphire before deposition. To achieve the target Cr_1+δ_Te_2_ with a specific *δ*, sapphire substrates were maintained at temperatures ranging from 200 to 360 °C depending on *δ* during the deposition of Cr_1+δ_Te_2_ films, and the temperature of the Knudsen cell used for evaporating Cr was delicately controlled (1280–1325 °C) to regulate the flux of Cr evaporation. The ratio of Cr flux to Te flux was adjusted to ≈1:6.03–1:2.71 depending on *δ* to grow each targeted Cr_1+δ_Te_2_ film. Further details on sample fabrication are described in Section S1.1, Supporting Information. AlO_*x*_ 2 nm cappings were deposited on all samples using the electron‐beam evaporation method in a vacuum (<10^−6^ torr), and all processes were performed in situ without air exposure.

To obtain XRD spectra to characterize the crystal orientation, we used an XRD instrument (Rigaku Smartlab). The composition of each Cr_1+δ_Te_2_ was confirmed via field‐emission scanning electron microscope (JEOL‐7800F and JSM‐IT‐500HR) under 15 kV accelerating voltage. To characterize the surface morphology and roughness of samples, we used atomic force microscopy (Park system Nx‐10). SQUID‐vibration sample magnetometer (SQUID‐VSM, Quantum Design MPMS3) with magnetization sensitivity of 10^−9^ emu was used to obtain *M*–*T* curves. The composition of Cr_1.612_Te_2_ was reconfirmed using in situ X‐ray photoelectron spectroscopy (Ulvac PHI 5000 VersaProbe) as shown in Figure S3, Supporting Information.

##### Device Fabrication and Magneto‐Transport Measurement

A standard Hall bar device was fabricated using the photolithography method. Using a positive photoresist (AZ GXR 601) and mask aligner (Suss MicroTec MA6), the transport channel of both the width and length of 20 μm was patterned. For measuring Hall resistivity, the width of the transverse bar was patterned to 5 μm. To etch the samples, an inductively coupled plasma reactive ion etching system (LAT) was utilized. Au electrodes were deposited to a thickness of 80 nm using the electron‐beam evaporation method in vacuum (<10^−6^ torr).

For the measurement of magneto‐transport properties, we employed a physical property measurement system (Cryogenic mCFM9T). As shown in the electrode connections of Figure 2a, for measuring longitudinal resistivity ($\rho_{xx}$) and Hall resistivity ($\rho_{xy}$) using the 4‐probe method, an electric current of 10^−5^ *A* was applied through electrode #3 (source) to #4 (ground). Then, $\rho_{xx}$ ($\rho_{xy}$) was calculated by measuring the voltage difference, $\Delta V_{xx}$ ($\Delta V_{xy}$), between electrodes #1 (#1) and #2 (#5). The Hall bar devices used in this study were configured so that the width and length of the transport channel were the same as 20 μm. Therefore, $\rho_{xx}$ ($\rho_{xy}$) can be calculated by $\rho_{xx}\;\left(\rho_{xy}\right)=\frac{\Delta V_{xx}\;\left(\Delta V_{xy}\right)}{10^{-5}\;A}\cdot t$, where *t* is the thickness of Cr_1+δ_Te_2_. An electric current of 10^−5^ *A* was applied through electrodes using a source meter (Keithley 2400). Longitudinal and transverse resistivity were measured using nanovoltmeters (Keithley 2182A and Keysight 34420A, respectively). Magnetic fields ranging from −3 to 3 T were applied to the samples, with a magnetic field sweep interval set to ≈0.0005 T (5 Oe) in both positive and negative sweeps. Therefore, for each temperature, the $\rho_{xy}\left(H\right)$ curve was plotted using over 10 000 data points.

##### Transmission Electron Microscopy

Samples for cross‐sectional HAADF–STEM were fabricated using a focused ion beam (FIB) system (HITACH Ethos NX5000). To reduce the damage caused by Ga ions to the sample, various voltages ranging from 30 to 3 kV were used to fabricate FIB samples. To acquire HAADF–STEM images and EDS elemental mapping, FEI Double Cs‐corrected Titan Themis transmission electron microscope instrument equipped with Super‐X energy dispersive X‐ray (EDX) detector with an X‐FEG module was operated at 300 kV.

##### DFT Calculations

The first‐principles calculations^[^ ^50^ ^]^ were carried out to get DMI strength^[^ ^51^ ^]^ by using the Vienna Ab initio Simulation Package (VASP).^[^ ^52^ ^]^ The plane wave basis energy cutoff was set to 450 eV. The projector‐augmented wave pseudopotentials were used and the generalized gradient approximation of Perdew–Burke–Ernzerhof was considered for exchange‐correlation functionals. The Cr_1+δ_Te_2_ structures were relaxed until the force applied to every atom was smaller than 0.01 eV Å^−1^. The electronic self‐consistent loop was relaxed until the total energy reached 10^−6 ^eV. The Γ‐centered 5 × 20 × 5 *k* (3 × 3 × 3 *k*) mesh was used to sample the Brillouin zone of (2 × 1 × 1) supercell ((2 × 3 × 1) supercell).

## Conflict of Interest

The authors declare no conflict of interest.

## Author Contributions

**Seungwon Rho**: conceptualization (lead); data curation (lead); formal analysis (lead); investigation (lead); methodology (lead); visualization (lead); writing—original draft (lead). **Dameul Jeong**: data curation (equal); methodology (equal); validation (lead); writing—review & editing (supporting). **Hyeong‐Ryul Kim**: data curation (equal); methodology (equal); validation (lead); writing—review & editing (supporting). **Jaeseok Huh**: conceptualization (supporting); data curation (equal); investigation (supporting). **Hyeong‐Jun Son**: data curation (supporting); formal analysis (supporting); investigation (supporting). **Young‐Kyun Kwon**: formal analysis (equal); methodology (equal); supervision (equal); validation (lead); writing—review & editing (lead). **Mann‐Ho Cho**: formal analysis (equal); funding acquisition (lead); project administration (lead); supervision (lead); writing—review & editing (lead).

## Supporting information

Supplementary Material

## Data Availability

The data that support the findings of this study are available from the corresponding author upon reasonable request.
