# Supplementary material for: Designing Nontrivial Real‐Space Berry Curvature through Non‐Monotonic Bulk Inversion Symmetry Breaking in Self‐Intercalated Cr1+δTe2
Source: Small Sci. 2025 Mar 24;5(6):2500028. doi: 10.1002/smsc.202500028 (PMC12168622; doi:10.1002/smsc.202500028)
Supplement: Supplementary file 1 — Supplementary Material [file SMSC-5-2500028-s001.pdf]

Supporting Information for

Designing Non-Trivial Real Space Berry Curvature through Non-Monotonic Bulk Inversion Symmetry Breaking in Self-Intercalated $Cr_{1+\delta}Te_2$

*Seungwon Rho[1], Dameul Jeong[3], Hyeong-Ryul Kim[3], Jaeseok Huh[1], Hyeong-Jun Son[1], Young-Kyun Kwon[3,4], and Mann-Ho Cho[1,2]**

[1]Department of Physics, Yonsei University, Seoul 03722, Republic of Korea

[2]Department of System Semiconductor Engineering, Yonsei University, Seoul 03722, Republic of Korea

[3]Department of Physics and Research Institute for Basic Sciences, Kyung Hee University, Seoul, 02447, Republic of Korea

[4]Department of Information Display, Kyung Hee University, Seoul, 02447, Republic of Korea

*Corresponding author. Email: mh.cho@yonsei.ac.kr (M.-H.C.)

Contents

1. Sample characterization of $Cr_{1+\delta}Te_2$ 3

1.1 $Cr_{1+\delta}Te_2$ characterization 3

1.2 $Cr_{1.612}Te_2$ characterization 6

1.3 $Cr_{1.468}Te_2$ characterization 9

1.4 $Cr_{1.357}Te_2$ characterization 11

1.5 $Cr_{1.238}Te_2$ characterization 13

1.6 Conductivity curve data of $Cr_{1+\delta}Te_2$ with δ =0.612, 0.468, 0.357, and 0.238 15

2. Tanh function fitting details of $Cr_{1+\delta}Te_2$ for different temperatures to extract AHE and THE contributions 16

2.1 Fitting catastrophe 16

2.2 Fitting detail for $Cr_{1.612}Te_2$ 20

2.3 Fitting detail for $Cr_{1.468}Te_2$ 21

2.4 Fitting detail for $Cr_{1.357}Te_2$ 24

2.5 Fitting detail for $Cr_{1.238}Te_2$ 27

2.6 Summary of AHE amplitude and coercivity for $Cr_{1+\delta}Te_2$ with $\delta = 0.612$, 0.468, 0.357, and 0.238. . 29

3. Excluding the possibility of mimic-THE in $Cr_{1+\delta}Te_2$ 31

3.1 Mimic-THE: two-AHE model 31

3.2 Kneller's law fitting of $H_C(T)$ for $Cr_{1+\delta}Te_2$ 32

3.3 two-AHE model fitting for $Cr_{1.612}Te_2$ 37

3.4 two-AHE model fitting for $Cr_{1.238}Te_2$ 44

4. Chiral spin texture in $Cr_{1+\delta}Te_2$ 48

5. Non-monotonic temperature-dependent THE amplitude in $Cr_{1.612}Te_2$ 50

6. The ρ_{xy} of lateral $Cr_2Te_3/CrTe_2$ with broken inversion symmetry at the interface 52

Section 1. Sample characterization of $Cr_{1+\delta}Te_2$

1.1 $Cr_{1+\delta}Te_2$ characterization

$Cr_{1+\delta}Te_2$ is not an alloy but a crystal with stable phases depending on the value of δ, which can range from 0 to 1. With the recent surge in research on 2D ferromagnets (FMs), there has been significant interest in $CrTe_2$, a 2D van der Waals (vdW) ferromagnetic crystal corresponding to $\delta = 0$. $CrTe_2$ can form various superstructures through the self-intercalation of Cr atoms in the vdW gaps, and its magnetic properties change with the degree of intercalation in the change of crystal structure.[1] However, growing $Cr_{1+\delta}Te_2$ homogeneously over large areas is quite challenging, and even when grown using chemical vapor deposition (CVD), it may form a multi-phase structure rather than a single phase.[2] In other words, stable phases are formed locally in lateral regions, creating grain boundaries with other phases. Therefore, when measuring the composition of $Cr_{1+\delta}Te_2$ using methods such as energy dispersive spectroscopy (EDS), the value of δ might appear different from the stable phase if the sample is not laterally homogeneous, depending on the portions of each phase within the measurement range. That is, $Cr_{1+\delta}Te_2$ might grow with locally dominant phases when excluding the presence of defects or vacancies. In such cases, the domain boundaries are naturally inversion asymmetric, and films with many domain boundaries, which indicate poor lateral homogeneity, may appear as if Cr atoms are intercalated in random positions. Hence, this study aims to investigate the THE characteristics manifested in pristine $Cr_{1+\delta}Te_2$ with stable phases by excluding non-centrosymmetry caused by the factors mentioned above. To do this, we used $Cr_{1+\delta}Te_2$ that is close to the stable phases (e.g., CrTe, Cr_3Te_4, Cr_2Te_3, and Cr_5Te_8) with lateral homogeneity grown on sapphire substrates. Moreover, the THE phenomenon has been reported to occur at the interface of 2D FM and heavy metals due to interfacial non-centrosymmetry and strong spin-orbit coupling, which induce chiral spin textures via the interfacial DMI (iDMI). However, in

this work, we focused on the unique and straightforward tool of self-intercalation in $Cr_{1+\delta}Te_2$ to control crystal symmetry and bulk DMI. We investigated the THE characteristics in pristine $Cr_{1+\delta}Te_2$ depending on the amount of Cr intercalant. Therefore, we can automatically exclude THE induced by iDMI. Furthermore, mimic-THE caused by magnetic inhomogeneity (e.g., vertical inhomogeneity due to strain from lattice mismatch with the substrate) has been reported. To prevent this, as mentioned in the main manuscript, we ensured that all $Cr_{1+\delta}Te_2$ samples had an appropriate thickness of 7 to 8 nm.

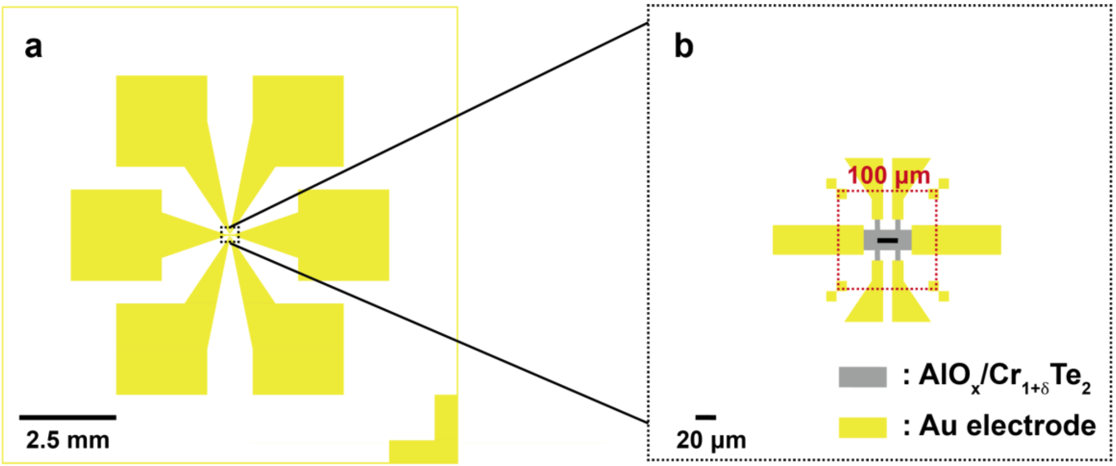

Figure S1. Schematic of the standard Hall bar device used in this work. a) Schematic of the photomask used to make standard Hall bar device with dimensions of 1 cm × 1 cm. b) Enlarged view of the boxed area indicated by the dotted lines in (a). The transport channel, composed of $AlO_x/Cr_{1+\delta}Te_2$ and represented by a gray box, has dimensions of 20 μm × 20 μm. The Au electrodes are represented by yellow boxes. The central area of 100 μm × 100 μm for EDS measurements is indicated by a red dotted box.

Table S1. Summary for the $Cr_{1+\delta}Te_2$ growth conditions.

Samples	Substrate temperature	$Flux_{Cr}$	$Flux_{Cr}$: $Flux_{Te}$
$Cr_{1.612}Te_2$	360 °C	~ 0.06 nm/min	1:6.03
$Cr_{1.468}Te_2$	260 °C	~ 0.05 nm/min	1:2.13
$Cr_{1.357}Te_2$	300 °C	~ 0.05 nm/min	1:3.03
$Cr_{1.238}Te_2$	200 °C	~ 0.05 nm/min	1:2.71

To verify the lateral homogeneity of pristine $Cr_{1+\delta}Te_2$, EDS was used to confirm stoichiometric homogeneity in at least two regions of 100 μm × 100 μm: 1) the central part where the transport channel of the standard 20 μm × 20 μm Hall bar pattern is located, and 2) an area without the Hall bar pattern, as shown in **Figure S1**. Only $Cr_{1+\delta}Te_2$ films with stoichiometry differences of less than 1 percentage point between the measured regions were utilized, ensuring the characterization of single-phase pristine $Cr_{1+\delta}Te_2$ without domain boundaries. To grow the $Cr_{1+\delta}Te_2$ with targeted δ, the temperature of the sapphire substrates during deposition, the temperature of the Knudsen cell used for evaporating Cr, and the ratio of Cr flux to Te flux were delicately adjusted. In addition to achieving stoichiometric accuracy, considerable effort was made to optimize the surface roughness and crystallinity of the films, as shown in SI sections 1.2 to 1.5. The conditions used for optimized growth of $Cr_{1+\delta}Te_2$ according to delta are summarized in Table S1. As the amount of Cr intercalant increases (or the relative amount of Te decreases), such as in $Cr_{1.612}Te_2$, high-temperature growth is essential. However, for $Cr_{1.468}Te_2$, which is close to Cr_2Te_3, it was necessary to grow the film at relatively lower temperatures and with less Te flux to achieve lateral homogeneity, deviating from the general trend for growing $Cr_{1+\delta}Te_2$ in this study. Sections 1.2 to 1.5 below include characterization data for the aforementioned optimal pristine $Cr_{1+\delta}Te_2$.

1.2 $Cr_{1.612}Te_2$ characterization

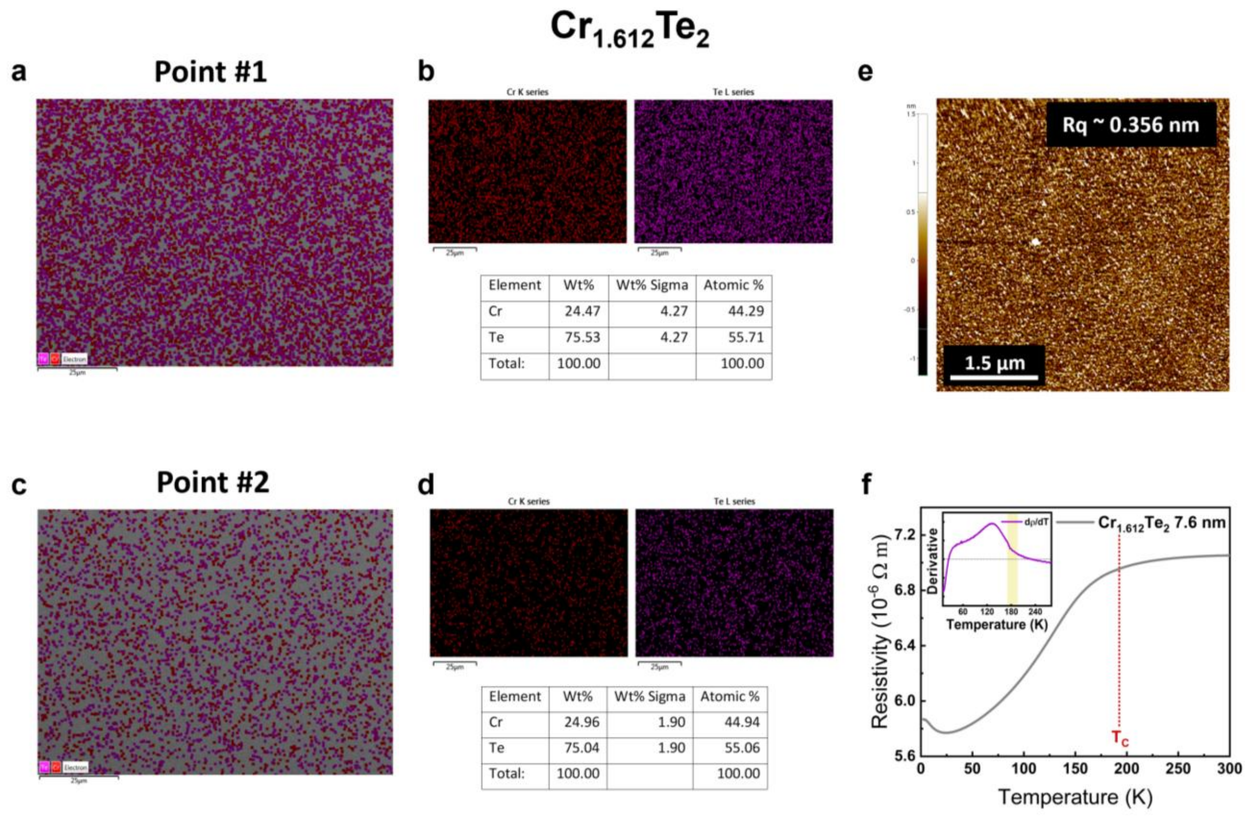

Figure S2. EDS, AFM data, and *R*–*T* curve data of $Cr_{1.612}Te_2$ 7.6 nm. a) Top-view EDS layered image of point #1 in $Cr_{1.612}Te_2$. b) EDS elemental mapping of $Cr_{1.612}Te_2$ (top-view) with 25 μm scale bar. Atomic percentages of Cr and Te are calculated based on the K and L series spectrum respectively. c) Top-view EDS layered image of point #2 in $Cr_{1.612}Te_2$. d) EDS elemental mapping of $Cr_{1.612}Te_2$ (top-view) with 25 μm scale bar. e) AFM image of $Cr_{1.612}Te_2$ (with AlO_x capping) with 1.5 μm scale bar. f) *R*–*T* curve data of $Cr_{1.612}Te_2$. Inset: First-order derivative of the *R*–*T* curve.

The EDS layered images of the two regions mentioned in Figure S1 for $Cr_{1.612}Te_2$ 7.6 nm are shown in **Figure S2**a and c. The EDS elemental mapping and atomic concentration

information are provided in Figure S2b and d. In Figure S2b and d, the atomic concentrations of Cr and Te atoms were determined using the K and L series of the EDS spectrum, respectively, and the quantitative values are summarized in a table in the bottom panel. Since the differences in the measured weight percentages of Cr and Te atoms between the two regions are smaller than the weight percentage error of the FE-SEM (approximately 0.5%) used in this study, the sample is considered laterally homogeneous over a large area. By averaging the atomic concentrations of the two regions, the δ of $Cr_{1+\delta}Te_2$ can be determined, with this sample identified as $Cr_{1.612}Te_2$.

AFM measurements were conducted to measure the surface flatness of $Cr_{1.612}Te_2$ with an AlO_x capping layer, as shown in Figure S2e. The $Cr_{1.612}Te_2$ 7.6 nm film with an AlO_x 2 nm capping layer exhibits flat surfaces with a surface roughness of approximately 0.356 nm. The *R*–*T* curve of $Cr_{1.612}Te_2$, which is the same as Figure 2b, is represented in Figure S2f, showing a ferromagnetic transition near the temperature of 195 K. The resistivity of $Cr_{1.612}Te_2$ at 300 K is confirmed to be approximately $7.05\times 10^{-6}\ \Omega \cdot m$.

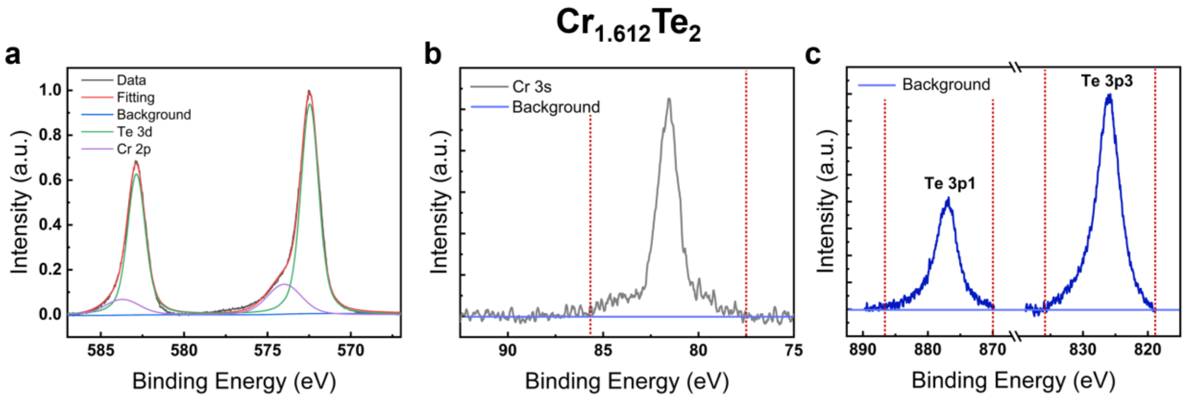

d

Atomic Concentration Table

	C1s	O1s	Cr2p	Cr3s	Te3p3	Te3p1	Te4d	SUR	
	0.314	0.733	2.488	0.080	1.750	0.794	1.721	1.000	RSF
	5.877	14.612	54.792	1.422	40.310	18.506	33.875	116.275	CorrectedRSF
	0.02	0.00	0.00	47.28	25.03	27.68	0.00	0.00	

e

[XPS] $$\frac{27.68\ \%\ (\text{Te 3p1})+25.03\%\ (\text{Te 3p3})}{47.28\ \%\ (\text{Cr 3s})} = 1.1148$$

[EDS] $$2/1.612 = 1.241$$

Figure S3. In situ XPS data of $Cr_{1.612}Te_2$ 7.6 nm. a) In situ XPS spectra of $Cr_{1.612}Te_2$ showing Cr 2p and Te 3d curves. The background is indicated by the cool blue solid line. b) XPS spectra of Cr 3s. c) XPS spectra of Te 3p. The regions designated for extracting atomic concentration are marked with a red-dotted line in the spectra. d) Atomic concentrations derived from the Cr 3s and Te 3p peaks. e) Comparison of the atomic concentrations derived from XPS data and those obtained from EDS measurements.

To double-check the composition of $Cr_{1.612}Te_2$ derived from EDS, we conducted in situ XPS measurements. However, the main peaks of Cr (2p) and Te (3d) in the XPS spectra overlap within the range of approximately 1 eV, making it difficult to extract accurate atomic concentrations from these peaks. Therefore, to obtain precise atomic concentrations, we used the Cr 3s and Te 3p peaks instead of the main peaks, as shown in **Figure S3**b, c. Despite this approach, Figure S3b,c reveals that the signal-to-noise ratio (SNR) for the Cr 3s and Te 3p peaks is lower compared to the main peaks, which could lead to inaccurate calculations. The atomic concentrations derived from the Cr 3s and Te 3p peaks are summarized in a table in Figure S3d and compared with the concentrations calculated using EDS in Figure S3e. The comparison in Figure S3e shows the ratio of Te concentration relative to Cr concentration. While there are slight differences between the results obtained from EDS and XPS, both methods confirm that the sample is close to a Cr-rich composition of $Cr_{1+\delta}Te_2$, such as CrTe or Cr_5Te_6.

1.3 $Cr_{1.468}Te_2$ characterization

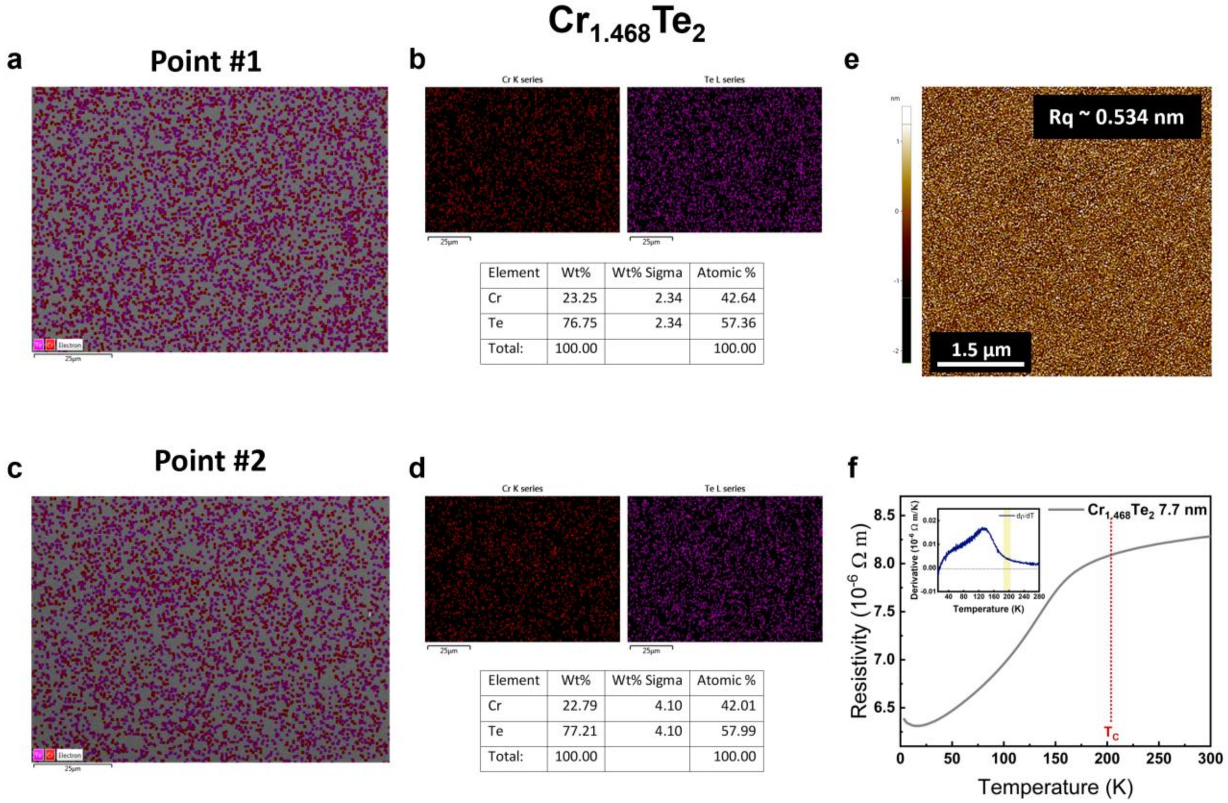

Element	Wt%	Wt% Sigma	Atomic %
Cr	23.25	2.34	42.64
Te	76.75	2.34	57.36
Total:	100.00		100.00

Element	Wt%	Wt% Sigma	Atomic %
Cr	22.79	4.10	42.01
Te	77.21	4.10	57.99
Total:	100.00		100.00

Figure S4. EDS, AFM data, and *R*–*T* curve data of $Cr_{1.468}Te_2$ 7.7 nm. a) Top-view EDS layered image of point #1 in $Cr_{1.468}Te_2$. b) EDS elemental mapping of $Cr_{1.468}Te_2$ (top-view) with 25 μm scale bar. Atomic percentages of Cr and Te are calculated based on the K and L series spectrum respectively. c) Top-view EDS layered image of point #2 in $Cr_{1.468}Te_2$. d) EDS elemental mapping of $Cr_{1.468}Te_2$ (top-view) with 25 μm scale bar. e) AFM image of $Cr_{1.468}Te_2$ (with AlO_x capping) with 1.5 μm scale bar. f) *R*–*T* curve data of $Cr_{1.468}Te_2$. Inset: First-order derivative of the *R*–*T* curve.

The EDS layered images of the two regions mentioned in Figure S1 for $Cr_{1.468}Te_2$ 7.7 nm are shown in **Figure S4**a and c. The EDS elemental mapping and atomic concentration

information are provided in Figure S4b and d. Since the differences in the measured weight percentages of Cr and Te atoms between the two regions are smaller than the weight percentage error of the FE-SEM (approximately 0.5%) used in this study, the sample is considered laterally homogeneous over a large area. By averaging the atomic concentrations of the two regions, the δ of $Cr_{1+\delta}Te_2$ can be determined, with this sample identified as $Cr_{1.468}Te_2$.

AFM measurements were conducted to measure the surface flatness of $Cr_{1.468}Te_2$ with an AlO_x capping layer, as shown in Figure S4e. The $Cr_{1.468}Te_2$ 7.7 nm film with an AlO_x 2 nm capping layer exhibits flat surfaces with a surface roughness of approximately 0.534 nm. The R–T curve of $Cr_{1.468}Te_2$ is presented in Figure S4f, showing a ferromagnetic transition near 205 K. Additionally, the first derivative of the R–T curve changes sharply between 190 K and 210 K, indicating that the T_C of $Cr_{1.468}Te_2$ is approximately 205 K, as shown in the inset of Figure S4f. The resistivity of $Cr_{1.468}Te_2$ at 300 K is confirmed to be approximately $8.26\times 10^{-6}\ \Omega \cdot m$.

1.4 $Cr_{1.357}Te_2$ characterization

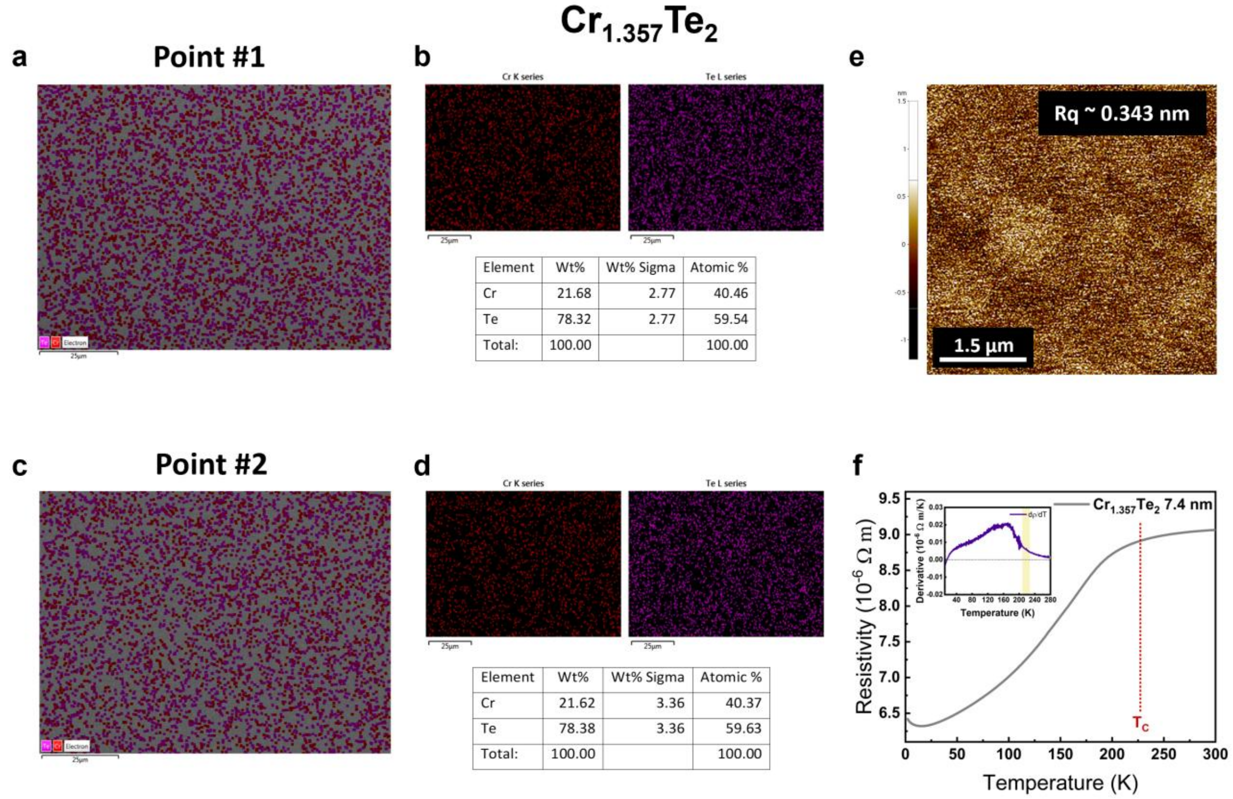

Figure S5. EDS, AFM data, and *R*–*T* curve data of $Cr_{1.357}Te_2$ 7.4 nm. a) Top-view EDS layered image of point #1 in $Cr_{1.357}Te_2$. b) EDS elemental mapping of $Cr_{1.357}Te_2$ (top-view) with 25 μm scale bar. Atomic percentages of Cr and Te are calculated based on the K and L series spectrum respectively. c) Top-view EDS layered image of point #2 in $Cr_{1.357}Te_2$. d) EDS elemental mapping of $Cr_{1.357}Te_2$ (top-view) with 25 μm scale bar. e) AFM image of $Cr_{1.357}Te_2$ (with AlO_x capping) with 1.5 μm scale bar. f) *R*–*T* curve data of $Cr_{1.357}Te_2$. Inset: First-order derivative of the *R*–*T* curve.

The EDS layered images of the two regions mentioned in Figure S1 for $Cr_{1.357}Te_2$ 7.4 nm are shown in **Figure S5**a and c. The EDS elemental mapping and atomic concentration

information are provided in Figure S5b and d. Since the differences in the measured weight percentages of Cr and Te atoms between the two regions are smaller than the weight percentage error of the FE-SEM (approximately 0.5%) used in this study, the sample is considered laterally homogeneous over a large area. By averaging the atomic concentrations of the two regions, the δ of $Cr_{1+\delta}Te_2$ can be determined, with this sample identified as $Cr_{1.357}Te_2$.

AFM measurements were conducted to measure the surface flatness of $Cr_{1.357}Te_2$ with an AlO_x capping layer, as shown in Figure S5e. The $Cr_{1.357}Te_2$ 7.7 nm film with an AlO_x 2 nm capping layer exhibits flat surfaces with a surface roughness of approximately 0.343 nm. The *R*–*T* curve of $Cr_{1.357}Te_2$ is presented in Figure S5f, showing ferromagnetic transition near 225 K. Moreover, the first derivative of the *R*–*T* curve changes sharply between 210 K and 220 K, indicating that the T_C of $Cr_{1.357}Te_2$ is approximately 225 K, as shown in the inset of Figure S5f. The resistivity of $Cr_{1.357}Te_2$ at 300 K is confirmed to be approximately $9.09\times 10^{-6}\ \Omega \cdot m$.

1.5 $Cr_{1.238}Te_2$ characterization

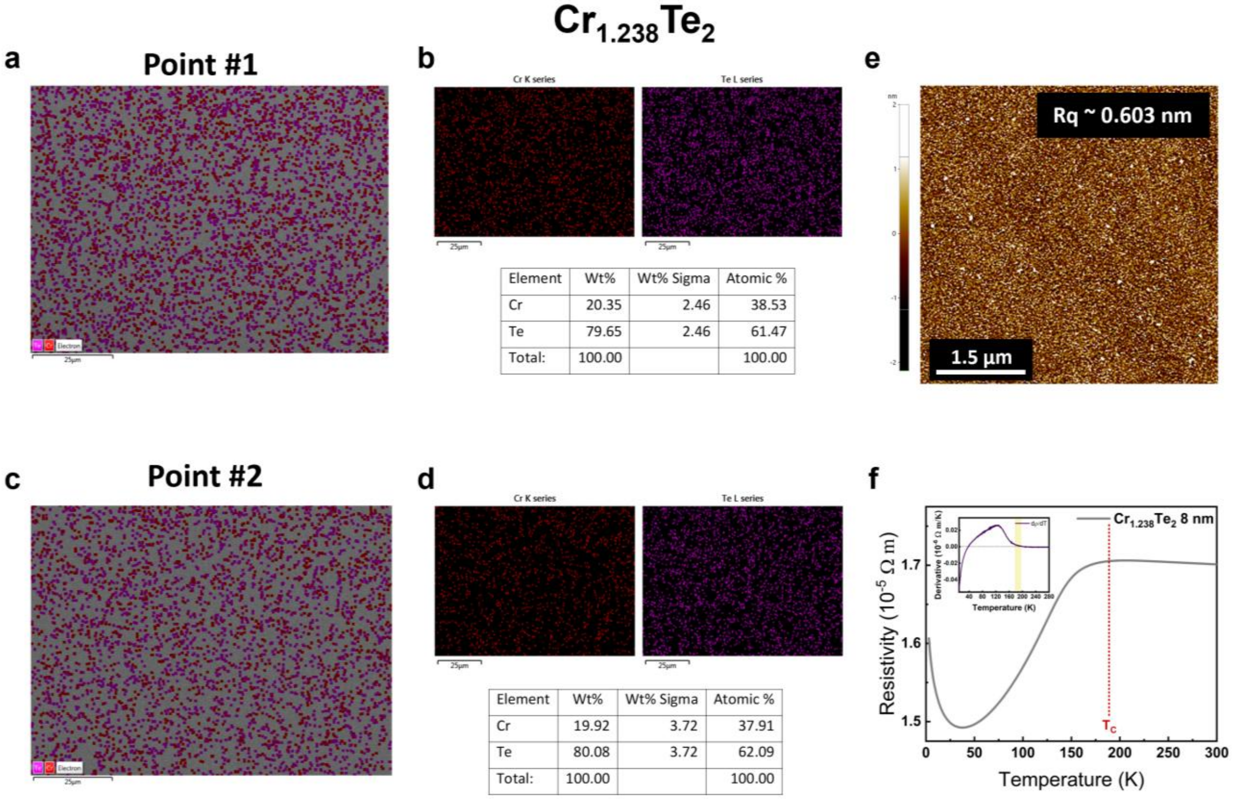

Figure S6. EDS, AFM data, and *R*–*T* curve data of $Cr_{1.238}Te_2$ 8 nm. a) Top-view EDS layered image of point #1 in $Cr_{1.238}Te_2$. b) EDS elemental mapping of $Cr_{1.238}Te_2$ (top-view) with 25 μm scale bar. Atomic percentages of Cr and Te are calculated based on the K and L series spectrum respectively. c) Top-view EDS layered image of point #2 in $Cr_{1.238}Te_2$. d) EDS elemental mapping of $Cr_{1.238}Te_2$ (top-view) with 25 μm scale bar. e) AFM image of $Cr_{1.238}Te_2$ (with AlO_x capping) with 1.5 μm scale bar. f) *R*–*T* curve data of $Cr_{1.238}Te_2$. Inset: First-order derivative of the *R*–*T* curve.

The EDS layered images of the two regions mentioned in Figure S1 for $Cr_{1.238}Te_2$ 8 nm are shown in **Figure S6**a and c. The EDS elemental mapping and atomic concentration information

are provided in Figure S6b and d. Since the differences in the measured weight percentages of Cr and Te atoms between the two regions are smaller than the weight percentage error of the FE-SEM (approximately 0.5%) used in this study, the sample is considered laterally homogeneous over a large area. By averaging the atomic concentrations of the two regions, the δ of $Cr_{1+\delta}Te_2$ can be determined, with this sample identified as $Cr_{1.238}Te_2$.

AFM measurements were conducted to measure the surface flatness of $Cr_{1.238}Te_2$ with an AlO_x capping layer, as shown in Figure S6e. The $Cr_{1.238}Te_2$ 8 nm film with an AlO_x 2 nm capping layer exhibits flat surfaces with a surface roughness of approximately 0.603 nm. The *R*–*T* curve of $Cr_{1.238}Te_2$ is presented in Figure S6f, showing a ferromagnetic transition near the temperature of 190 K. Moreover, the first derivative of the *R*–*T* curve changes sharply between 180 K and 200 K, indicating that the T_C of $Cr_{1.238}Te_2$ is approximately 190 K, as shown in the inset of Figure S6f. The resistivity of $Cr_{1.238}Te_2$ at 300 K is confirmed to be approximately $1.69\times 10^{-5}\ \Omega\cdot m$.

1.6 Conductivity curve data of $Cr_{1+\delta}Te_2$ with $\delta = 0.612$, 0.468, 0.357, and 0.238.

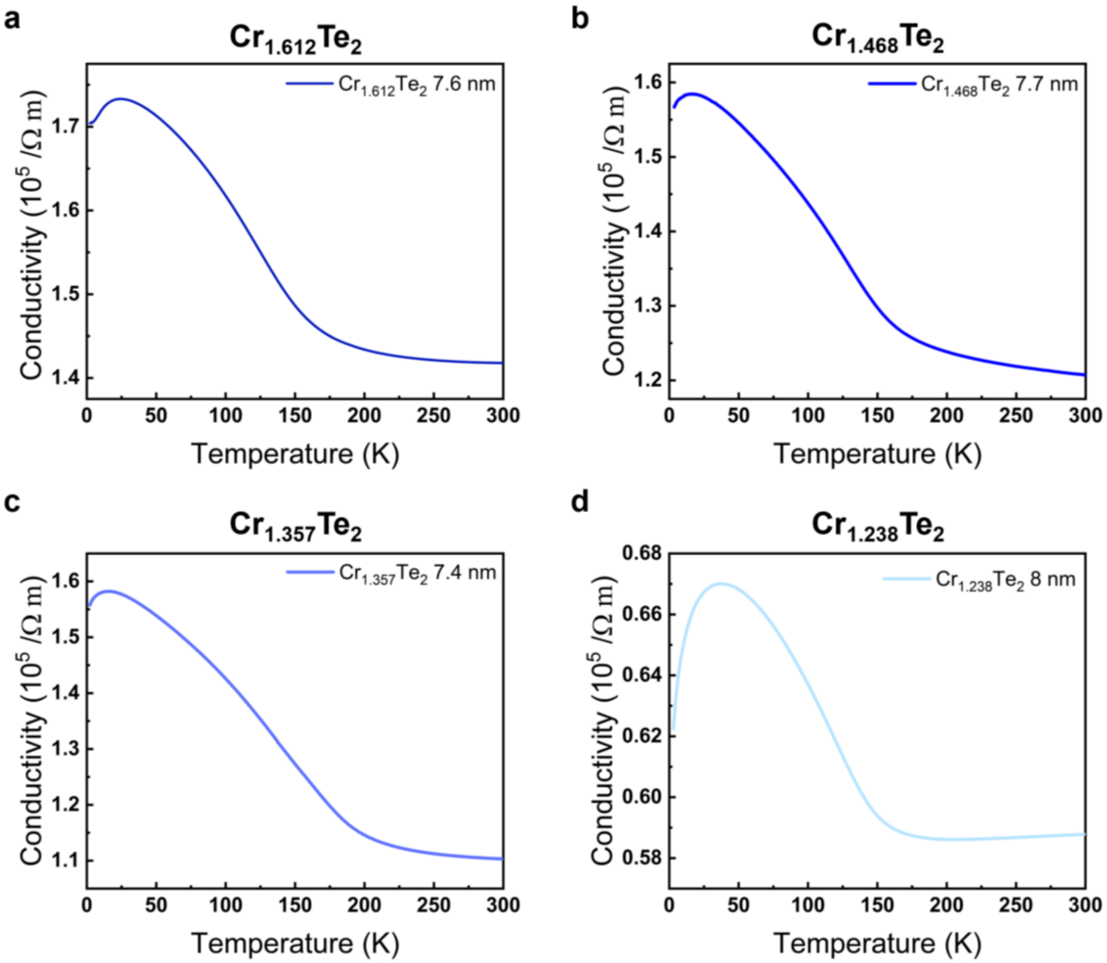

Figure S7. Conductivity curve data. a) Conductivity curve as a function of temperature for $Cr_{1.612}Te_2$ 7.6 nm. b) Conductivity curve as a function of temperature for $Cr_{1.468}Te_2$ 7.7 nm. c) Conductivity curve as a function of temperature for $Cr_{1.357}Te_2$ 7.4 nm. d) Conductivity curve as a function of temperature for $Cr_{1.238}Te_2$ 8 nm.

To figure out how the conductivity (resistivity) of $Cr_{1+\delta}Te_2$ changes with δ, **Figure S7** shows the conductivity curves as a function of temperature for $Cr_{1+\delta}Te_2$ with $\delta = 0.612$, 0.468, 0.357, 0.238. Considering the single-atom conductivities, Te has lower conductivity than Cr. Therefore, as the value of δ increases in $Cr_{1+\delta}Te_2$, the conductivity is expected to increase. As

predicted, and shown in Figure S7, the conductivity increases with δ, with values of 1.42× $10^5/\Omega \cdot m$ (for $\delta = 0.612$), 1.21× $10^5/\Omega \cdot m$ (for $\delta = 0.468$), 1.10× $10^5/\Omega \cdot m$ (for $\delta = 0.357$), and 0.59× $10^5/\Omega \cdot m$ (for $\delta = 0.238$).

Section 2. Tanh function fitting details of $Cr_{1+\delta}Te_2$ for different temperatures to extract AHE and THE contributions

2.1 Fitting catastrophe

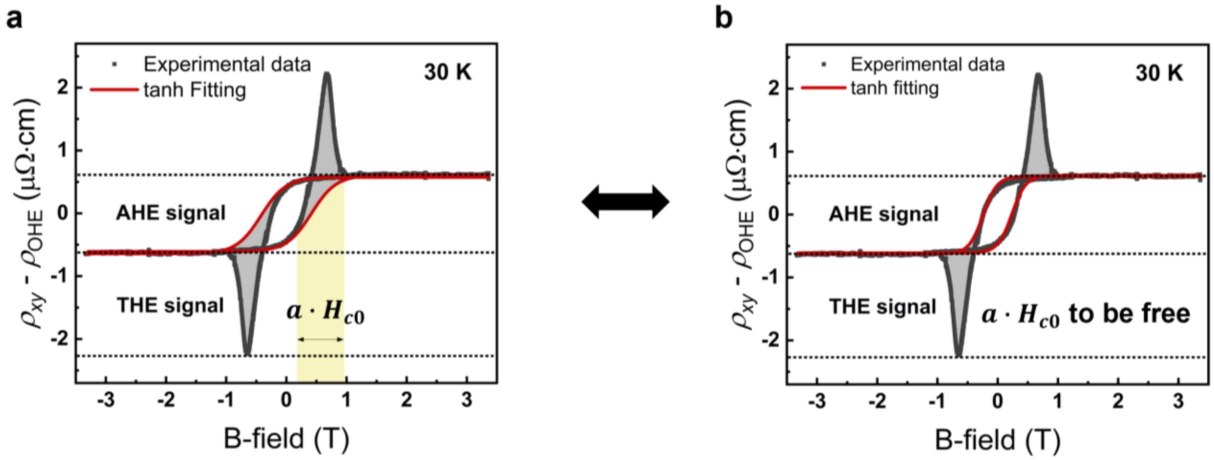

Figure S8. Two different methods of fitting the AHE contribution of $Cr_{1.612}Te_2$ based on the presence of fitting constraints. a) Fitting results of the $\rho_{xy} - \rho_{OHE}$ curve at 30 K for $Cr_{1.612}Te_2$ to separate the AHE signal with fitting constraints applied. The region representing the fitting constraint is indicated by the yellow box. b) Fitting results of the $\rho_{xy} - \rho_{OHE}$ curve at 30 K for $Cr_{1.612}Te_2$ to separate the AHE signal without applying constraints to any parameters. In both (a) and (b), the AHE signal obtained from the fitting is shown by the red solid line, and the THE signal is represented by the gray area.

With the great attention in research on THE arising from chiral spin textures, instances of incorrect separation of THE and AHE signals from the $\rho_{xy} - \rho_{OHE}$ curve have been reported.[3-5] Considering the formation of chiral spin textures (e.g., magnetic skyrmions, and non-coplanar spin textures), it is essential to note that these are formed not when the spin polarization of a magnetic material is fully saturated, but during the switching process of magnetic domains. In the case of non-coplanar spin textures, as the magnetic domains switch, the competition between DMI energy and exchange interaction energy stabilizes the non-coplanar spin texture. When a larger magnetic field is applied, magnetization aligns in the direction of the B-field. In other words, it is physically impossible for a THE signal to occur after AHE resistivity has saturated.

In general, to separate the AHE signal (ρ_{AHE}) from the mixed THE and AHE signal in the $\rho_{xy} - \rho_{OHE}$ curve, ρ_{AHE} is fitted out using the quantum mechanical approach of the $M_s \tanh\left(\frac{H}{a} - H_{c0}\right)$ function, where M_s, a, H_{c0} are fitting parameters, or by obtaining an additional magnetization curve and subtracting it. In early studies of materials such as B-20 type materials and perovskites, where chiral spin texture formation was considered, the coercivity of the AHE signal was positioned inside the field range where ρ_{THE} appears, as illustrated in **Figure S8**a; coercivity (defined as $a \cdot H_{c0}$) of AHE signal to be near the field at which ρ_{THE} reaches its peak value.[6-8] However, in recent THE studies, there have been cases where physical errors occurred during the separation of the AHE signal, either by subtracting negative sweep data from positive sweep data or by fitting the AHE signal after AHE resistivity had saturated, as shown in Figure S8b. Such approaches lead to the wrong assumption that chiral spin texture occurs after the saturation of magnetization, which is not the case. Therefore, as depicted in Figure S8a, accurately separating the AHE signal is essential. In detail, defining the field at

which ρ_{THE} reaches its peak value as H_T, the coercivity is constrained to be similar to H_T and positioned within the field range where ρ_{THE} occurs.

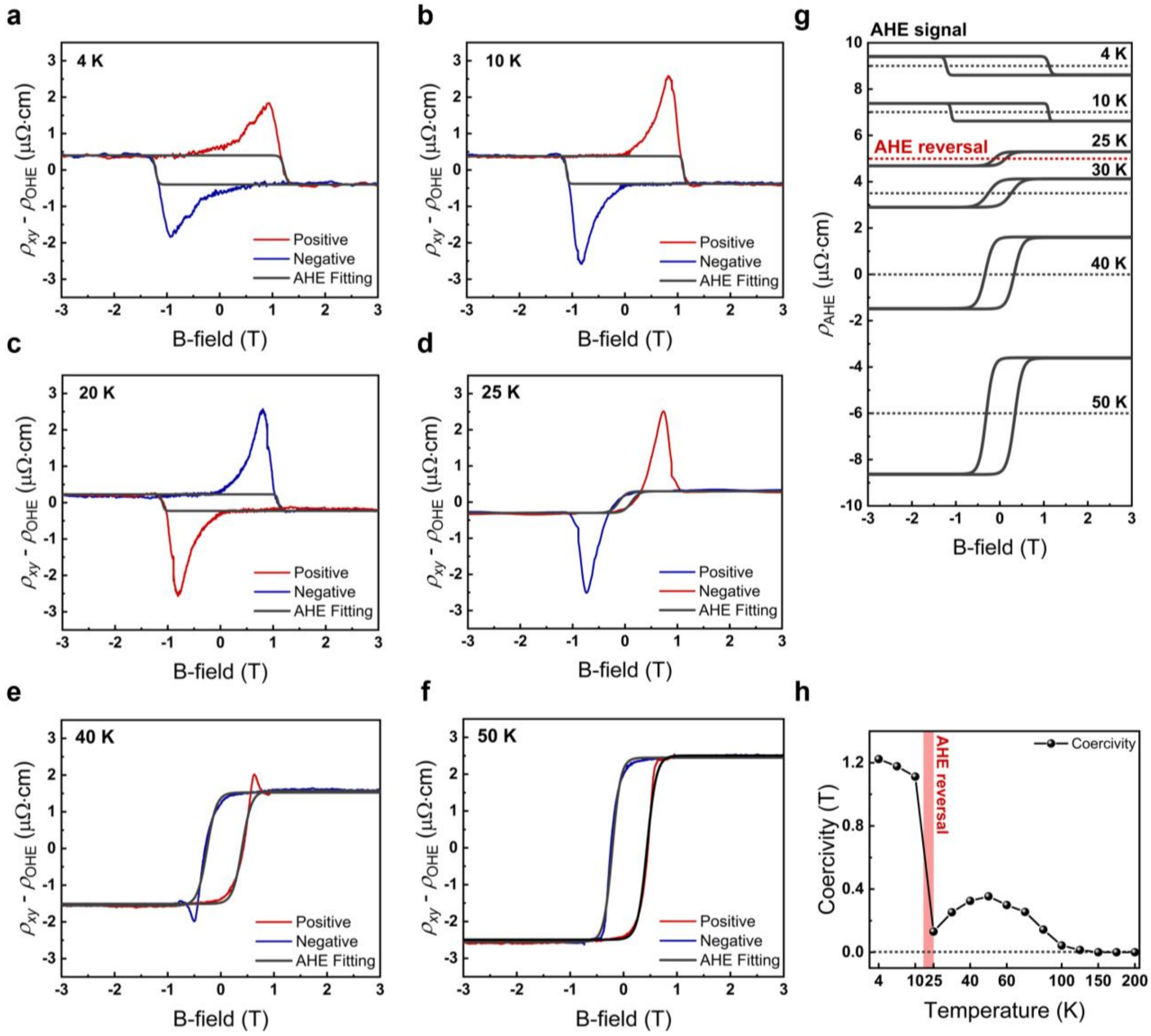

Figure S9. An example of incorrect AHE separation using the fitting method depicted in Figure S8b. a-f) $\rho_{xy} - \rho_{OHE}$ curve (≤50 K) of $Cr_{1.612}Te_2$ at different temperatures, illustrating AHE fitting details employing the method shown in Figure S8b. The positive sweep is depicted by the red solid line, the negative sweep by the blue solid line, and the fitted AHE signal is represented by the gray solid line. g) AHE signals fitted out of the data in (a)-(f) at various temperatures. h) The temperature-dependent coercivity extracted from the data in (g).

Therefore, to explain the issues arising from incorrectly separating the AHE signal using the method illustrated in Figure S8b, we attempted to fit the AHE contribution in the $\rho_{xy} - \rho_{OHE}$ curve (≤50 K) of $Cr_{1.612}Te_2$ as shown in **Figure S9**a-f. In other words, we tried to fit ρ_{AHE} without any fitting constraint, and it can be observed that for the 4, 10, and 20 K data, which overall exhibit negative polarity AHE loops, the coercivity is significantly larger than that obtained by accurately fitting ρ_{AHE} using the correct method shown in Figure 2f (or SI section 2.2), as shown in Figure S9g,h. On the other hand, for the 25, 40, and 50 K data, which exhibit positive polarity AHE loops, it can be observed that the coercivity is smaller than that obtained by accurate fitting of ρ_{AHE}. In particular, the coercivity of the fitted ρ_{AHE} exhibits a sharp decrease at 25 K where AHE reversal occurs. As a result, Figure S9h, which summarizes the coercivity for each temperature, shows physically impossible behavior regarding the coercivity of $Cr_{1.612}Te_2$. The coercivity decreases sharply around the temperature where AHE reversal occurs, increases up to 50 K, and then decreases again. This phenomenon is impossible for a magnetic material with a single domain. The results obtained by separating the AHE signal considering the conditions for the formation of chiral spin texture, as shown in Figure 2j of the main manuscript, are completely different from those obtained without applying constraints to the tanh function as in Figure S8b. Therefore, if the AHE signal is separated without applying the constraint to the tanh function, as shown in Figure S8b, it leads to a catastrophic issue with coercivity. Therefore, fitting the AHE and THE signals accurately using the method depicted in Figure S8a is essential, and we have provided the results and fitting details of separating AHE and THE for all $Cr_{1+\delta}Te_2$ used in this work in SI section 2.2 to 2.5 according to δ.

2.2 Fitting detail for $Cr_{1.612}Te_2$

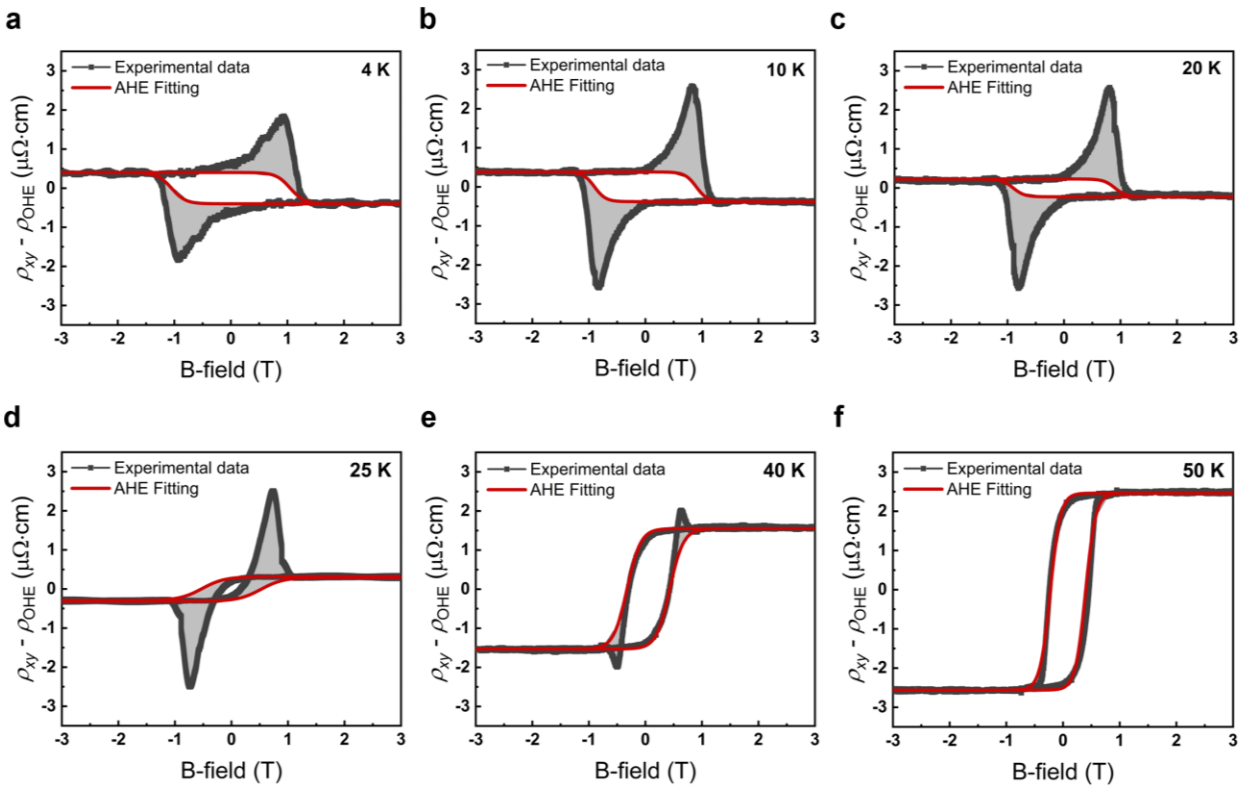

Figure S10. Fitting details of separating AHE and THE signals for $Cr_{1.612}Te_2$ 7.6 nm using the fitting method considering the formation of chiral spin texture. a-f) AHE fitting details of $Cr_{1.612}Te_2$ at 4, 10, 20, 25, 40, and 50 K, respectively. ρ_{AHE} is represented by the red solid line, and ρ_{THE} is represented by the gray area.

As shown in Figure 2e of the main manuscript, the tanh fitting details for the $\rho_{xy} - \rho_{OHE}$ curve of $Cr_{1.612}Te_2$ 7.6 nm up to the maximum temperature where THE occurs (50 K) are presented in **Figure S10**. Considering the formation of chiral spin texture as explained in SI section 2.1, the fitting was performed using the method illustrated in Figure S8a.

2.3 Fitting detail for $Cr_{1.468}Te_2$

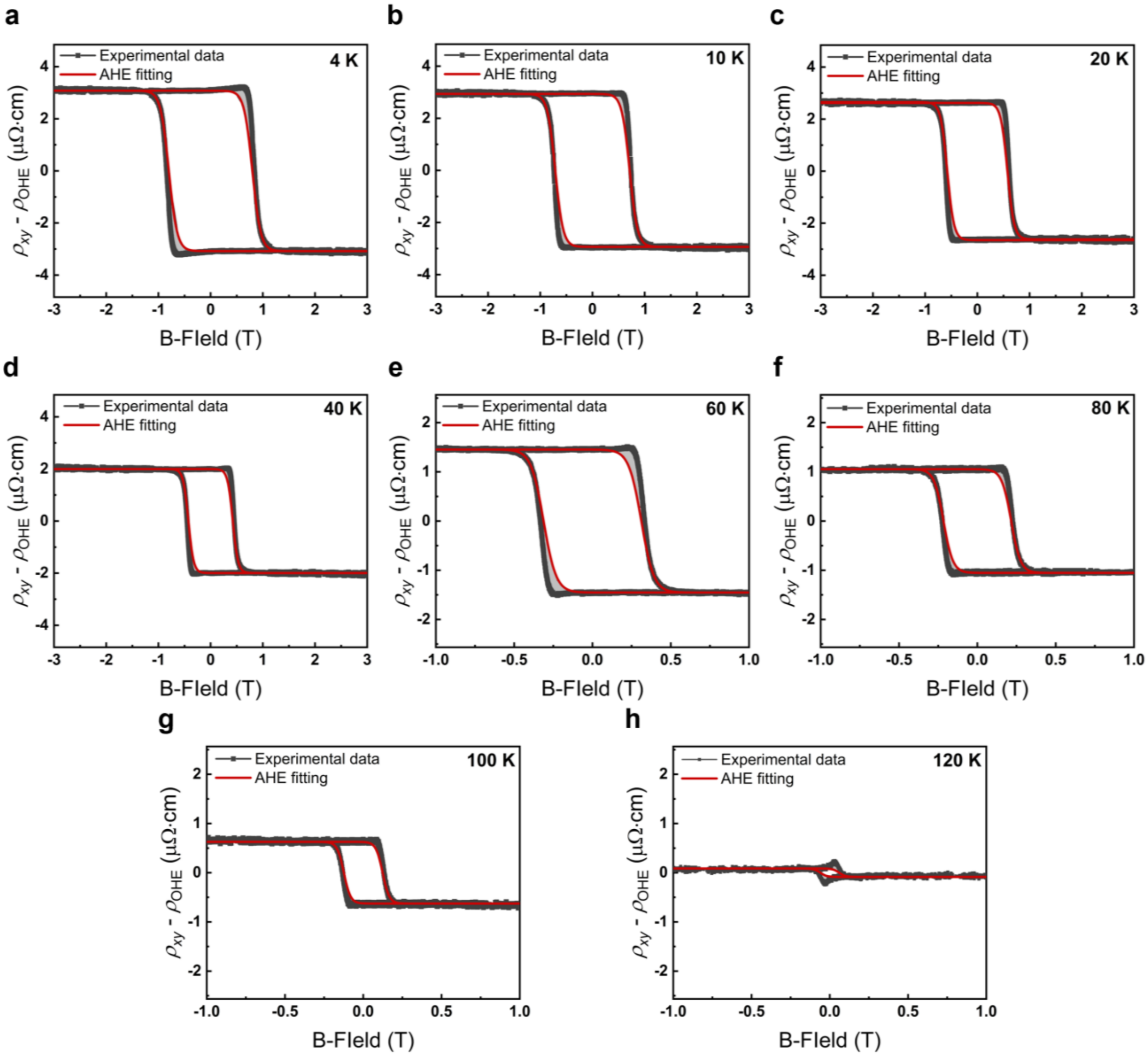

Figure S11. Fitting details of separating AHE and THE signals for $Cr_{1.468}Te_2$ 7.7 nm using the fitting method considering the formation of chiral spin texture. a-h) AHE fitting detail of $Cr_{1.468}Te_2$ at 4, 10, 20, 40, 60, 80, 100, and 120 K, respectively. ρ_{AHE} is represented by the red solid line, and ρ_{THE} is represented by the gray area.

As shown in Figure 3a of the main manuscript, the tanh fitting details for $Cr_{1.468}Te_2$ 7.7 nm up to the maximum temperature at which THE occurs (120 K) are presented in **Figure S11**. The fitting was performed using the method described in Figure S8a, considering the formation of chiral spin texture as explained in SI section 2.1.

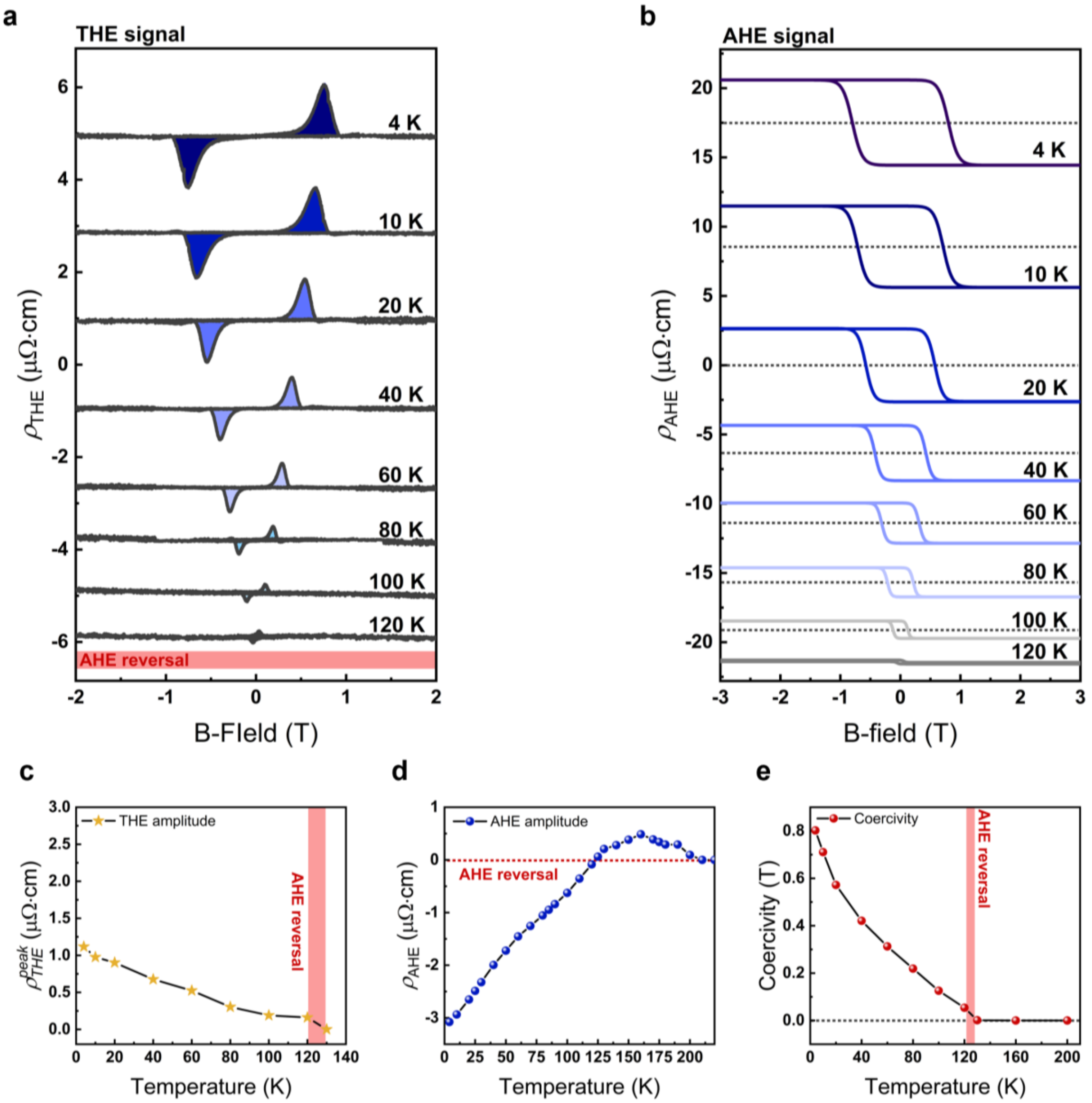

Figure S12. THE, AHE, and coercivity characteristics extracted from the data of Figure S11 for $Cr_{1.468}Te_2$ 7.7 nm. a) THE signal of the data in Figure S11 at various temperatures. b) AHE signal of the data in Figure S11 at various temperatures. c) Temperature-dependent ρ_{THE}^{peak}

of $Cr_{1.468}Te_2$. d) Temperature-dependent AHE amplitude of $Cr_{1.468}Te_2$. $\rho_{AHE} = 0$ is represented with a red dotted line. AHE polarity is reversed near 125 K. e) Temperature-dependent coercivity of $Cr_{1.468}Te_2$.

By fitting the $\rho_{xy} - \rho_{OHE}$ curve of $Cr_{1.468}Te_2$ with the tanh function in Figure S11, the values of ρ_{THE}, ρ_{AHE}, ρ_{THE}^{peak}, AHE amplitude, and coercivity were obtained and summarized as a function of temperature in **Figure S12**a to e, respectively. As shown in Figure S12c, $Cr_{1.468}Te_2$ 7.7 nm exhibits a maximum ρ_{THE}^{peak} of 1.12 $\mu\Omega \cdot cm$ at 4 K. As shown in Figure S12d, $Cr_{1.468}Te_2$ 7.7 nm also shows AHE reversal like $Cr_{1.612}Te_2$ with the polarity of the AHE signal changing sign at approximately 125 K. Moreover, the overall shape of the AHE resistivity as a function of temperature is similar to that of $Cr_{1.612}Te_2$. As shown in Figure S12e, the coercivity of $Cr_{1.468}Te_2$ is approximately 0.802 T at 4 K.

2.4 Fitting detail for $Cr_{1.357}Te_2$

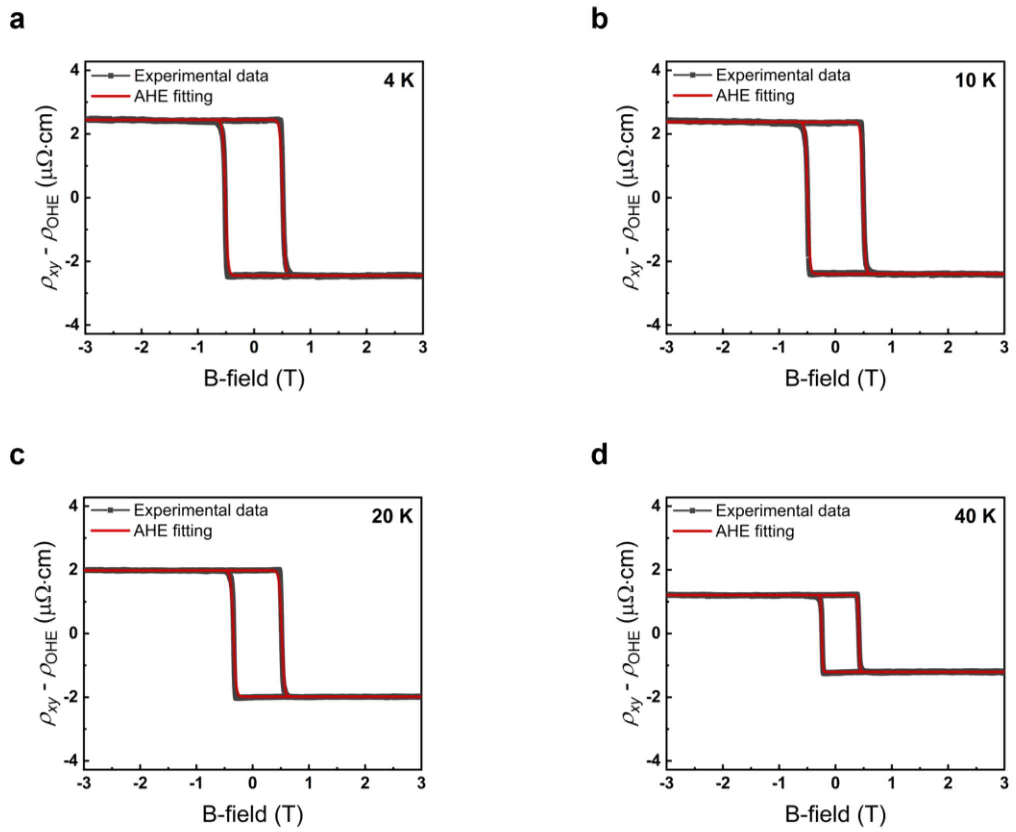

Figure S13. Fitting details of separating AHE and THE signals for $Cr_{1.357}Te_2$ 7.4 nm using the fitting method considering the formation of chiral spin texture. a-d) AHE fitting details of $Cr_{1.357}Te_2$ at 4, 10, 20, and 40 K, respectively. ρ_{AHE} is represented by the red solid line, and ρ_{THE} is represented by the gray area.

As shown in Figure 3b of the main manuscript, the tanh fitting details for $Cr_{1.357}Te_2$ 7.4 nm up to the maximum temperature at which THE occurs (40 K) are presented in **Figure S13**. The fitting was performed using the method described in Figure S8a, considering the formation of chiral spin texture as explained in SI section 2.1.

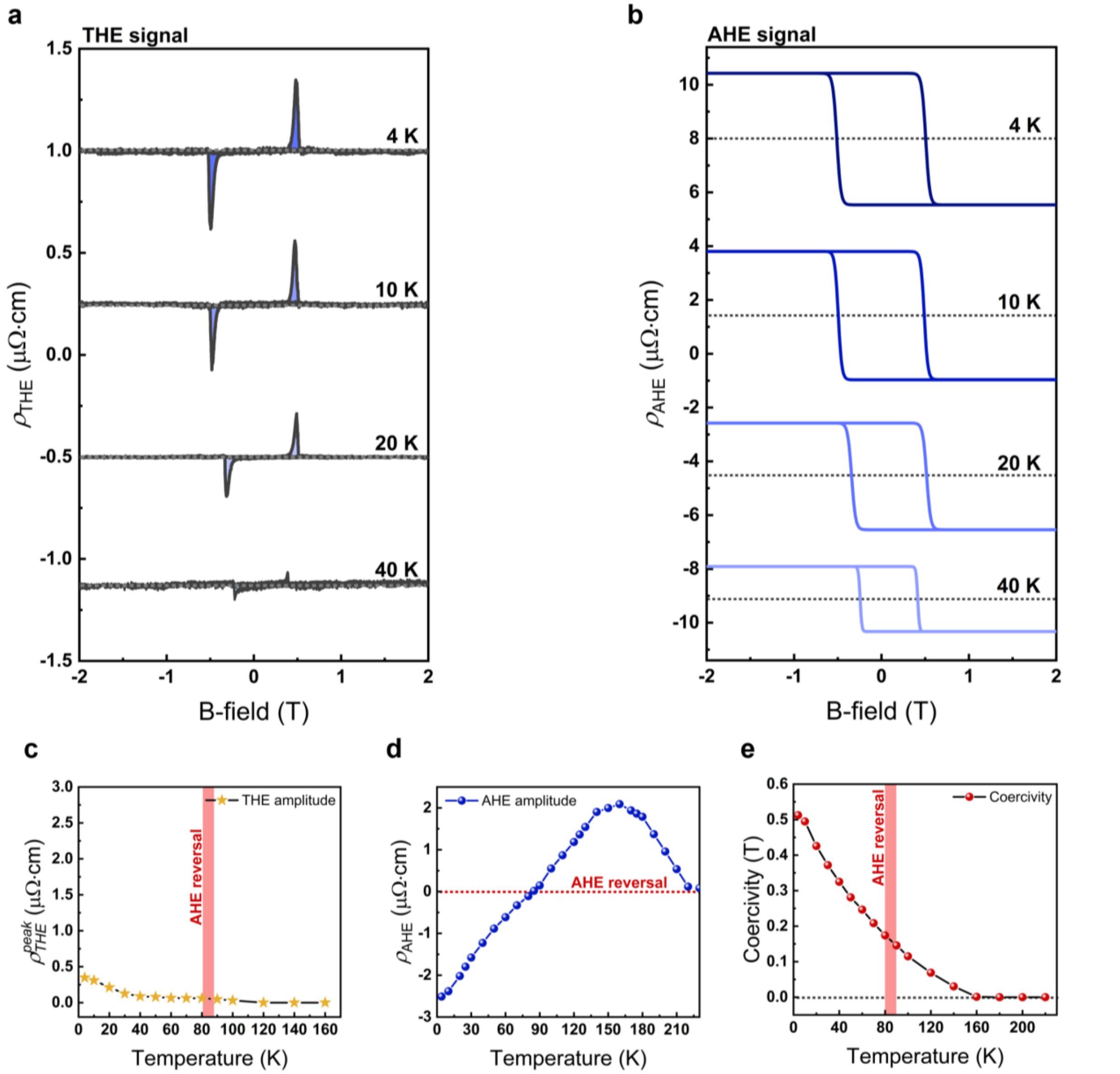

Figure S14. THE, AHE, and coercivity characteristics extracted from the data in Figure S13 for $Cr_{1.357}Te_2$ 7.4 nm. a) THE signal of the data in Figure S13 at various temperatures. b) AHE signal of the data in Figure S13 at various temperatures. c) Temperature-dependent ρ_{THE}^{peak} of $Cr_{1.357}Te_2$. d) Temperature-dependent AHE amplitude of $Cr_{1.357}Te_2$. $\rho_{AHE} = 0$ is represented with a red dotted line. AHE polarity is reversed near 85 K. e) Temperature-dependent coercivity of $Cr_{1.357}Te_2$.

The values of ρ_{THE}, ρ_{AHE}, ρ_{THE}^{peak}, AHE amplitude, and coercivity obtained by fitting the $\rho_{xy} - \rho_{OHE}$ curve of $Cr_{1.357}Te_2$ with the tanh function in Figure S13 are summarized by temperature in **Figure S14**a-e, respectively. As shown in Figure S14a, the THE signal in $Cr_{1.357}Te_2$ almost disappears at 40 K. As shown in Figure S14c, $Cr_{1.357}Te_2$ 7.4 nm shows a maximum ρ_{THE}^{peak} of 0.35 $\mu\Omega \cdot cm$ at 4 K, which is significantly smaller than that of other compositions. As shown in Figure S14d, $Cr_{1.357}Te_2$ also exhibits AHE reversal like other $Cr_{1+\delta}Te_2$ compositions, with the AHE polarity changing sign around 85 K. The AHE resistivity profile is also consistent among $Cr_{1+\delta}Te_2$ compositions. As shown in Figure S14e, the coercivity of $Cr_{1.357}Te_2$ is approximately 0.512 T at 4 K.

2.5 Fitting detail for $Cr_{1.238}Te_2$

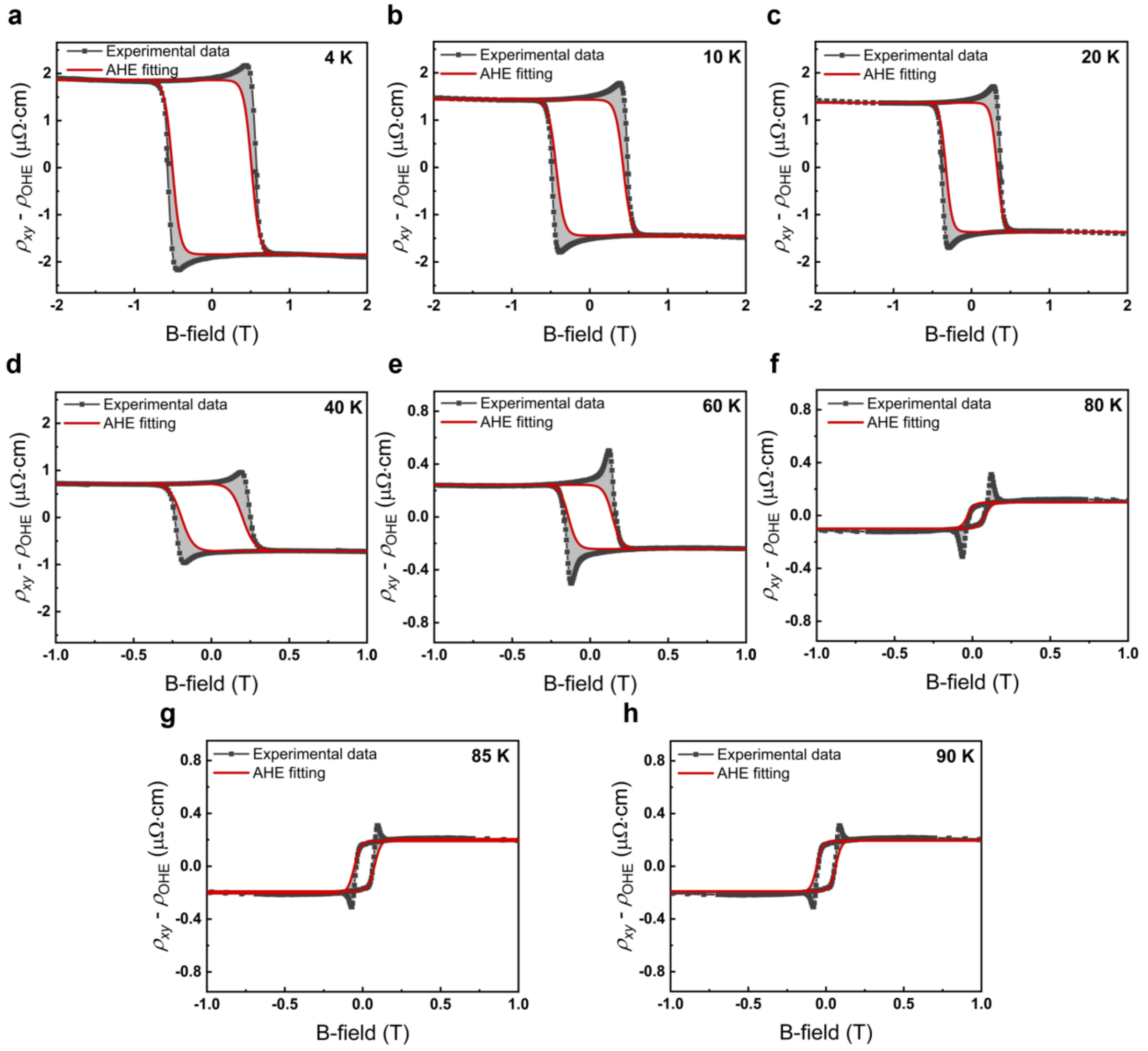

Figure S15. Fitting details of separating AHE and THE signals for $Cr_{1.238}Te_2$ 8 nm using the fitting method considering the formation of chiral spin texture. a-f) AHE fitting details of $Cr_{1.238}Te_2$ at 4, 10, 20, 40, 60, 80, 85, and 90 K, respectively. ρ_{AHE} is represented by the red solid line, and ρ_{THE} is represented by the gray area.

As shown in Figure 3c of the main manuscript, the tanh fitting details for $Cr_{1.238}Te_2$ 8 nm up to the maximum temperature at which THE occurs (90 K) are presented in **Figure S15**. The fitting was performed using the method described in Figure S8a, considering the formation of chiral spin texture as explained in SI section 2.1.

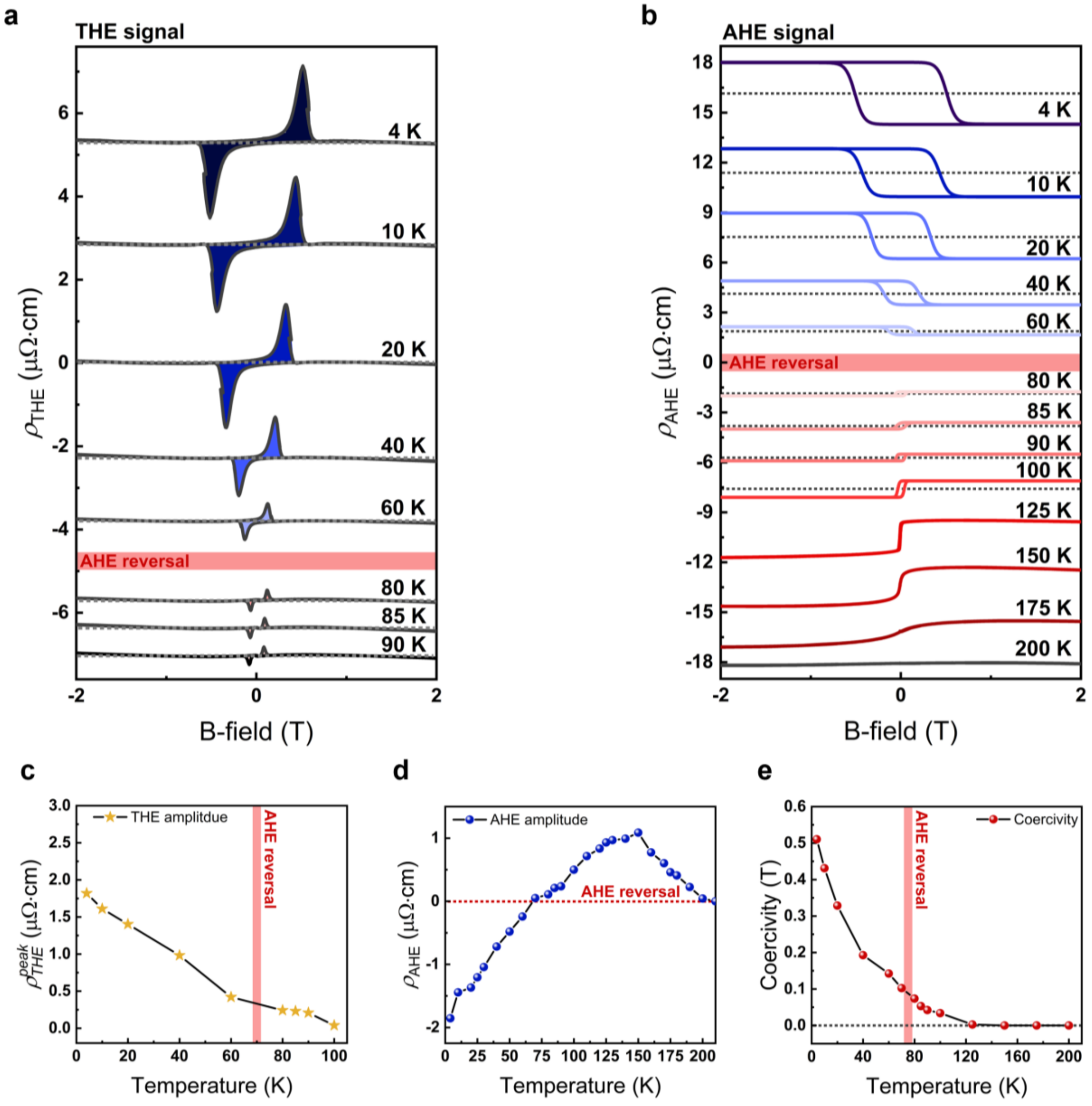

Figure S16. THE, AHE, and coercivity characteristics extracted from the data of Figure S15 for $Cr_{1.238}Te_2$ 8 nm. a) THE signal of the data in Figure S15 at various temperatures. b) AHE signal of the data in Figure S15 at various temperatures. c) Temperature-dependent ρ_{THE}^{peak}

of $Cr_{1.238}Te_2$. d) Temperature-dependent AHE amplitude of $Cr_{1.238}Te_2$. $\rho_{AHE} = 0$ is represented with a red dotted line. AHE polarity is reversed near 70 K. e) Temperature-dependent coercivity of $Cr_{1.238}Te_2$.

The values of ρ_{THE}, ρ_{AHE}, ρ_{THE}^{peak}, AHE amplitude, and coercivity obtained by fitting the $\rho_{xy} - \rho_{OHE}$ curve of $Cr_{1.238}Te_2$ with the tanh function in Figure S15 are summarized by temperature in **Figure S16**a-e, respectively. As shown in Figure S16c, $Cr_{1.238}Te_2$ 8 nm shows a maximum ρ_{THE}^{peak} of 1.82 $\mu\Omega \cdot cm$ at 4 K. Similar to other $Cr_{1+\delta}Te_2$ compositions, $Cr_{1.238}Te_2$ also exhibits AHE reversal behavior, with a change in AHE polarity occurring around 70 K, as shown in Figure S16d. Additionally, it can be observed that AHE resistivity follows a common pattern among different $Cr_{1+\delta}Te_2$ compositions. Figure S16e confirms that the coercivity of $Cr_{1.238}Te_2$ is approximately 0.510 T at 4 K.

2.6 Summary of AHE amplitude and coercivity for $Cr_{1+\delta}Te_2$ with $\delta = 0.612$, 0.468, 0.357, and 0.238.

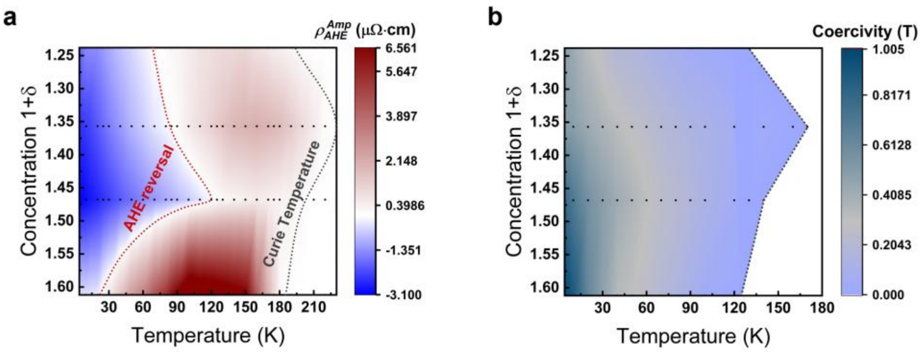

Figure S17 a) Temperature-dependent AHE amplitude values. A color contour plot is used (red-white-blue) to represent the magnitude of AHE amplitude. Setting the AHE reversal point

($\rho_{AHE} = 0$) to white, the AHE reversal points along the interpolated contour of the same white color are marked by a red-dotted line. After marking T_C according to δ, the interpolated contour is displayed as a gray-dotted line. b) Temperature-dependent coercivity. A color contour plot is used (dark cyan-gray-cool blue) to depict the coercivity magnitude.

After separating ρ_{AHE} from the $\rho_{xy} - \rho_{OHE}$ curve, the temperature-dependent AHE amplitude for different δ is summarized in **Figure S17**a, showing AHE reversal for all δ values investigated in this study. As explained in Figure 2, AHE reversal in $Cr_{1+\delta}Te_2$ occurs due to the change in sign of the k-space Berry curvature depending on the Fermi level, leading to a change in AHE polarity as the temperature varies. To determine the T_C of $\delta = 0.468$, 0.357, and 0.238, as represented in Figure S17a, the same method employed to determine the T_C of $Cr_{1.612}Te_2$ in Figure 2 is used, as detailed in SI section 1. The temperature-dependent coercivity, $H_C(T)$, extracted from ρ_{AHE} of each δ, shows that the coercivity at low temperatures increases with increasing δ, as shown by the color contour plot in Figure S17b. This indicates a behavior toward becoming magnetically harder as the $Cr_{1+\delta}Te_2$ transitions from 2D vdW FM to 3D FM: e.g., for $Cr_{1.612}Te_2$, the coercivity at 4 K reaches as high as 1.005 T, indicating a very hard magnet.

Section 3. Excluding the possibility of mimic-THE in $Cr_{1+\delta}Te_2$

3.1 Mimic-THE: two-AHE model

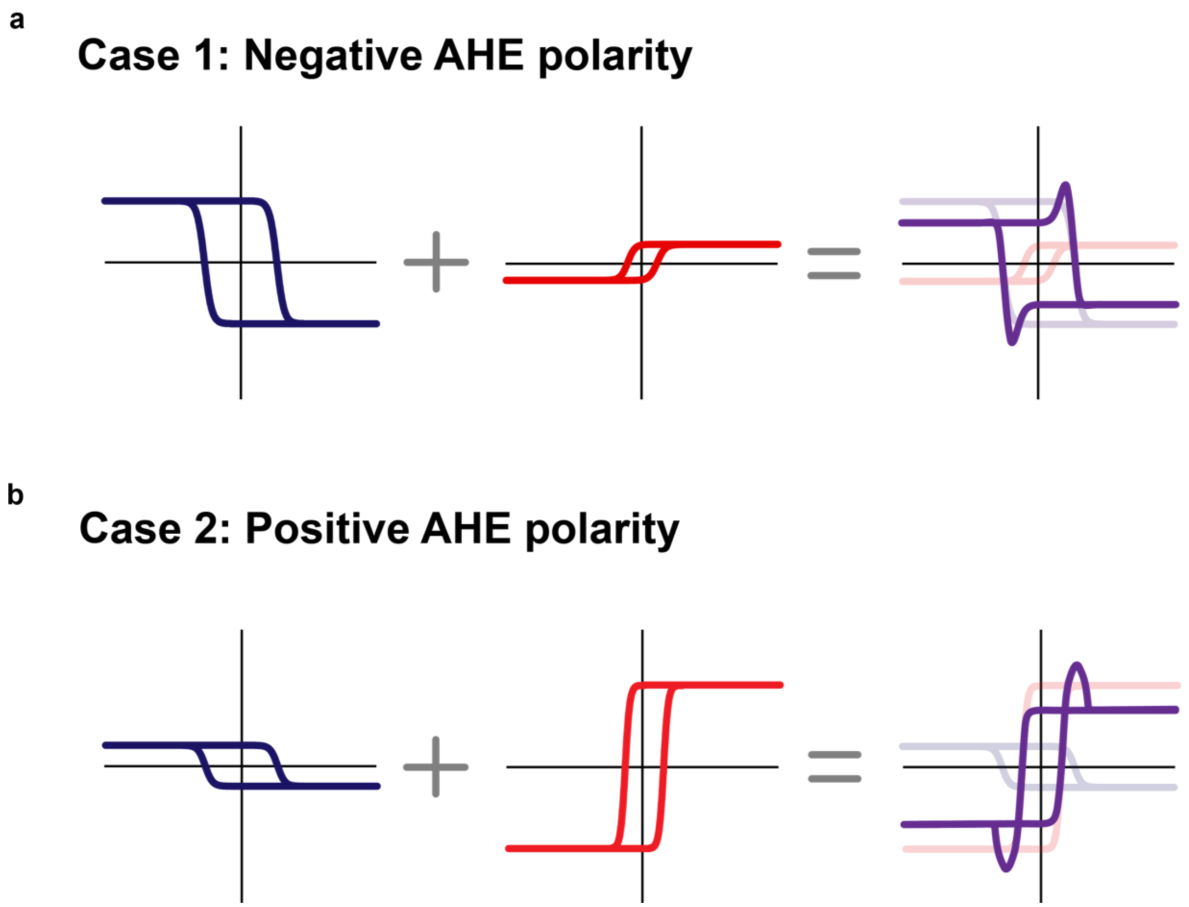

Figure S18. Schematics of two cases that can create mimic-THE: two-AHE model. a) Schematic of a case where mimic-THE can appear when the total AHE polarity is negative. b) Schematic of a case where mimic-THE can appear when the total AHE polarity is positive.

Recently, with the active research on THE in 2D FMs, the importance of reporting genuine THE has increased due to the occurrence of mimic-THE originating from other sources, such as two-AHE domain-induced mimic-THE. When there is lateral or vertical magnetic inhomogeneity in magnetic thin films, leading to two AHE domains, mimic-THE can arise under certain conditions, as depicted in the schematic in **Figure S18**. Case 1 (Figure S18a): When a

domain with large coercivity and AHE amplitude but with negative AHE polarity (domain 1) mixes with a domain with small coercivity and AHE amplitude but with positive AHE polarity (domain 2), mimic-THE with a total AHE polarity of negative can occur. Case 2 (Figure S18b): When a domain with large coercivity but small AHE amplitude and negative AHE polarity (domain 1) mixes with a domain with small coercivity but large AHE amplitude and positive AHE polarity (domain 2), mimic-THE with a total AHE polarity of positive can occur.

In this study, to prevent magnetic inhomogeneity in $Cr_{1+\delta}Te_2$ thin films, we used laterally homogeneous thin films of a certain thickness, as mentioned in the main manuscript and SI section 1.1. Moreover, there are two methods to confirm the magnetic homogeneity of magnetic materials: 1) fitting the $H_C(T)$ of the magnetic material using Kneller's law to verify if the derived parameter α is 0.5, and 2) fitting the $\rho_{xy} - \rho_{OHE}$ curve using the two-tanh function (two-AHE model) to check for any physical inconsistencies. To verify whether the pristine $Cr_{1+\delta}Te_2$ used in this work is a single magnetic domain thin film, we applied these two methods, as shown in SI section 3.2 to 3.4.

3.2 Kneller's law fitting of $H_C(T)$ for $Cr_{1+\delta}Te_2$

In SI section 3.2, to determine whether the $Cr_{1+\delta}Te_2$ used in this work has a magnetic single domain, we fitted the $H_C(T)$ of $Cr_{1+\delta}Te_2$ using Kneller's law. When fitting $H_C(T)$ with Kneller's law: $H_C(T) = H_0 \cdot [1 - \left(\frac{T}{T_B}\right)^{\alpha}]$, where H_0, T_B, α are fitting parameters, if $\alpha = 1/2$ is yielded, this indicates that the material possesses a single magnetic domain.[9,10] In other words, the material can be considered to have magnetic homogeneity. In the case of 2D FM with perpendicular magnetic anisotropy (PMA), if a chiral spin texture is formed, the net magnetization is in the out-of-plane direction, but the definition of the magnetic domain becomes

ambiguous. Therefore, while the magnitude of magnetization can be analyzed by approximating with a single-domain model, the presence of a DMI term in the magnetization energy Hamiltonian makes magnetic dynamics analysis impossible. Consequently, the $H_C(T)$ of materials with chiral spin textures may differ from that of single-domain magnetic materials and may not be fitted using Kneller's law. Therefore, we fitted the $H_C(T)$ using Kneller's law for both compositions of $Cr_{1+\delta}Te_2$ that exhibited significant THE and those that did not.

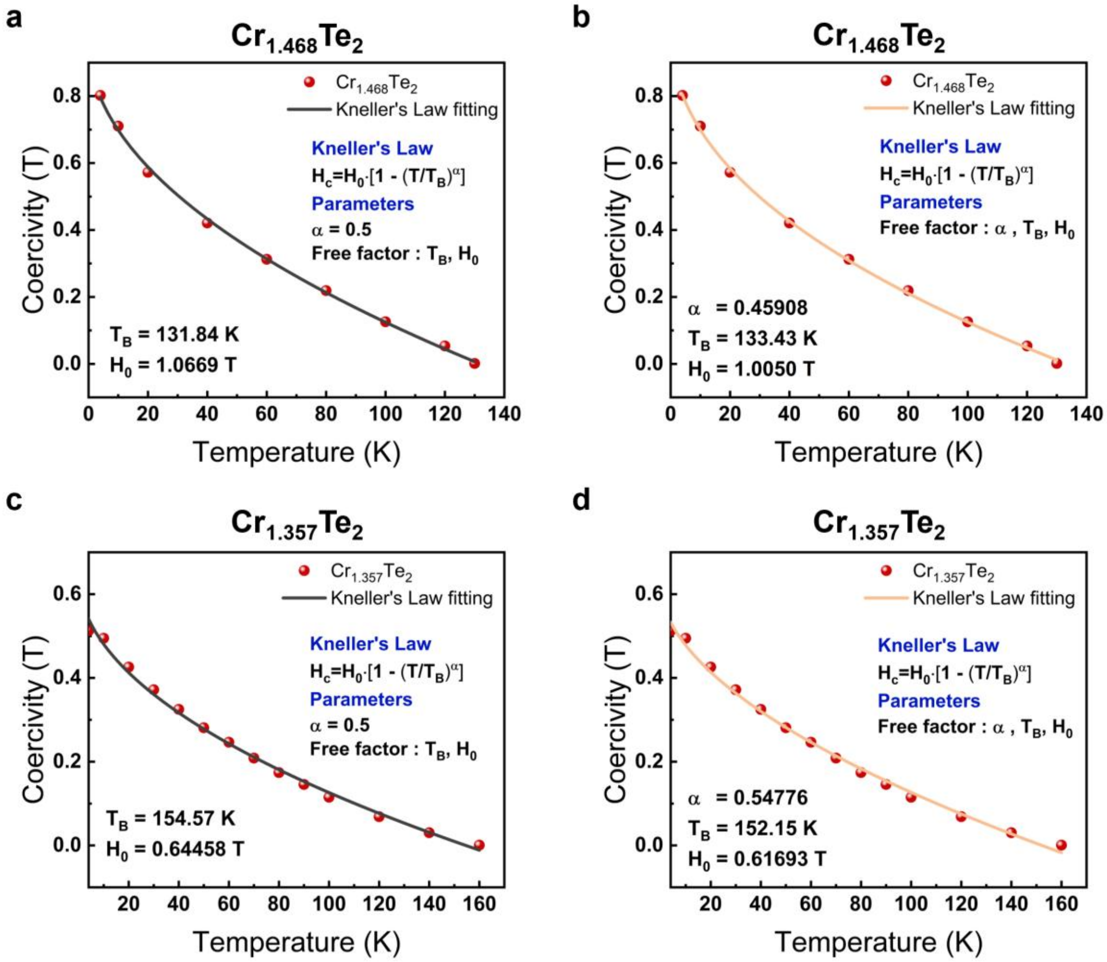

Figure S19. Kneller's law fitting of $H_C(T)$ for $Cr_{1.468}Te_2$ and $Cr_{1.357}Te_2$. a) Fitting results of $H_C(T)$ using Kneller's law for $Cr_{1.468}Te_2$ with the parameter α fixed at 1/2. b) Fitting results of $H_C(T)$ using Kneller's law for $Cr_{1.468}Te_2$ with all parameters free. c) Fitting results of $H_C(T)$

using Kneller's law for $Cr_{1.357}Te_2$ with the parameter α fixed at 1/2. d) Fitting results of $H_C(T)$ using Kneller's law for $Cr_{1.357}Te_2$ with all parameters free.

First, we fitted the $H_C(T)$ of $Cr_{1.468}Te_2$ and $Cr_{1.357}Te_2$ using Kneller's law, which did not exhibit pronounced THE, as shown in **Figure S19**. The fitting was performed in two ways: 1) H_0 and T_B as free parameters with $\alpha = 1/2$, and 2) all parameters as free. As shown in Figure S19a,c, when fitting the $H_C(T)$ of $Cr_{1.468}Te_2$ and $Cr_{1.357}Te_2$ with α fixed at 1/2, the fitting results match very well. As shown in Figure S19b,d, even when α is treated as a free parameter, the α values obtained (0.45908 and 0.54776, respectively) are close to 0.5, indicating that $Cr_{1.468}Te_2$ and $Cr_{1.357}Te_2$ possess a single magnetic domain. Therefore, $Cr_{1.468}Te_2$ and $Cr_{1.357}Te_2$ are magnetically homogeneous, and the fitting of the $H_C(T)$ using Kneller's law provides evidence that mimic-THE due to multiple AHE domains is not occurring. The parameters derived from the fitting, H_0 and T_B, represent zero-Kelvin coercivity and blocking temperature, respectively. For $Cr_{1.468}Te_2$, when α is fixed, $H_0 = 1.0669$ T, $T_B = 131.84$ K, and when α is not fixed, $H_0 = 1.0050$ T, $T_B = 133.43$ K are derived. For $Cr_{1.357}Te_2$, when α is fixed, $H_0 = 0.64458$ T, $T_B = 154.57$ K, and when α is not fixed, $H_0 = 0.61693$ T, $T_B = 152.15$ K are derived.

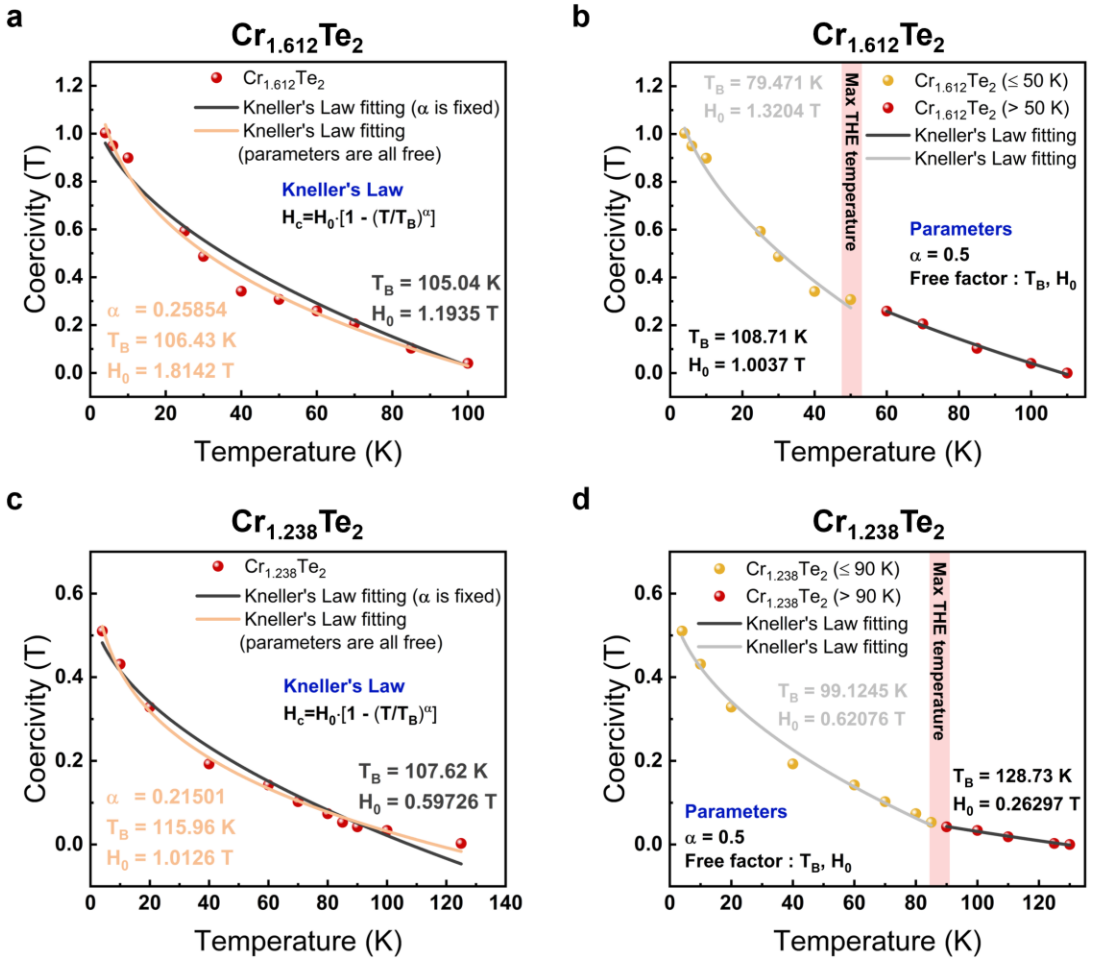

Figure S20. Kneller's law fitting of $H_C(T)$ for $Cr_{1.612}Te_2$ and $Cr_{1.238}Te_2$. a) Fitting results of $H_C(T)$ using Kneller's law for $Cr_{1.612}Te_2$ with the parameter α both fixed at 1/2 and free. b) Fitting results of $H_C(T)$ for $Cr_{1.612}Te_2$, performed by separating the regions based on the maximum temperature at which THE occurs, using Kneller's law with the parameter α fixed at 1/2. c) Fitting results of $H_C(T)$ using Kneller's law for $Cr_{1.238}Te_2$ with the parameter α both fixed at 1/2 and free. d) Fitting results of $H_C(T)$ for $Cr_{1.238}Te_2$, performed by separating the regions based on the maximum temperature at which THE occurs, using Kneller's law with the parameter α fixed at 1/2.

Then, we fitted the $H_C(T)$s of $Cr_{1.612}Te_2$ and $Cr_{1.238}Te_2$ using Kneller's law, which exhibited significant THE, as shown in **Figure S20**. We performed the Kneller's law fitting in the same manner as for $Cr_{1.468}Te_2$ and $Cr_{1.357}Te_2$. As shown in Figure S20a,c, we conducted Kneller's law fitting for the $H_C(T)$ of $Cr_{1.612}Te_2$ and $Cr_{1.238}Te_2$ with α fixed at 1/2 and as a free parameter, respectively. As mentioned earlier, if a chiral spin texture is formed, the definition of the magnetic domain becomes ambiguous, so the $H_C(T)$ would not fit well with Kneller's law. As expected, Figure S20a,c show that the $H_C(T)$ of $Cr_{1.612}Te_2$ and $Cr_{1.238}Te_2$ do not fit well using Kneller's law regardless of whether α is fixed. It is especially noticeable that the fitting does not match in the temperature range where THE appears. Moreover, when all parameters are free, the fitting results in orange curves show α values of 0.25854 ($Cr_{1.612}Te_2$), and 0.21501 ($Cr_{1.238}Te_2$), indicating that these materials are far from being magnetic single domains.

Therefore, we divided the $H_C(T)$ into two regions based on the maximum temperature at which THE appears and performed Kneller's law fitting, as shown in Figure S20b,d. Fixing α at 1/2, we fitted the $H_C(T)$ of $Cr_{1.612}Te_2$ and $Cr_{1.238}Te_2$ and found that the fitting matches well for temperatures above the maximum temperature where THE occurs (max THE temperature). Particularly for the case of $Cr_{1.612}Te_2$, the parameters derived from Kneller's law fitting are $H_0 = 1.0037$ T and $T_B = 108.71$ K, which exactly match the T_B obtained from the SQUID data in the main manuscript Figure 3c, supporting the accuracy of the fitting. For the case of $Cr_{1.238}Te_2$, fitting above the max THE temperature with α fixed at 1/2 yields $H_0 = 0.26297$ T and $T_B = 128.73$ K. However, for both $Cr_{1.612}Te_2$ and $Cr_{1.238}Te_2$, the fitting below the max THE temperature with α fixed at 1/2 does not match well, and the derived H_0 and T_B values differ significantly from experimental values, making it difficult to determine magnetic homogeneity. Thus, $Cr_{1.612}Te_2$ and $Cr_{1.238}Te_2$ are considered magnetically homogeneous at temperatures above the max THE temperature, providing evidence that multiple AHE domains do not exist at least

above the max THE temperature. This suggests that even if multiple AHE domains existed in both $Cr_{1.612}Te_2$ and $Cr_{1.238}Te_2$, they either coincidentally had T_C near the max THE temperature, becoming single domain, or were initially single domain magnetic materials. To determine whether they are single domain below the max THE temperature, we performed two-AHE model fitting on the $\rho_{xy} - \rho_{OHE}$ curves of $Cr_{1.612}Te_2$ and $Cr_{1.238}Te_2$ in SI section 3.3 to 3.4.

3.3 Two-AHE model fitting for $Cr_{1.612}Te_2$

Fitting the $\rho_{xy} - \rho_{OHE}$ curve using two tanh functions can determine whether the magnetic material has two AHE domains. If the $\rho_{xy} - \rho_{OHE}$ curve of a material exhibiting THE can be well-fitted by a two-tanh function without physical issues, THE in that material is likely due to two AHE domains. Fitting $\rho_{xy} - \rho_{OHE}$ curve data with the fitting equation $\rho_{xy} - \rho_{OHE}(H) = M_0 \tanh\left(\frac{H}{a_0} - H_{c0}\right) + M_1 \tanh\left(\frac{H}{a_1} - H_{c1}\right)$, where $M_{0,1}$, $a_{0,1}$, and $H_{c0,c1}$ are fitting parameters, will be referred to as two-AHE model fitting. Since one of the main origins of mimic-THE is the formation of two AHE domains, it is crucial to prove that a magnetic thin film has a single domain. Therefore, to verify that $Cr_{1.612}Te_2$ and $Cr_{1.238}Te_2$, which have not been confirmed as single domains, are indeed single magnetic domains, we performed two-AHE model fitting on the $\rho_{xy} - \rho_{OHE}$ curve of $Cr_{1.612}Te_2$ and $Cr_{1.238}Te_2$ (SI section 3.4).

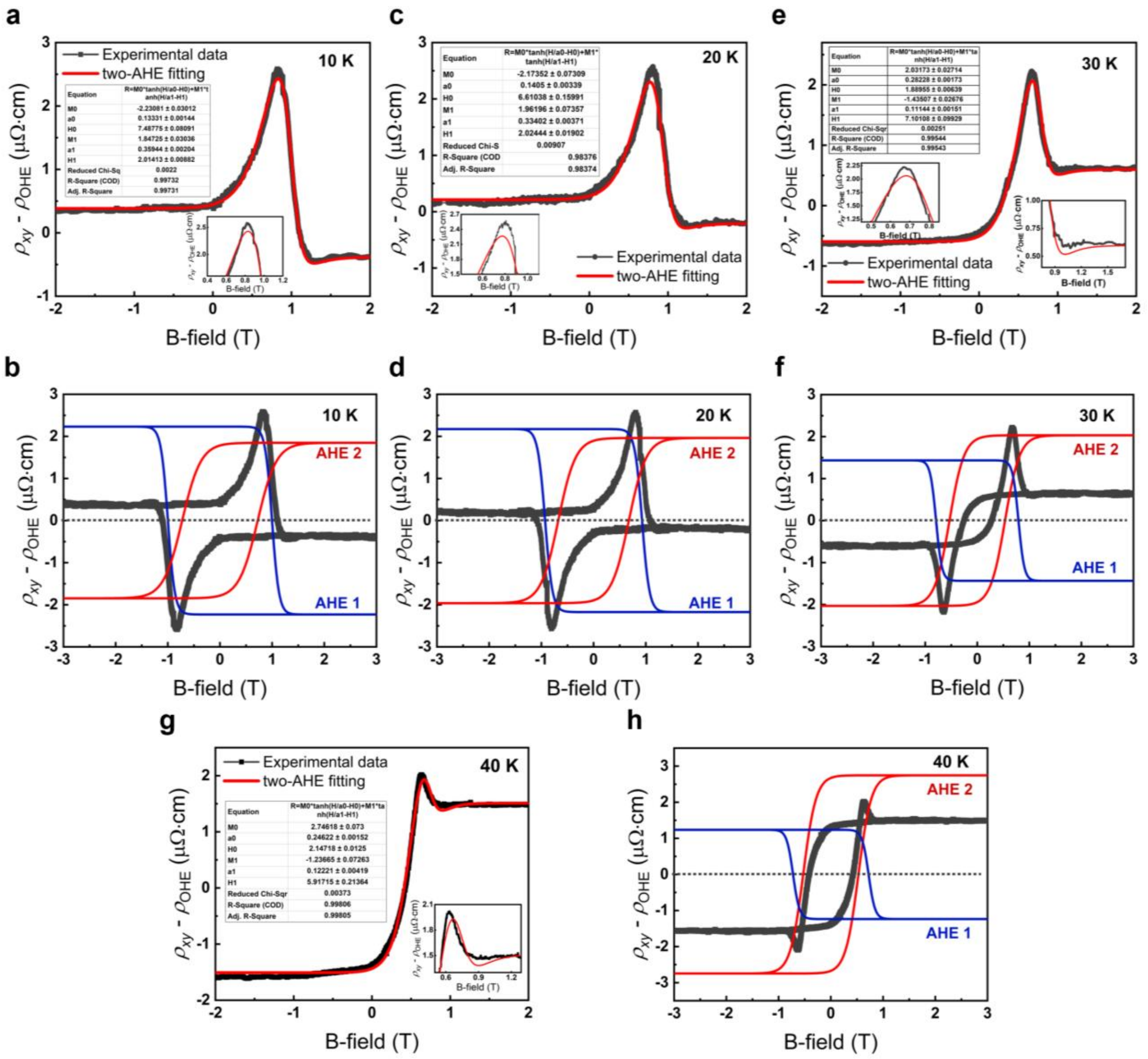

Figure S21. Two-AHE model fitting of $\rho_{xy} - \rho_{OHE}$ curve for $Cr_{1.612}Te_2$. a, c, e, g) Two-AHE model fitting results of the $\rho_{xy} - \rho_{OHE}$ curve at 10, 20, 30, and 40 K, respectively. The fitting results (experimental data for the $\rho_{xy} - \rho_{OHE}$ curve) are represented by the red solid line (gray solid line with gray dots). Each inset: Enlarged curve data near the THE peak or where the fitting is not accurate. Each inset table: A table of parameters derived from the fitting using the two-tanh function (two-AHE model). b, d, f, h) Two-AHE domains obtained from the two-AHE model fitting of the $\rho_{xy} - \rho_{OHE}$ curve at 10, 20, 30, and 40 K, respectively. Each AHE domain

is represented by red and blue solid lines, and the experimental data of the $\rho_{xy} - \rho_{OHE}$ curve is represented by the gray solid line with gray dots.

As shown in **Figure S21**, the results of fitting the $\rho_{xy} - \rho_{OHE}$ curve of $Cr_{1.612}Te_2$ with the two-AHE model for 10, 20, 30, and 40 K are presented. The insets include the derived fitting parameters and enlarged data for regions where the fitting is not accurate. As mentioned in SI section 3.1, the fitting can be done using two AHE loops with different coercivities and AHE polarities. The two-AHE domains (AHE 1 and AHE 2) obtained from the two-AHE model fitting of the $\rho_{xy} - \rho_{OHE}$ curve at 10, 20, 30, and 40 K are shown in Figure S21b,d,f, and h, respectively. Fortunately, the $\rho_{xy} - \rho_{OHE}$ curve data of $Cr_{1.612}Te_2$ from 10 to 40 K appear to be generally well-fitted by the two-AHE model. However, there are two issues: 1) Near the peak of the hump-like signal, it is impossible to fit accurately with any combination of $M_{0,1}, H_{0,1},$ and $a_{0,1}$ fitting parameters. 2) The fitting is inaccurate near the end of the hump-like signal. For example, in the insets of the $\rho_{xy} - \rho_{OHE}$ curve data at 30 K (Figure S21e) and 40 K (Figure S21g), the two-AHE model fitting results appear concave near the end of the THE signal. However, a $\rho_{xy} - \rho_{OHE}$ curve composed of one ρ_{AHE} loop and ρ_{THE} should not have an inflection point near the end of the THE signal. This phenomenon occurs because the $\rho_{xy} - \rho_{OHE}$ curve resulting from one AHE domain is being forcibly fitted with the two-tanh function. Therefore, $Cr_{1.612}Te_2$ is expected to have one AHE domain under 40 K.

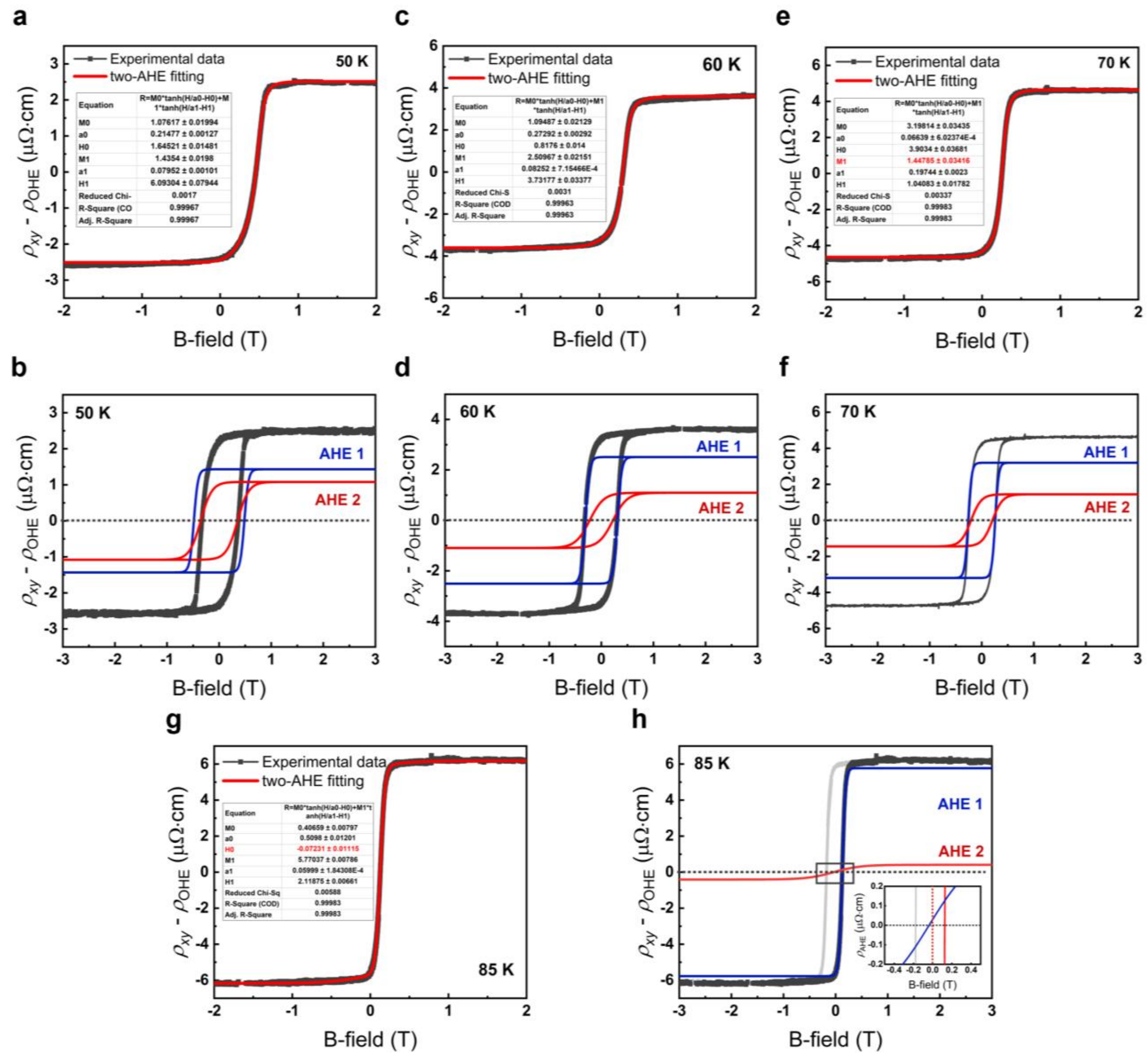

Figure S22. Two-AHE model fitting of $\rho_{xy} - \rho_{OHE}$ curve for $Cr_{1.612}Te_2$. a, c, e, g) Two-AHE model fitting results of the $\rho_{xy} - \rho_{OHE}$ curve at 50, 60, 70, and 85 K, respectively. The fitting results (experimental data for the $\rho_{xy} - \rho_{OHE}$ curve) are represented by the red solid line (gray solid line with dray dots). Each inset table: A table of parameters derived from the fitting using the two-tanh function (two-AHE model), with physically impossible values highlighted in red. b, d, f, h) Two-AHE domains obtained from the two-AHE model fitting of the $\rho_{xy} - \rho_{OHE}$ curve at 50, 60, 70, and 85 K, respectively. Each AHE domain is represented by red and blue solid lines,

and the experimental data of the $\rho_{xy} - \rho_{OHE}$ curve is represented by the gray solid line with gray dots. Inset of (**h**): Enlarged curve data near the origin. Since the AHE loop in the positive sweep has a negative coercivity, this indicates a physically impossible result.

As shown in **Figure S22**, the fitting results of the $\rho_{xy} - \rho_{OHE}$ curve of $Cr_{1.612}Te_2$ using the two-AHE model at 50, 60, 70, and 85 K are presented. The insets include the derived fitting parameters and enlarged data for regions where the fitting is inaccurate. In the $\rho_{xy} - \rho_{OHE}$ curve above 50 K, the THE signal is negligible, and the AHE polarity is positive. One thing to note is that if one AHE loop perfectly follows a tanh function, it can always be fitted by the two-AHE model due to the characteristics of the tanh function. Therefore, it is crucial to check whether the physical parameters obtained from the fitting are reasonable. The values of $M_{0,1}$ obtained from the two-AHE model fitting represent the ρ_{AHE} of each domain, while $H_{0,1} \cdot a_{0,1}$ represent the coercivity of each domain. Since the values obtained at 40 K are $M_{0,1} = -1.237, 2.746$, for the total AHE polarity to be positive at 50 K, one domain (in this case, AHE 1 obtained from the two-AHE fitting, represented by the blue solid line) would need to have a T_C between 40 and 50 K, or a rapid transition from negative to positive AHE polarity would be required in this temperature range. The former is unlikely for homogeneous $Cr_{1.612}Te_2$ with a T_C of approximately 195 K, so the fitting was performed assuming the latter. As expected, a rapid change in AHE polarity for AHE 1 was observed at 50 K through the two-AHE model fitting. However, physical errors can be detected when fitting across different temperatures. For example, 1) the ρ_{AHE} of AHE 2 obtained from the two-AHE model fitting is not monotonic as a function of temperature, and 2) the coercivity of AHE 2 obtained from the two-AHE model fitting of the $\rho_{xy} - \rho_{OHE}$ curve at 85 K is negative for the positive sweep (Figure S22g,h). Therefore, fitting

the $\rho_{xy} - \rho_{OHE}$ curve of $Cr_{1.612}Te_2$ using the two-AHE model appears to be incorrect, suggesting that $Cr_{1.612}Te_2$ 7.6 nm has one AHE domain across the entire temperature range.

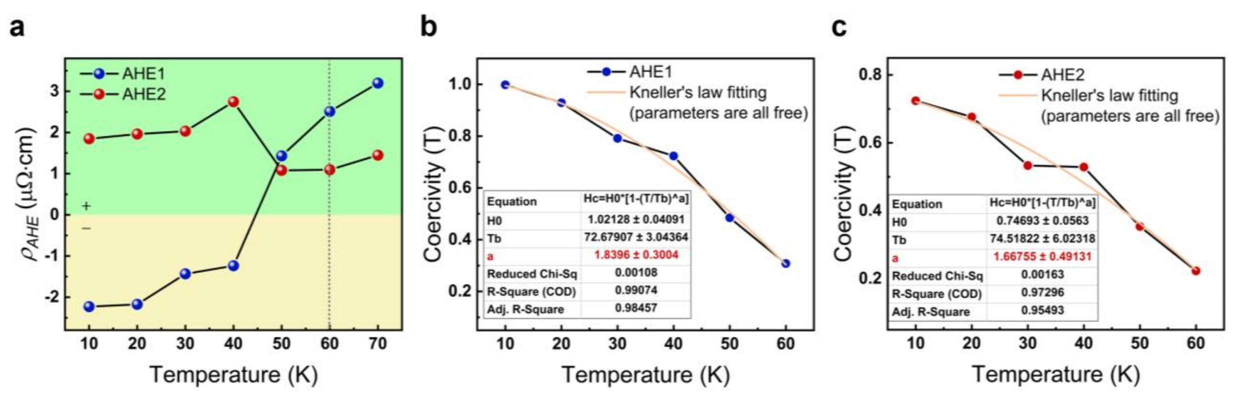

Figure S23. Summary of the two-AHE model fitting results for $Cr_{1.612}Te_2$. a) Temperature-dependent AHE amplitude for each of the two AHE domains obtained from the two-AHE model fitting. b) $H_C(T)$ for the first AHE domain and the fitting results using Kneller's law with all parameters free. c) $H_C(T)$ for the second AHE domain and the fitting results using Kneller's law with all parameters free. Insets of (b) and (c): Tables of parameters derived from the fitting using Kneller's law. Physically impossible values are highlighted in red.

As shown in **Figure S23**, the ρ_{AHE} and $H_C(T)$ of AHE 1 and AHE 2 obtained through the two-AHE model fitting in Figure S21 and S22 are summarized. In Figure S23a, the AHE amplitudes of AHE 1 and AHE 2 obtained from the two-AHE model fitting of $Cr_{1.612}Te_2$ are represented. The physically impossible phenomena occurring in AHE 2, as explained in Figure S22, are clearly shown. The temperature at which these phenomena start is indicated by a gray dotted line. Since AHE amplitude is proportional to magnetization, it should be monotonic with temperature. Moreover, when AHE arises from an intrinsic origin, the AHE amplitude and polarity are proportional to the k-space Berry curvature. However, 1) the AHE 1 domain shows a

rapid change in AHE amplitude and a polarity within a 10 K range, and 2) the AHE amplitude of the AHE 2 domain is not monotonic with temperature. This indicates an error in fitting the $\rho_{xy} - \rho_{OHE}$ curve of $Cr_{1.612}Te_2$ using the two-AHE model.

To further verify, we fitted the $H_C(T)$ using Kneller's law for AHE 1 and AHE 2 obtained from the two-AHE model fitting, as shown in Figure S23b, c. When fitting both domains with all parameters free, the α values derived (1.8396 and 1.6676, respectively) are far from those of a single domain, and the fitting itself is not accurate. Furthermore, the derived T_B values (72.68 and 74.52 K, respectively) are significantly different from the T_B of 108 K measured by SQUID, indicating a lack of agreement with experimental values. In conclusion, it is impossible to fit the $\rho_{xy} - \rho_{OHE}$ curve of $Cr_{1.612}Te_2$ assuming two AHE domains, indicating that $Cr_{1.612}Te_2$ has a single magnetic domain at all temperatures. Therefore, the THE observed in $Cr_{1.612}Te_2$ is due to real space Berry curvature, not multiple AHE domains.

3.4 Two-AHE model fitting for $Cr_{1.238}Te_2$

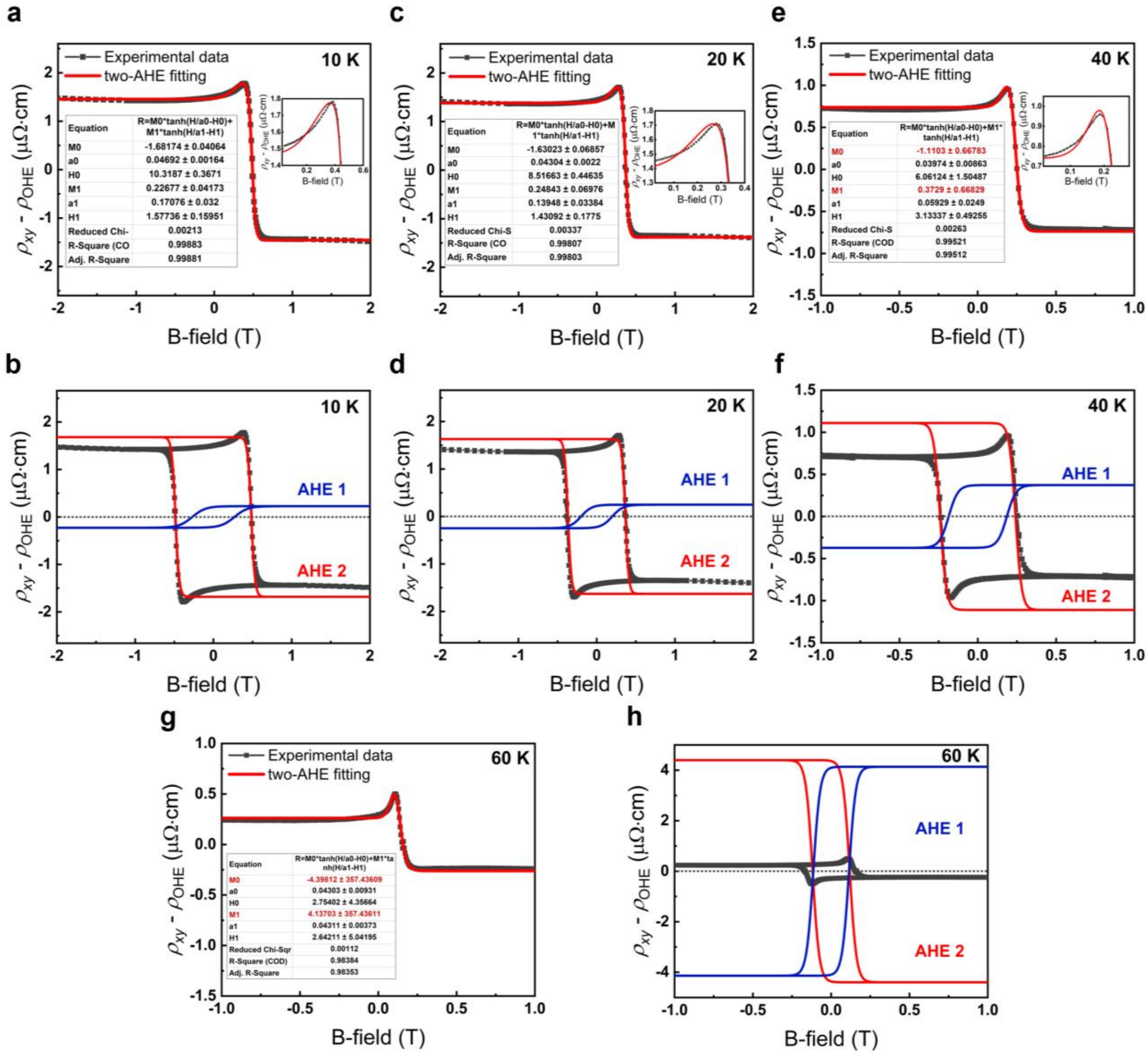

Figure S24. Two-AHE model fitting of $\rho_{xy} - \rho_{OHE}$ curve for $Cr_{1.238}Te_2$. a, c, e, g) Two-AHE model fitting results of the $\rho_{xy} - \rho_{OHE}$ curve at 10, 20, 40, and 60 K respectively. The fitting results (experimental data for the $\rho_{xy} - \rho_{OHE}$ curve) are represented by the red solid line (gray solid line with gray dots). Each inset table: A table of parameters derived from the fitting using the two-tanh function (two-AHE model). Physically impossible values are highlighted in red. b, d, f, h) Two-AHE domains obtained from the two-AHE model fitting of the $\rho_{xy} - \rho_{OHE}$ curve at

10, 20, 40, and 60 K, respectively. Each AHE domain is represented by red and blue solid lines, and the experimental data of the $\rho_{xy} - \rho_{OHE}$ curve is represented by the gray solid line with gray dots.

As shown in **Figure S24**, the fitting results of the $\rho_{xy} - \rho_{OHE}$ curve of $Cr_{1.238}Te_2$ using the two-AHE model at 10, 20, 40, and 60 K are presented. The insets include the derived fitting parameters and enlarged data for regions where the fitting is inaccurate. Unlike $Cr_{1.612}Te_2$, the two-AHE model fitting of the $\rho_{xy} - \rho_{OHE}$ curve for $Cr_{1.238}Te_2$ did not converge for all temperatures unless one domain parameter was fixed arbitrarily and then allowed to vary freely during the fitting process. Similar to $Cr_{1.612}Te_2$, the $\rho_{xy} - \rho_{OHE}$ curve of $Cr_{1.238}Te_2$ also exhibited inaccuracies in fitting near the peak of the hump-like signal. Moreover, the fitting errors were significantly larger than the $M_{0,1}$ values obtained from fitting at temperatures above 20 K, as shown in the insets of Figure S24e and g. These physical errors are highlighted in red and bold in the tables within each inset. Notably, the AHE amplitude of each domain derived from two-AHE model fitting for the AHE amplitude of each domain derived from the two-AHE model fitting of $\rho_{xy} - \rho_{OHE}$ curve for $Cr_{1.238}Te_2$ at 60 K appeared excessively large compared to the experimental data (approximately five times larger). Therefore, fitting $\rho_{xy} - \rho_{OHE}$ curve of $Cr_{1.238}Te_2$ using the two-AHE model is physically inappropriate, suggesting that $Cr_{1.238}Te_2$ possesses a single AHE domain.

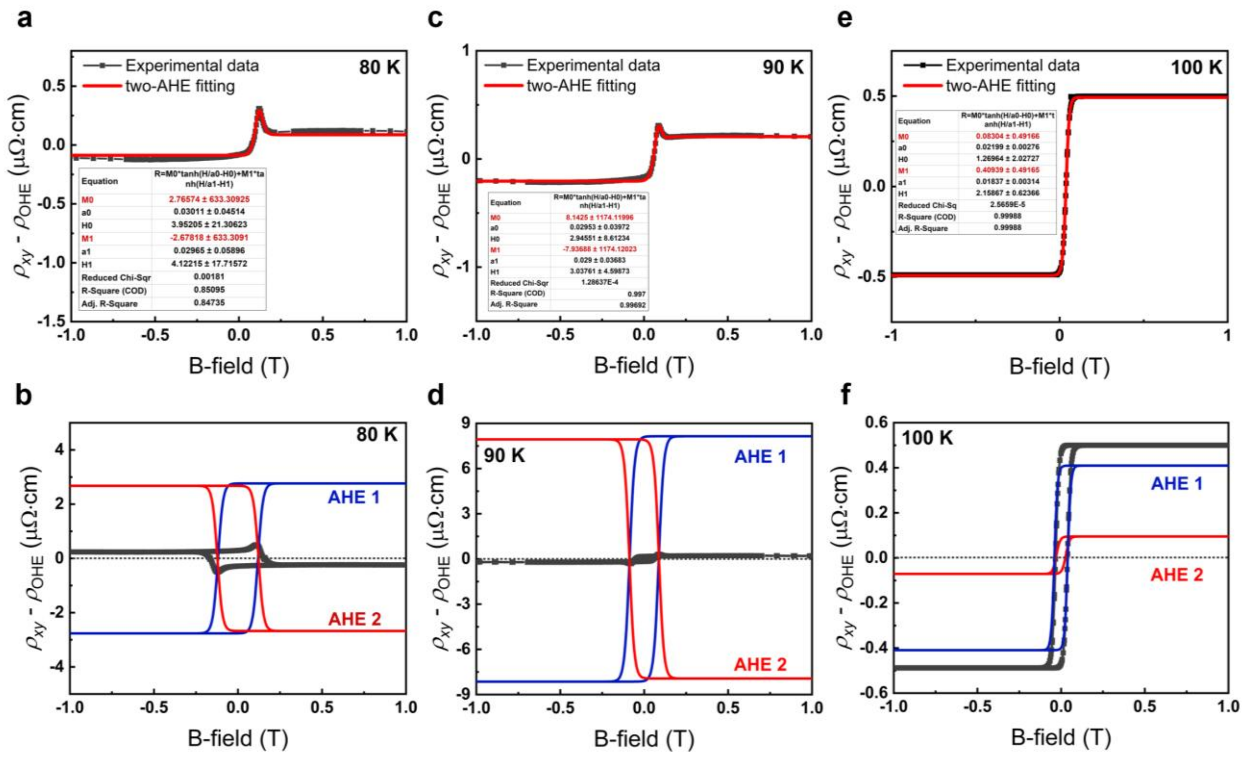

Figure S25. Two-AHE model fitting of $\rho_{xy} - \rho_{OHE}$ curve for $Cr_{1.238}Te_2$. a, c, e) Two-AHE model fitting results of the $\rho_{xy} - \rho_{OHE}$ curve at 80, 90, and 100 K, respectively. The fitting results (experimental data for the $\rho_{xy} - \rho_{OHE}$ curve) are represented by the red solid line (gray solid line with gray dots). Each inset table: A table of parameters derived from the fitting using the two-tanh function (two-AHE model). Physically impossible values are highlighted in red. b, d, f) Two-AHE domains obtained from the two-AHE model fitting of the $\rho_{xy} - \rho_{OHE}$ curve at 80, 90, and 100 K, respectively. Each AHE domain is represented by red and blue solid lines, and the experimental data of the $\rho_{xy} - \rho_{OHE}$ curve is represented by the gray solid line with gray dots.

Nevertheless, to verify more accurately, we conducted two-AHE model fitting for 80, 90, and 100 K, as shown in **Figure S25**. However, the $M_{0,1}$ values obtained from fitting at these

temperatures were even more physically unreasonable than those derived from fitting data below 60 K, as shown in Figure S25a,c,e and the inset tables. Similar to the fitting results of the $\rho_{xy} - \rho_{OHE}$ curve for $Cr_{1.612}Te_2$, the AHE amplitudes of the two AHE domains obtained from the two-AHE model fitting of the $\rho_{xy} - \rho_{OHE}$ curve for $Cr_{1.238}Te_2$ are non-monotonic with respect to temperature. Therefore, it is expected that $Cr_{1.238}Te_2$ 8 nm has a single AHE domain across all temperature ranges.

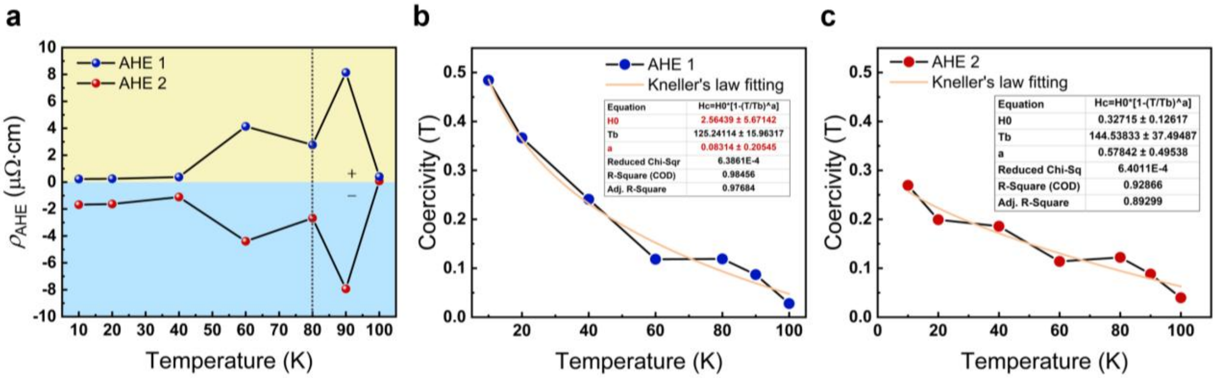

Figure S26. Summary of the two-AHE model fitting results for $Cr_{1.238}Te_2$. a) Temperature-dependent AHE amplitude for each of the two AHE domains obtained from the two-AHE model fitting. b) $H_C(T)$ for the first AHE domain and the fitting results using Kneller's law with all parameters free. c) $H_C(T)$ for the second AHE domain and the fitting results using Kneller's law with all parameters free. Insets of (b) and (c): Tables of parameters derived from the fitting using Kneller's law. Physically impossible values are highlighted in red.

As shown in **Figure S26**, the ρ_{AHE} and $H_C(T)$ of AHE 1 and AHE 2 obtained through the two-AHE model fitting in Figure S24 and S25 are summarized. As shown in Figure S26a, the AHE amplitudes of AHE 1 and AHE 2 obtained from the two-AHE model fitting of $Cr_{1.238}Te_2$ are represented, and it is observed that the AHE amplitudes of both AHE 1 and AHE 2 fluctuate

significantly and are non-monotonic with respect to temperature. The temperature at which physically impossible phenomena begin is indicated by a gray-dotted line, but the fitting itself already does not converge at temperatures above 20 K. Nevertheless, we fitted the $H_C(T)$ of AHE 1 and AHE 2 obtained from the two-AHE model fitting using Kneller's law, as shown in Figure S26b, c. When fitting both domains with all parameters free, the α values derived (2.5439 and 0.32715, respectively) are far from that expected for a single domain, and the fitting itself is inaccurate. In conclusion, it is impossible to fit the $\rho_{xy} - \rho_{OHE}$ curve of $Cr_{1.238}Te_2$ assuming two AHE domains, indicating that $Cr_{1.238}Te_2$ has a single magnetic domain at all temperatures. Therefore, the THE observed in $Cr_{1.238}Te_2$ is due to real space Berry curvature, not multiple AHE domains.

Section 4. Chiral spin texture in $Cr_{1.612}Te_2$

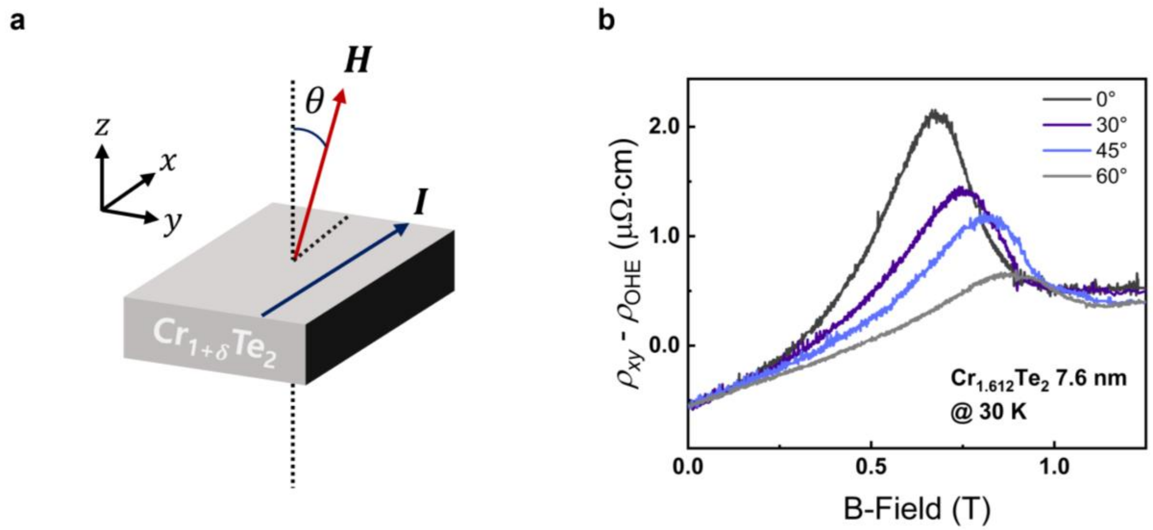

Figure S27. B-field angle-dependent THE in $Cr_{1.612}Te_2$. a) Schematic of the measurement setup for the B-field angle-dependent THE. An electrical current is applied along the $\hat{x}$ direction. θ represents the angle between the B-field and the z-axis in the z-x plane. THE signal was

measured by varying θ. b) $\rho_{xy} - \rho_{OHE}$ curve data of $Cr_{1.612}Te_2$ 7.6 nm with $\theta = 0°, 30°, 45°, 60°$ at 30 K.

To investigate the specific chiral spin texture that gives rise to THE in $Cr_{1.612}Te_2$, we measured the B-field angle-dependent THE. As shown in **Figure S27**a, θ is defined as the angle between the z-axis and the direction of the applied electrical current ($\hat{x}$ direction). We then applied a magnetic field at an angle θ with respect to the z-axis and measured the $\rho_{xy} - \rho_{OHE}$ curve. The B-field angle dependence of THE in magnetic materials can reveal the type of chiral spin texture according to the results. For instance, when THE is induced by the formation of skyrmions, the THE signal disappears when the $\rho_{xy} - \rho_{OHE}$ curve is measured with a B-field angle of $\theta = 10°$ (as reported in EuO[11] and $Mn_{1-x}Fe_xSi$[12]). However, for large-scale non-coplanar spin textures, the THE signal persists even with a B-field angle of $\theta > 60°$, showing a stark contrast to 2D skyrmions.[4]

Therefore, we measured the B-field angle-dependent THE for $Cr_{1.612}Te_2$ 7.6 nm at 30 K, which exhibited a large THE amplitude in this study, as shown in Figure S27b. The $\rho_{xy} - \rho_{OHE}$ curve of $Cr_{1.612}Te_2$ was measured at B-field angles of $\theta = 0°, 30°, 45°, 60°$. Even at $\theta = 60°$, a slight THE signal remains, indicating that $Cr_{1.612}Te_2$ has a non-coplanar spin texture at temperatures below 50 K, where THE manifests.

Section 5. Non-monotonic temperature-dependent THE amplitude in $Cr_{1.612}Te_2$

a

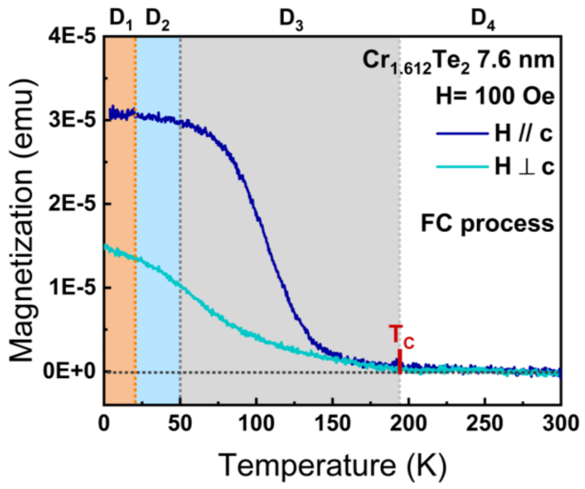

Figure S28. SQUID measurement data of $Cr_{1.612}Te_2$ 7.6 nm, performed with an applied magnetic field parallel and perpendicular to the c-axis. a) *M*–*T* curve data of $Cr_{1.612}Te_2$ under FC process with a 100 Oe magnetic field applied parallel and perpendicular to the c-axis. Temperature domains in (a) are divided using orange, sky blue, gray, and white colors, and are indicated as D_1, D_2, D_3, and D_4, respectively.

To understand only $Cr_{1.612}Te_2$ exhibits a non-monotonic temperature-dependent THE amplitude among the $Cr_{1+\delta}Te_2$ films used in this study, SQUID measurements were performed with a 100 Oe magnetic field applied parallel and perpendicular to the c-axis under FC process. As shown in **Figure S28**a, both the in-plane (blue solid line) and out-of-plane (cyan solid line) *M*–*T* curve data demonstrate that the T_C of $Cr_{1.612}Te_2$ is consistent with 195 K. Temperature domains exhibiting distinct THE and magnetic properties are delineated using various colors, with each domain labeled as D_1, D_2, D_3, and D_4. Explaining the domains in descending order of temperature: the D_4 domain exists above T_C, where $Cr_{1.612}Te_2$ loses its magnetic properties. The

D_3 domain lies below the T_C of $Cr_{1.612}Te_2$, demonstrating ferromagnetic ordering where magnetization is observable via SQUID measurements. Previous studies have reported that $Cr_{1+\delta}Te_2$ films with $\delta < 0.6$ exhibit strong perpendicular magnetic anisotropy (PMA), while for $\delta > 0.6$, PMA weakens, suggesting the emergence of in-plane magnetic anisotropy.[13] Consistent with these findings, in the D_3 domain, although out-of-plane magnetization is stronger, the in-plane magnetization is also substantial. Near the end temperature of the D_3 domain at 50 K, out-of-plane magnetization approaches saturation. The D_2 domain spans from the onset temperature of THE in $Cr_{1.612}Te_2$ to the temperature at which ρ_{THE}^{peak} reaches its maximum at 20 K. Out-of-plane magnetization remains relatively constant with temperature, while in-plane magnetization increases as temperature decreases in the D_2 domain. This suggests that starting from the D_2 domain, the ratio of in-plane to out-of-plane magnetization increases, indicating the onset of PMA weakening. The D_1 domain corresponds to the temperature range where the ratio of in-plane magnetization to out-of-plane magnetization increases to approximately 45%. Consequently, ρ_{THE}^{peak} of $Cr_{1.612}Te_2$ decreases as temperature decreases due to further weakening of PMA in the range of the D_1 domain.

Since THE arises from the chiral spin texture generating Ω_r when a magnetic field is applied parallel to the c-axis, spin texture significantly influences the THE properties. Moreover, spin texture is greatly affected by magnetic anisotropy. Recent studies have also highlighted the impact of magnetic anisotropy on THE, emphasizing its role in creating non-coplanar spin texture.[14] Given that $Cr_{1.612}Te_2$ exhibits a non-coplanar spin texture, as demonstrated in SI section 4, the reason for the decrease in ρ_{THE}^{peak} in the D_1 domain can be attributed to the weakening of magnetic anisotropy.

Section 6. The ρ_{xy} of lateral Cr_2Te_3/$CrTe_2$ with broken inversion symmetry at the interface

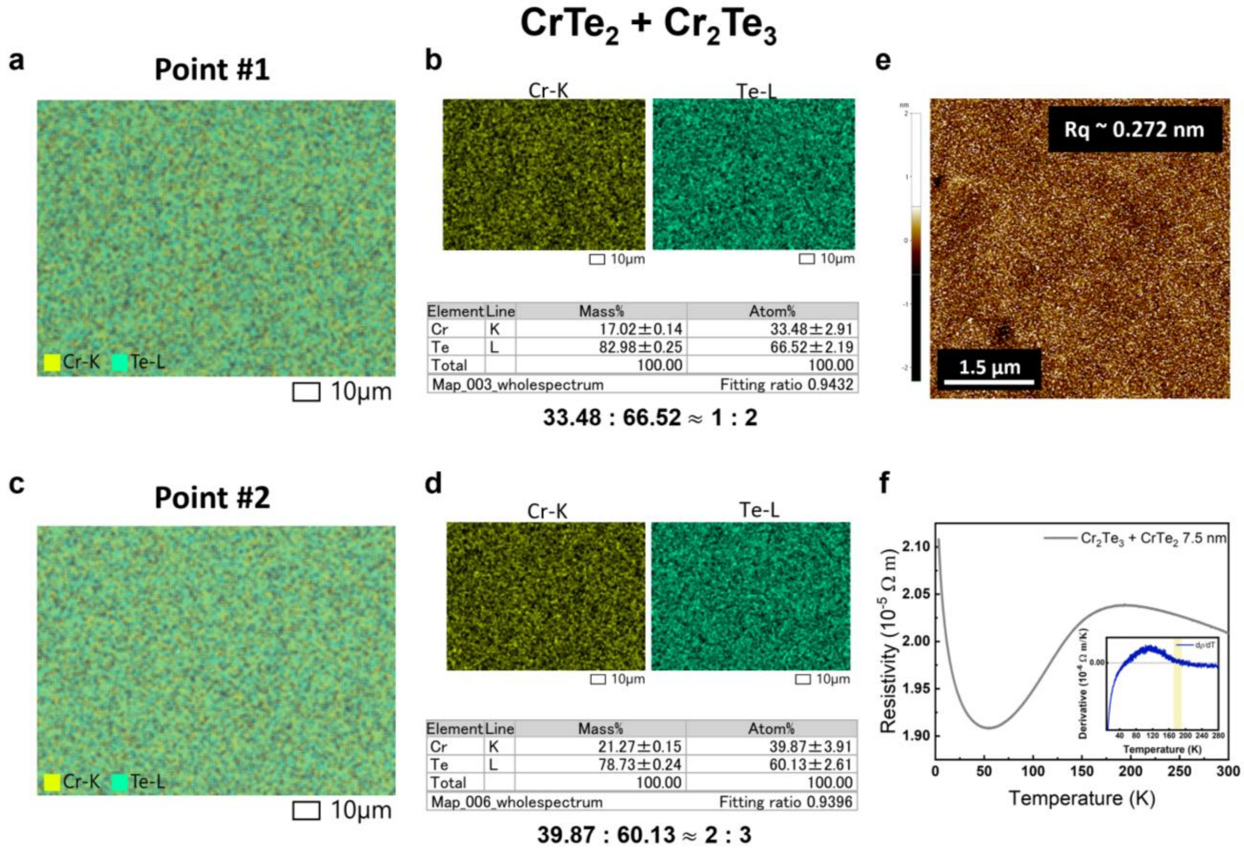

Figure S29. EDS, AFM data, and *R*–*T* curve data of Cr_2Te_3 + $CrTe_2$ 7.5 nm. a) Top-view EDS layered image of point #1 in Cr_2Te_3 + $CrTe_2$. b) EDS elemental mapping of Cr_2Te_3 + $CrTe_2$ (top-view) with 10 μm scale bar. Atomic percentages of Cr and Te are calculated based on the K and L series spectrum respectively. c) Top-view EDS layered image of point #2 in Cr_2Te_3 + $CrTe_2$. d) EDS elemental mapping of Cr_2Te_3 + $CrTe_2$ (top-view) with 10 μm scale bar. e) AFM image of Cr_2Te_3 + $CrTe_2$ (with AlO_x capping) with 1.5 μm scale bar. f) *R*–*T* curve data of Cr_2Te_3 + $CrTe_2$. Inset: First-order derivative of the *R*–*T* curve.

The EDS layered images of the two regions for Cr_2Te_3 + $CrTe_2$ 7.5 nm are shown in **Figure S29**a and c. For this sample, two regions near the transport channel of the Hall bar device were

analyzed, as indicated in **Figure S30**a. The EDS elemental mapping and atomic concentration information are provided in Figure S29b and d. The atomic concentration for the first region was determined to be Cr:Te = 1:2, while for the second region, it was determined to be Cr:Te = 2:3. Therefore, the sample shown in Figure S29 is considered laterally inhomogeneous, consisting of Cr_2Te_3 and $CrTe_2$.

AFM measurements were conducted to measure the surface flatness of Cr_2Te_3 + $CrTe_2$ with an AlO_x capping layer, as shown in Figure S29e. The Cr_2Te_3 + $CrTe_2$ 7.5 nm film with an AlO_x 2 nm capping layer exhibits flat surfaces with a surface roughness of approximately 0.272 nm. The *R*–*T* curve of Cr_2Te_3 + $CrTe_2$ is presented in Figure S29f, showing ferromagnetic transition near 190 K. Moreover, the first derivative of the *R*–*T* curve changes sharply between 180 K and 200 K, indicating that the T_C of Cr_2Te_3 + $CrTe_2$ is approximately 190 K, as shown in the inset of Figure S29f. The resistivity of Cr_2Te_3 + $CrTe_2$ at 300 K is confirmed to be approximately $2.01\times 10^{-5}\ \Omega\cdot m$.

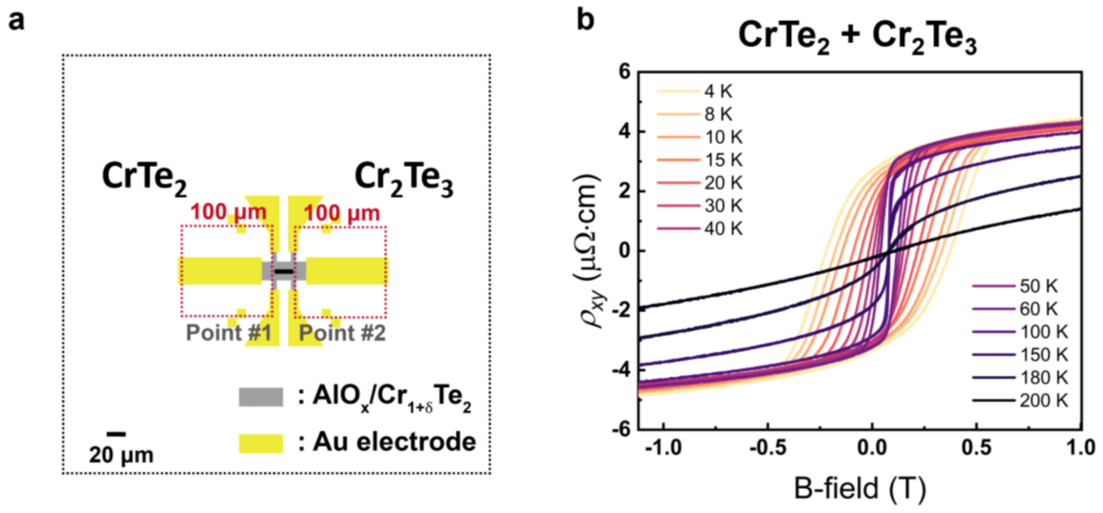

Figure S30. Negligible THE in Cr_2Te_3 + $CrTe_2$ 7.5 nm. a) Schematic image of the two regions selected for EDS elemental mapping measurement of $CrTe_2$ + Cr_2Te_3 7.5 nm. b) ρ_{xy} as a function of the B-field with different temperatures for $CrTe_2$ + Cr_2Te_3 7.5 nm.

When measuring the atomic concentration of the sample represented in Figure S29, adopting the region elucidated in Figure S1, we identified that the sample was grown in an inhomogeneous manner. Therefore, we selected two regions near the transport channel of the Hall bar device, as shown in Figure S30a, which significantly influence ρ_{xy} curve, and measured their atomic concentrations. Consequently, we identified that $CrTe_2$ and Cr_2Te_3 were laterally distributed near the transport channel, as confirmed by the EDS data in Figure S29a–d. The laterally inhomogeneous $CrTe_2$ + Cr_2Te_3 7.5 nm sample exhibited a negligible THE contribution in the temperature-dependent ρ_{xy} curve, as shown in Figure S30b. This is attributed to the centrosymmetric characteristic of both $CrTe_2$ and Cr_2Te_3, which exhibit zero bulk DMI strength, resulting in negligible THE, since the THE of pristine $Cr_{1+\delta}Te_2$ is dominated by the bulk DMI strength. Therefore, lateral symmetry breaking cannot generate an effective THE or Ω_r.

References

[1] J. Yang, C. Zhu, Y. Deng, B. Tang, Z. Liu, *iScience* **2023**, *26*, 106567.

[2] K. Niu, G. Qiu, C. Wang, D. Li, Y. Niu, S. Li, L. Kang, Y. Cai, M. Han, J. Lin, *Adv. Funct. Mater.* **2023**, *33*, 2208528.

[3] X. Zhang, S. C. Ambhire, Q. Lu, W. Niu, J. Cook, J. S. Jiang, D. Hong, L. Alahmed, L. He, R. Zhang, *ACS Nano* **2021**, *15*, 15710.

[4] Y. Chen, Y. Zhu, R. Lin, W. Niu, R. Liu, W. Zhuang, X. Zhang, J. Liang, W. Sun, Z. Chen, *Adv. Funct. Mater.* **2023**, *33*, 2302984.

[5] W. Wang, M. W. Daniels, Z. Liao, Y. Zhao, J. Wang, G. Koster, G. Rijnders, C.-Z. Chang, D. Xiao, W. Wu, *Nat. Mater.* **2019**, *18*, 1054.

[6] J. Matsuno, N. Ogawa, K. Yasuda, F. Kagawa, W. Koshibae, N. Nagaosa, Y. Tokura, M. Kawasaki, *Sci. Adv.* **2016**, *2*, e1600304.

[7] Y. Ohuchi, J. Matsuno, N. Ogawa, Y. Kozuka, M. Uchida, Y. Tokura, M. Kawasaki, *Nat. Commun.* **2018**, *9*, 213.

[8] L. Wang, Q. Feng, Y. Kim, R. Kim, K. H. Lee, S. D. Pollard, Y. J. Shin, H. Zhou, W. Peng, D. Lee, *Nat. Mater.* **2018**, *17*, 1087.

[9] N. S. E. Osman, T. Moyo, *J. Supercond. Nov. Magn.* **2016**, *29*, 361.

[10] K. Maaz, A. Mumtaz, S. Hasanain, M. Bertino, *J. Magn. Magn. Mater.* **2010**, *322*, 2199.

[11] D. C. Ramirez, T. Besara, J. B. Whalen, T. Siegrist, *Phys. Rev. B* **2017**, *95*, 014407.

[12] T. Yokouchi, N. Kanazawa, A. Tsukazaki, Y. Kozuka, M. Kawasaki, M. Ichikawa, F. Kagawa, Y. Tokura, *Phys. Rev. B* **2014**, *89*, 064416.

[13] C. Zhang, C. Liu, J. Zhang, Y. Yuan, Y. Wen, Y. Li, D. Zheng, Q. Zhang, Z. Hou, G. Yin, *Adv. Mater.* **2023**, *35*, 2205967.

[14] S. Purwar, S. Changdar, S. Ghosh, T. K. Bhowmik, S. Thirupathaiah, *Acta Mater.* **2024**, *271*, 119898.
